# Epithelial cell size dysregulation in human lung adenocarcinoma

**DOI:** 10.1371/journal.pone.0274091

**Published:** 2022-10-06

**Authors:** Clifford W. Sandlin, Song Gu, Jun Xu, Charuhas Deshpande, Michael D. Feldman, Matthew C. Good

**Affiliations:** 1 Department of Cell and Developmental Biology, University of Pennsylvania, Philadelphia, Pennsylvania, United States of America; 2 Department of Pathology and Laboratory Medicine, University of Pennsylvania, Philadelphia, Pennsylvania, United States of America; 3 Department of Bioengineering, University of Pennsylvania, Philadelphia, Pennsylvania, United States of America; 4 Nanjing University of Information Science and Technology, Nanjing, China; University of Melbourne, AUSTRALIA

## Abstract

Human cells tightly control their dimensions, but in some cancers, normal cell size control is lost. In this study we measure cell volumes of epithelial cells from human lung adenocarcinoma progression in situ. By leveraging artificial intelligence (AI), we reconstruct tumor cell shapes in three dimensions (3D) and find airway type 2 cells display up to 10-fold increases in volume. Surprisingly, cell size increase is not caused by altered ploidy, and up to 80% of near-euploid tumor cells show abnormal sizes. Size dysregulation is not explained by cell swelling or senescence because cells maintain cytoplasmic density and proper organelle size scaling, but is correlated with changes in tissue organization and loss of a novel network of processes that appear to connect alveolar type 2 cells. To validate size dysregulation in near-euploid cells, we sorted cells from tumor single-cell suspensions on the basis of size. Our study provides data of unprecedented detail for cell volume dysregulation in a human cancer. Broadly, loss of size control may be a common feature of lung adenocarcinomas in humans and mice that is relevant to disease and identification of these cells provides a useful model for investigating cell size control and consequences of cell size dysregulation.

## Introduction

Many human cell types grown in culture display strict homeostasis for cell size [1]. Studies in model systems show that cells coordinate cell growth and division during the cell cycle to achieve proper size control [2–6] and correct size variation at a population level [7, 8]. Although there has been progress to uncover factors that modulate cell size in mammalian culture [7, 9, 10], it is not clear which pathways determine the cell size set point for most proliferating cells. An added hurdle is that perturbations to the volumes of cultured cells normally cause reduction in cell growth rates and a loss of fitness [11]. As a result, it has been challenging to ascertain the role of cell size regulation in tissue physiology and to characterize the consequences of altered cell size on function. In short, new models are required [12]. To date, there are no mammalian model cell systems that enable broadly tunable control of volume without transiently swelling cells in which to study the biological impacts of altered cell size.

In certain diseased cell states, such as cancer and diabetes, cells display loss of size regulation [1, 2]. Analysis of proliferative cells of various types of cancer suggest that they lose normal control of cell size, nucleus size, or nucleus:cytoplasmic volume ratio [13, 14]. Abnormal nucleocytoplasmic ratios are often indicative of cancerous transformation [12, 15–19]. Additionally, alterations in dimensions of cell or nucleus have been tied to epithelial-mesenchyme transition (EMT) and invasion [20], and stemness [14], and thus there is significant interest in how loss of cell size control may contribute to tumorigenesis. More generally, in non-cancer model systems, alterations in cell size are linked to changes in gene expression [21, 22].

Although the molecular pathways that regulate human cell sizes are poorly understood, a number of general hypotheses have been put forth to explain increases in nucleus and cell volume or nucleocytoplasmic ratio found in some cancers. As early as 1930, it was Levine and his contemporaries who linked what he referred to as “semi-giant” cells to the tetraploid karyotype, and suggested “giant” cells had very high ploidies [23]. More recent refinements to this concept that ploidy drives size dysregulation are that alterations in nucleus size or cell size relate to genomic instability of cancer cells and subsequent broad increases of ploidy and cytoplasmic output [24, 25]. Studies in culture systems have also linked increased DNA content to increased cell size [26, 27]. Separately, because it has been observed that cells may transiently enlarge through increases in water content, including during mitosis [28, 29], osmotic swelling is commonly hypothesized to explain cell volume dysregulation. Indeed, measurements of sizes of the diverse cell lines that comprise the NCI-60 panel [30] show cell volume increases generally increase with ploidy, although some interpret aneuploidy to be the driving factor [30, 31]. Further, cell swelling has been observed in senescent cells and is accompanied by a reduction in cytoplasmic protein content [32], thus it might be assumed that enlarged cells do not contribute to disease. We wondered whether cells might lose regulation of their cell size set point, and do so in the absence of the increased ploidy or cell swelling, while retaining or even increasing proliferative functions. If so, these cells might provide generalizable insights on causes and consequences of size dysregulation.

Analyses of cell size dysregulation in human cancers have relied on proxy model systems, or indirect or bulk measurements that do not address ploidy on a per-cell basis. Measurement of cell areas in two dimensions at moderate magnifications also limits the ability to detect alterations in nucleus and cell shape. 2D spherical approximations underestimate cell volume, because the equator is a small portion of a sphere; the more complex the shape, the less reliable the 2D approximation. Nandakumar et al. addressed 3D cell and nuclear volumes and DNA contents of preneoplastic esophageal and also breast cancer cells in suspension [24, 25]. In both cases, they observed greater volume and DNA content for cell lines originating from the more progressed disease state, suggesting a link between volume, ploidy, and cell size. Dolfi et al. have provided the most complete dataset relating cell volumes and ploidy by measuring the entire NCI-60 panel with a Nexcelom Cellometer^**™**^. Although groundbreaking in establishing that cell size dysregulation exists in tumor cells, these are bulk measurements that are not bona fide tumors. Thus, a major advance would be three-dimensional image reconstruction of tumors and object segmentation of specific cell types that is essential to accurately characterize alterations to the shapes and volumes of cells. Thus, in this study we sought to characterize the loss of homeostatic cell and organelle size control for a defined cell of origin in situ in 3D, within a human tumor.

By first analyzing digital histology databases [33, 34] and screening pathology H&E slides, we identified lung adenocarcinoma (LA) as a cancer type that likely contains a large variation of epithelial cell sizes. LA is the most prevalent form of non-small cell lung cancer (NSCLC) [35]. NSCLC is the number one cause of cancer deaths in the United States, and was expected to claim more than 130,000 lives in 2021 [36]. Morphological categorization remains the underlying foundation for the classification of subtypes of NSCLC, suggesting more high-resolution data may provide a benefit to the field of pathology [17, 18, 35, 37–41]. LA morphology is described by lepidic, papillary, acinar, micropapillary, and solid patterns of growth, and the disease has well-established mouse models [35, 42, 43]. Experiments in mice suggest LAs may arise from transformation of epithelial stem cells, called airway-type II (AT2) cells present in the lung alveoli [44]. These stem cells secrete surfactant proteins to prevent alveolar collapse and help to repair damaged lung tissue [43, 45–48]. In this study, we also discovered that these cells display a network of thin processes containing pro-surfactant protein. Within the clinic, two predominant genetic forms of LA, EGFR+ and KRAS+, are mutually exclusive [49]. Notably, data on DNA content for LA across all stages [18] suggested ploidy might only partially explain our preliminary observation of cell size dysregulation. Thus, LA seemed to be an ideal candidate for characterizing the loss of cell size regulation.

In this study, we set out to explore the extent, prevalence and staging of epithelial cell size dysregulation in situ via quantitative analysis of three-dimensional image reconstructions of LA tumor specimens. Additionally, we sought to determine the extent to which size dysregulation was explained by alteration of ploidy or osmotic swelling and to what extent cells of altered size proliferated and maintained homeostatic control of protein content and organelle size scaling. To do so, we characterized cell and organelle volumes and shapes of epithelial cells in situ within EGFR+ and KRAS+ types across three stages of tumor progression, and compare them to normal AT2 cells. Our results show vast enlargement of epithelial cell and nucleus volume are present in LA, irrespective of ploidy. The range of cell and nucleus volumes increases up to eight-fold and up to 80% of euploid cells display size dysregulation at specific stages. The mean size of proliferating epithelial cells in LA is greatly increased suggesting that these cells contribute to tumor progression. Further, these enlarged cells are not simply swollen and instead show homeostatic control of protein content and size scaling or organelles, including the nucleus, nucleolus and nuclear speckles, indicative of their function. Cell size variation is associated with tissue organization in these tumors and nuclear size variation was also present in a mouse model for the disease. Additionally, to validate the presence of cell size variation is a property of near-euploid cells, we developed a method to isolate and separate human epithelial cells from LAs on the basis of their cell sizes using flow cytometry. Our data suggest that broad dysregulation of cell size is a prevalent feature of LA and that these proliferating epithelial cells, which maintain macromolecular content and scale organelle sizes may be a useful model for investigating mechanisms of size control and the consequences of size variation.

## Results

### Human lung adenocarcinoma displays significant cell and nuclear volume dysregulation that can be measured in 3D by exploiting machine learning

**Fig 1:**
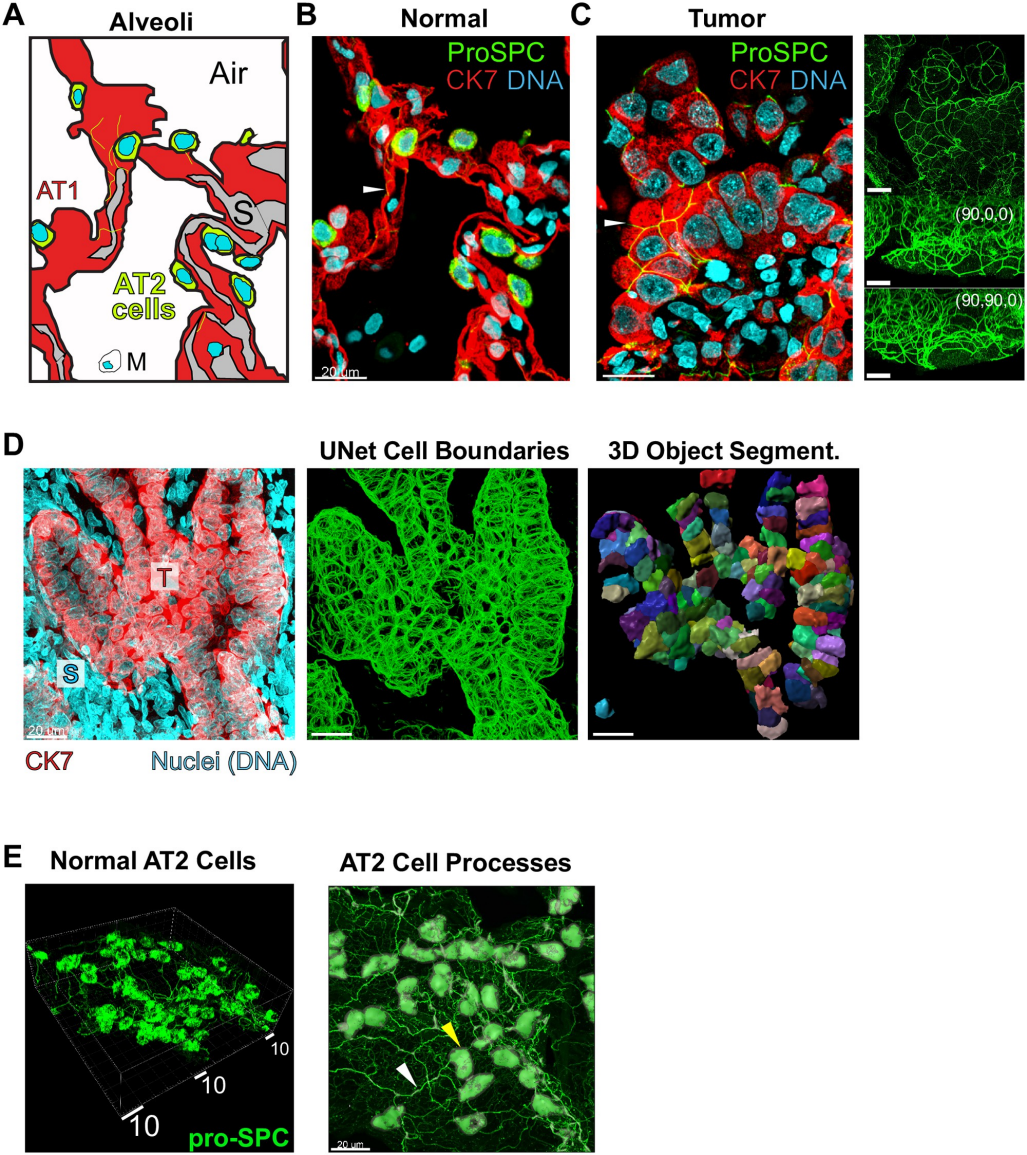
Human lung adenocarcinoma is characterized by dysregulation of cell and nuclear size. 63X images shown. A., schematized alveolus, related to B., showing AT2 cells (marker: proSPC, green), presumptive wide and flat AT1 cells, non-CK7-staining macrophage, M, and stroma, S. Epithelial cells express marker CK7, but weakly in AT2 cell bodies. B. and C., 5 μm z-projections of representative normal distant control alveoli, B., and representative EGFR+ LA tumor, AT2 cells marked by proSPC (green), C. Cytokeratin 7, CK7 (red); overexpressed in tumor cells. DNA stained by TO-PRO-3 (cyan). White arrows: thin cellular processes (shown in 1E, below). Right, full-stack max-intensity projections from each axis. Scale bars, 20 μm. Row D., 3D segmentation approach for tumor cells. Left, a high-density confocal stack often referenced during early development of our machine-learning algorithm. Middle, Machine Learning from CK7 staining predicts cell boundaries. Right, Commercial Imaris software fits cell boundaries, with rainbow coloring showing individually segmented cell surfaces. High-coverage case shown. E. Left, 3D rendering of AT2 cells (proSPC) showing thin cellular processes; right, processes shown with AT2 cell body segmentation (gray). White box shows perspective scale, 2 μm per small tick mark. Yellow arrow: AT2 cell body; white arrow: cellular process.

Human lung alveoli are structured air sacs with lumens which are surrounded by an epithelium. This epithelial layer is composed alveolar type 1 (AT1) and type 2 (AT2) cells (Fig 1A) and lies on top of a thin stromal layer. AT2 cells fulfill the critical functions of secreting surfactants that are released into the lamina of the alveolus from multilamellar bodies, and are the resident stem cells of the alveolus [42, 44, 45, 47, 48, 50, 51]. AT1 cells differentiate from dividing AT2 cells that undergo a process of shaping and flattening to a 1 μm thickness and loss of surfactant protein expression [45]. LA is believed to result from an oncogenic event in dividing AT2 cells in mice, although evidence for this in humans is limited [44]. LA cells are known to overexpress cytokeratin 7 before they undergo additional transformations [52].

We first asked whether it was possible to distinguish LA cells from their proposed AT2 progenitors in whole-mount tumor sections and to reconstruct three dimensional morphologies. In doing so we reasoned it would be possible to identify alterations in cell and organelle dimensions in situ in the context of local tissue organization. We performed whole-mount immunofluorescence (IF) confocal imaging of formaldehyde-fixed and paraffin-embedded (FFPE) [53] samples that comprised 150 μm-sections of pre-identified normal and LA patient specimens. FFPE is well-documented to undergo minor isotropic shrinkage over the length scale order of 1–100 μm relevant here, which depend on a given lab’s procedures [54]. However, it preserves cell and nuclear features and DNA content very well, while providing a route to deep image characterization of human samples that are already phenotyped, genotyped, and with well-defined treatment history [55]. To identify cell types in situ, we immunostained using antibodies specific to the unprocessed N-terminal region of prosurfactant protein C (proSPC), which is highly expressed in the cytoplasm of AT2 cells, and cytokeratin 7, which labels epithelial and LA cells with high sensitivity; it does not stain underlying stroma [42, 43, 48, 52, 56, 57]. To stain DNA for nucleus segmentation we used TO-PRO-3. For DNA content measurements we included stromal cell nuclei to establish normal baseline levels of DNA. At high magnification our image stack was typically ~135 x 135 x 60 μm [58].

Representative IF images of normal and LA specimens (Fig 1B and 1C) illustrate that delineation of AT2 and CK7-overexpressing tumor cells was straightforward and facilitated observation of novel cellular morphologies and changes to the cell size and shape. AT2 cells were easily identified as bright-green fluorescing cells (proSPC) mutually exclusive with the red channel in their cytoplasm (CK7), but bracketed by brightly red-fluorescing alveolar walls. These cells also have intense TO-PRO-3 DNA stains typical of cell nuclei (AT1 cells, Fig 1A shows a schematic). We observed AT2 cell bodies filled with dense green-fluorescing puncta, which mark multilamellar vesicles containing proSPC as expected for these secretory cells. Surprisingly, we also noticed that this protein marker extended into an interconnected network of thin “processes”, which to our knowledge have not previously been described (Fig 1B). In pre-identified tumor specimens, tumor cells were visibly obvious, presenting high intensity of red fluorescence in cytoplasm (CK7). Additionally, LA epithelial cells showed a dramatic enlargement of their cell bodies and nuclei (Fig 1C). LA cell nuclei are as large as cell bodies of normal AT2 cells and, at 63X magnification, display proSPC processes confined to the surface boundary of cell-cell junctions (Fig 1C, sidebar shows side-perspectives of channel 1). Tumor cell processes do not connect to cells classified as AT2, but in rare cases, tumor cell processes did connect directly to neighboring cells of an AT2-like morphology (S4B Video, punctate cells and S5 Video, yellow arrows). Concomitant with the enlargement of tumor nuclei, DNA organization often changes, becoming more spread-out and granular or cavitated appearance relative to DNA staining in stromal neighbors (Fig 1C). CK7 staining reveals cell boundaries of the epithelial cells and hints at a broadened range of cell volumes relative to normal AT2 cells (Fig 1C). Highly elongated stromal nuclei highly reminiscent of smooth muscle layers but of unknown type were also frequently observed (Fig 1D, “S”, and S4B Video, yellow circle).

To determine quantitatively whether these LA epithelial cells and their nuclei had enlarged we had to develop computational strategies for image segmentation. Due to challenges with immunostaining traditional cell boundary markers we ultimately decided to use CK7 stain for epithelial cell body segmentation. However the CK7 staining pattern precluded use of classical watershed approaches. To overcome this hurdle and automate cell body segmentation for hundreds of objects per stack, we trained a UNet-based convolutional neural network with a set of 40 optimized manual cell body annotations (S1 Fig). The resulting spaghetti model is a plot of the network’s confidence in a boundary (Fig 1D, middle, and S1 and S2 Figs), and represents 3D scatter data that can be fit by a surface model using Bitplane Imaris^™^ software (S3 Fig). Segmentation of AT2 cells from normal tissue was performed in parallel using straightforward thresholding approaches, with a comparable level of accuracy, as assessed by agreement with proSPC or CK7 data (S1D, S1E and S2–S4 Figs). Staining of AT2 cells also revealed thin cellular processes (Fig 1E), discussed later in the manuscript. Segmentation of nuclei was carried out using DNA stain and the cell surface models, correcting for variations in stack brightness (S5 Fig). Due to cell crowding in the tumor, we also manually corrected small clippings to construct an accurate nuclear model (S4 Fig). Notably, nuclear volume segmentation usually required filling cavities present in binary representations of nuclear volumes, highlighting that large regions of oversized tumor cell are DNA-poor (S5 Fig).

Initial 3D surfaces inevitably contained some inaccurate cell models and thus every cell and nucleus was subjected to thorough screening for qualitative agreement with CK7 and DNA data in both 2D and 3D (S3 Fig and Methods). The resulting quality-screened data are provided in S1 Data, tab B., and all quantitative data shown and discussed below are for cells passing this quality assessment screening. In general, the machine learning approach was able to recognize a cell boundary to a similar degree as the authors, and cells failing QC are indicative of poor CK7 staining. Thus, our dataset includes normal lung and LA lung epithelia that the human eye can clearly detect in the microscope and with clear boundaries that stain brightly with our IF protocol. An unfortunate limitation of our data is that it does not include small neoplasms that stain with some proSPC, but not with CK7. An overview of cell models with good cell coverage are shown in S1–S5 Videos; S4B and S5 Videos emphasize that small neoplasms and lesions stain poorly with CK7 and are thus not included in our data, but may be relevant to tumor cell development (green channels shown late in video and S5 Video, yellow arrows).

### Quantitation of cell and organelle dimensions in KRAS+ and EGFR+ lung tumors

**Fig 2:**
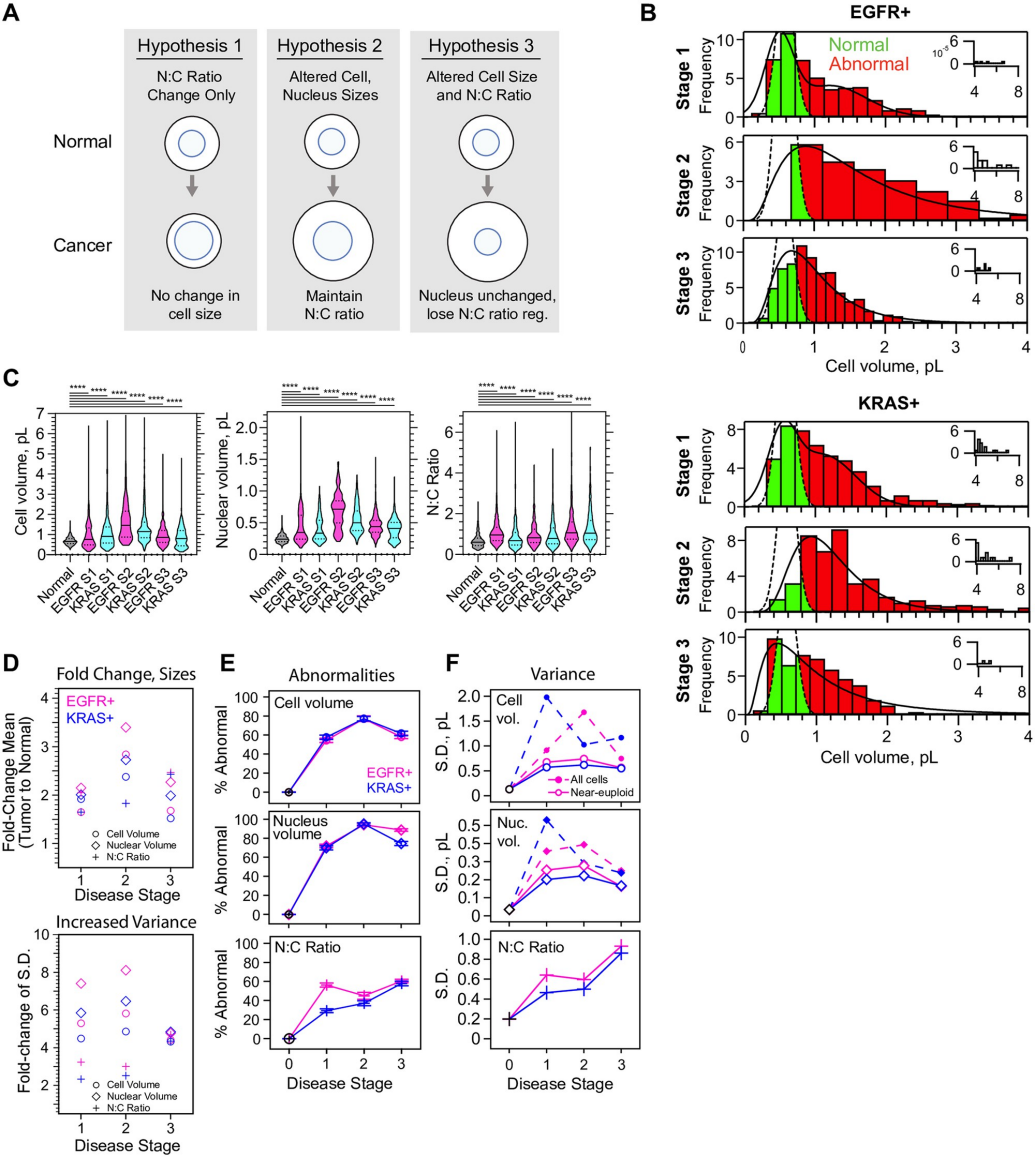
Quantitation of cell and nucleus dimensions in EGFR+ and KRAS+ lung tumors. A., Schematic: potential outcomes for cell and nucleus scaling in cancerous cells, hypothesis 2 is that LA retains nuclear scaling function. B., histograms of cell volumes of near-euploid (1.6–4.4n) cells (N = 21, n = 2683) as a function of EGFR+ (N = 10, n = 1411) or KRAS+ (N = 11, n = 1260) genotype and stage. Green, cell volumes for tumor cells within the boundaries of the 2n normal AT2 cells; Red, cell volumes outside the Gaussian distribution of 2n population of Normal AT2 cell volumes. C., violin plots for near-euploid cells show very significantly abnormal scaling and significant changes in distributions between stages. 1–2 and 2–3. Colors alternating for visibility, normal in grey. Kruskal-Wallis ANOVA, p<1 x 10^−4^ (****). Select pairwise p-values for Mann-Whitney tests in Tables 1–3. D.-E., quantitation of differences between near-euploid tumor cells and 2n wild-type AT2 cells. Circles, cell volume. Diamonds, nuclear volume. Crosses, N:C ratio. D., Corresponding fold change (tumor-to-normal) for cell sizes (top); standard deviation (bottom). Error bars ≤ 2.7 x 10^−3^ omitted for clarity Magenta, EGFR+, Blue, KRAS+. E., percentages of cells which fall within the regression fits to histograms for tumor cells, but outside the Gaussian fit to 2n AT2 cells; showing cell volume, nucleus volume and N:C ratio; colored by stage. Error bars, SE. Lines connect points to show trends. F., variance of cell size parameters as a function of stage. Dotted lines and solid markers, ploidy most significantly affects the variance of cell and nuclear volume. Related to Table 4 and S1 Data gives detail.

It is commonly observed that proliferating cells in tumors display a high nucleus:cytoplasm (N:C) volume ratio [13, 14]. However, observations of cell volume dysregulation are less frequently reported. Therefore, we wondered whether reconstructed LA cells, filtered for near-euploid DNA signal, would display only alterations of N:C ratio or whether they might show bona fide increases in cell and nucleus volume (Fig 2A). We found that for both genotypes of LA, epithelial cells had cell volumes and nucleus volume that drastically differed from normal AT2 cells (Kruskal-Wallis and ANOVA, p < 10^−4^, Fig 2B–2D, S6–S8 Figs). AT2 cell and nuclear volume data with overall mean ± SD of 682.3 ± 194.2 fit to a 2-Gaussian mixture model to recover a first peak of cell volume ± σ of 582 ± 127 fL (n = 802, χ^2^/df = 1.265 x 10^−8^) and nuclear volume of 202 ± 34.2 fL (n = 802, χ^2^/df = 9.285 x 10^−8^). In contrast epithelial cells across all stages of LAs have mean ± SD cell volumes of 1334 ± 1340 fL (n = 4133), and nuclear volumes of 553.3 ± 378.6 fL (n = 4082). Considering only near-euploid cells, defined as 1.6–4.4n (n = haploid genome), gives cell volumes of 1086 ± 755.6 fL (n = 2683) and nuclear volumes of 462.3 ± 222.2 fL (n = 2671, S6 and S7 Figs). However, inspection of the cell volume distributions and outliers in the data reveals that eliminating cells >4.4n results in elimination of outliers in the long tail >7 pL and loss of the long tail in the distribution (Fig 2B and S6–S8 Figs). Thus, in the absence of ploidies > 4.4n, size dysregulation, defined as standard deviation (S.D.), is 5.9-fold higher for cell volume and 6.5-fold higher for nuclear volume in diseased versus normal (Fig 2C and 2E). Surprisingly, alterations of N:C ratio were much more modest (Fig 2E and S8 Fig) compared to the extent of cell and nucleus size alteration (Fig 2C and 2D). Importantly, polyploidy contributes to size dysregulation by a 77.6% increase in the S.D. of tumor cell volume relative to near-euploid tumor cells (Fig 2F). However, we still observe an 86.6% increase in mean volume of near-euploid tumor cells relative to 2n AT2 wild-type cells. Thus, polyploidy is not necessary to observe a nearly 2-fold cell-size increase in human LA.

**Table 1:** P-value results of pairwise Mann-Whitney testing select conditions shown in Fig 2C, cell volume of near-euploids.

	Normal	EGFR S1	EGFR S2	EGFR S3	KRAS S1	KRAS S2	KRAS S3
Normal	NA	1 X 10^−4^	NM	NM	NM	NM	NM
EGFR S1	NA	NA	NM	NM	1.2 x 10^−3^	NM	NM
EGFR S2	NA	NA	NA	NM	1 x10^-4^	4 x 10^−4^	NM
EGFR S3	NA	NA	NA	NA	NM	1 x 10^−4^	1.5 x 10^−3^
KRAS S1	NA	NA	NA	NA	NA	NM	NM
KRAS S2	NA	NA	NA	NA	NA	NA	NM
KRAS S3	NA	NA	NA	NA	NA	NA	NA

**Table 2:** P-value results of pairwise Mann-Whitney testing select conditions for nuclear volume of near-euploids.

	Normal	EGFR S1	EGFR S2	EGFR S3	KRAS S1	KRAS S2	KRAS S3
Normal	NA	1 X 10^−4^	NM	NM	NM	NM	NM
EGFR S1	NA	NA	NM	NM	0.2410	NM	NM
EGFR S2	NA	NA	NA	NM	1 x10^-4^	1 x 10^−4^	NM
EGFR S3	NA	NA	NA	NA	NM	1 x 10^−4^	1 x 10^−4^
KRAS S1	NA	NA	NA	NA	NA	NM	NM
KRAS S2	NA	NA	NA	NA	NA	NA	NM
KRAS S3	NA	NA	NA	NA	NA	NA	NA

**Table 3:** P-value results of pairwise Mann-Whitney testing select conditions for N:C ratios of near-euploids.

	Normal	EGFR S1	EGFR S2	EGFR S3	KRAS S1	KRAS S2	KRAS S3
Normal	NA	1 X 10^−4^	NM	NM	NM	NM	NM
EGFR S1	NA	NA	NM	NM	1 x10^-4^	NM	NM
EGFR S2	NA	NA	NA	NM	2 x10^-4^	0.3321	NM
EGFR S3	NA	NA	NA	NA	NM	1 x 10^−4^	0.7944
KRAS S1	NA	NA	NA	NA	NA	NM	NM
KRAS S2	NA	NA	NA	NA	NA	NA	NM
KRAS S3	NA	NA	NA	NA	NA	NA	NA

**Table 4:** Percentages of abnormal cell volumes, nuclear volumes, and N:C ratios vs. stage, genotype, and DNA content.

Genotype	Stage	Ploidy range	Cell volume	Nuclear volume	N:C ratio	Number of Patients, N	Number of cell models, n (% total)*
EGFR+	1	All cells	55.9 ± 1.7	76.4 ± 1.4	56.7 ± 1.7	4	876
EGFR+	1	Near-euploids	54.5 ± 2.1	72.0 ± 1.9	56.3 ± 2.1	4	555 (63.4%)
EGFR+	2	All cells	89.8 ± 1.3	97.1 ± 0.7	47.0 ± 2.2	3	514
EGFR+	2	Near-euploids	77.2 ± 3.0	94.2 ± 1.7	45.0 ± 3.6	3	190 (37.0%)
EGFR+	3	All cells	59.0 ± 1.7	88.2 ± 1.1	61.1 ± 1.7	3	805
EGFR+	3	Near-euploids	58.0 ± 1.9	88.7 ± 1.2	60.4 ± 1.9	3	666 (82.7%)
KRAS+	1	All cells	65.8 ± 1.8	71.1 ± 1.7	26.1 ± 1.6	4	734
KRAS+	1	Near-euploids	57.7 ± 2.2	70.0 ± 2.1	29.3 ± 2.0	4	498 (67.8%)
KRAS+	2	All cells	82.4 ± 1.6	95.0 ± 0.9	35.4 ± 2.0	4	572
KRAS+	2	Near-euploids	77.2 ± 2.3	95.0 ± 1.2	37.1 ± 2.6	4	340 (59.4%)
KRAS+	3	All cells	63.0 ± 1.9	79.7 ± 1.6	55.8 ± 2.0	3	633
KRAS+	3	Near-euploids	61.8 ± 2.4	74.5 ± 2.1	57.8 ± 2.4	3	422 (66.7%)

*See also, S1 Data. Standard error of percentages are estimated as the standard error of a proportion. See methods.

Next we asked whether the level of size dysregulation of near-euploid LA cells varied with cancer genotype or disease progression. Therefore we included in our dataset 10 patients pre-identified with either EGFR+ and 11 with KRAS+ genotypes, with 3–4 patients staged 1–3. Collapsing all stages, there is little difference between cell or nuclear volume distributions for EGFR+ and KRAS+ patients (S6 and S7 Figs). However, separating data by stage and genotype reveals subtle differences in the prevalence and trajectories of size dysregulation (Fig 2B and 2C, S9 and S10 Figs). Although we expected cell size dysregulation would worsen with stage, the independent EGFR+ (n_near-euploid_ = 1411, n_total_ = 2194) and KRAS+ (n_near-euploid_ = 1260, n_total_ = 1939) datasets both displayed the highest fraction of cell and nucleus size dysregulation at stage 2 (Fig 2C–2E, S9 and S10 Figs). Somewhat convincingly, both genotypes track together in terms of their trajectories of size dysregulation with stage (Fig 2B–2E). A significant difference between two genotypes occur at stage 1, in which KRAS+ tumor cells displayed more normal nucleocytoplasmic ratios (Fig 2C and 2D), perhaps due to slightly larger cell size and smaller nuclei compared to EGFR+ cells (Fig 2D).

Importantly, size dysregulation is not caused by a small subpopulation of cells in these LAs. Considering near-euploid cancer cells, we calculated the percent of cells whose cell volumes and nucleus volumes extended outside of the first normal AT2 Gaussian (Fig 2E, Table 4 and Methods). We found that 54.5–77.2% of near-euploid cells fall outside the range of normal AT2 cells, suggesting that size dysregulation is highly prevalent in both tumor genotypes. The extent of dysregulation is most dramatic stage 2, in which 95% of all nuclear volumes are abnormal and more than 77% of cell volumes, irrespective of ploidy (Fig 2D, Table 4). The percentage of cells with abnormal cell or nucleus sizes is also largely independent of cell ploidy (Table 4). N:C ratio abnormalities are more modest for near-euploids, although they do increase by stage 3 of LA progression (Fig 2C and 2D, S11 Fig). In EGFR+ the N:C ratio dysregulation occurs earlier than in KRAS+, reaching a plateau at stage 1 (Fig 2D). The increase in N:C ratio observed at stage 3 appears consistent with slight reductions in cell volume but considered across all cells some scaling is evident (S11 Fig). Altogether our data support partial nuclear scaling with cell volume in cancer, and broader general shifts upward in terms of nucleus size and cell size relative to the wild type.

### Cell and nucleus size dysregulation is not simply explained by altered ploidy, and the extent of dysregulation differs between patients

**Fig 3:**
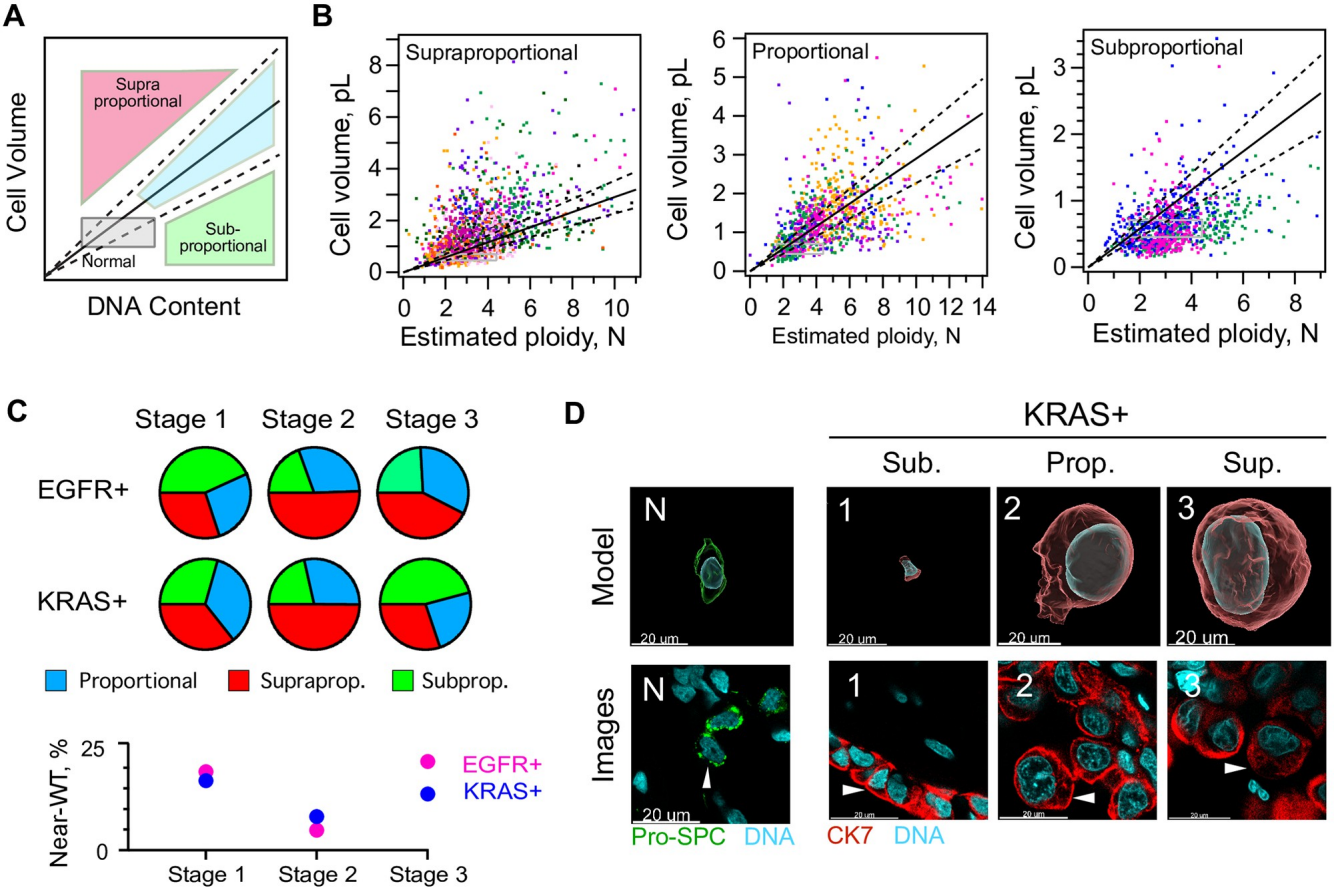
Ploidy alone cannot explain a majority of cell and nuclear size dysregulation. 63X images shown. A., schematic: subgroups of scaling relationships between DNA content and cell volume: supra: cell volumes larger than expected for ploidy; sub: DNA content higher than expected for cell volume. Colors, individual patient data. Grey box, near-WT, with AT2 volume μ ± σ and near-euploid (n = 1.6–4.4). B., distributions of cell ploidy and volume for individual cells; colored to distinguigh patients. Solid black line indicates simple multiplication of the estimated cell volume and ploidy of a normal AT2 cell, or the equivalent slope; showing +/- 1 S.D., dotted lines. For example, the solid passes through the point (2 x 582 fL, 2 x 2n), where 582 is the mode of the first gaussian fit to the AT2 cell volume distribution (See S6 Fig). The three plots each represent a binning of patients from all stages and both genotypes, but with either no preference, Proportional, or a departure from this line, Subproportional, or Supraproportional. C., top, the fraction of cells classified within each category of abnormal (EGFR+, N = 10, n = 2195, KRAS+, N = 11, n = 1939, S1 Data, tab E). More cells in stage 2 display the supraproportional phenotype, and subproportional cells are enriched in stage 3 KRAS+ cancer. C., bottom, Near-WT, cells with cell volumes close to WT (455–709 fL) that are also near-euploid (1.6–4.4n) are rare in LA, but are depleted in stage 2. Magenta, EGFR+, Blue, KRAS +. D., images of high-quality KRAS+ cell models for each representative category, right, with corresponding 2D images; N, typical AT2 controls. 1, subproportional, 2 proportional, 3 subproportional. White arrows, cells corresponding to models seen in 3D renderings. Scale bar, 20 μm.

Polyploidy is known to have a general effect on cell volume, and is a feature of aggressive cancers that is proposed to affect drug resistance and recurrence [18, 59–62]. Therefore, we asked to what extent cell and nucleus size dysregulation could be explained by changes in genome content, and whether individual patients showed distinct scaling relationships. For this analysis we plotted the volume of each tumor cell against its ploidy for each patient specimen (S12 and S13 Figs), compared to a prediction line that represents a simple multiplication of the mean DNA and volume of a normal AT2 cell. Patient samples that showed a significant number of abnormal cells above the AT2 prediction for polyploidization were classified as “supraproportional”, meaning higher cell volume enlargement than expected for the ploidy (Fig 3A and 3B, S12 and S13 Figs, see Supplemental Methods for our decision rule). Patient samples containing many cells below the line were classified as “subproportional”, or having smaller cell volumes than expected for polyploid. A majority(52.4%) of patient samples showed nonrandom enrichment of, and a majority of supraproportional cells (Fig 3B and 3C, S1 Data, tab E). Supraproportional cell volume behavior is highest at stage 2 for both genotypes (50.6% of EGFR+ cells, 49.9% of KRAS+ cells, Fig 3C, pie charts) consistent with their being the highest amount of size dysregulation (Fig 2B and 2C). By stage 3 there is increased prevalence of both “proportional” cells–cells with cell volume:ploidy that acts like simple multiplication of AT2 cell volume:ploidy—and “Near-WT” cells, near-euploids classified with cell volume within μ_WT_ ± σ = 582 ± 127 ratio (Fig 3A, grey box and Fig 3C, bottom). KRAS+ cells become more subproportional in stage 3, suggesting elevated ploidy without proportionate cell size increases (Fig 3B, top). Examples of supra- and subproportionality are visible in IF (Fig 3D). From these observations we conclude that size dysregulation is common in patients, and that its dependence on ploidy is patient-specific and not predominant.

### Cell-size dysregulation is strongly correlated with loss of tissue organization and local tumor cell density

**Fig 4:**
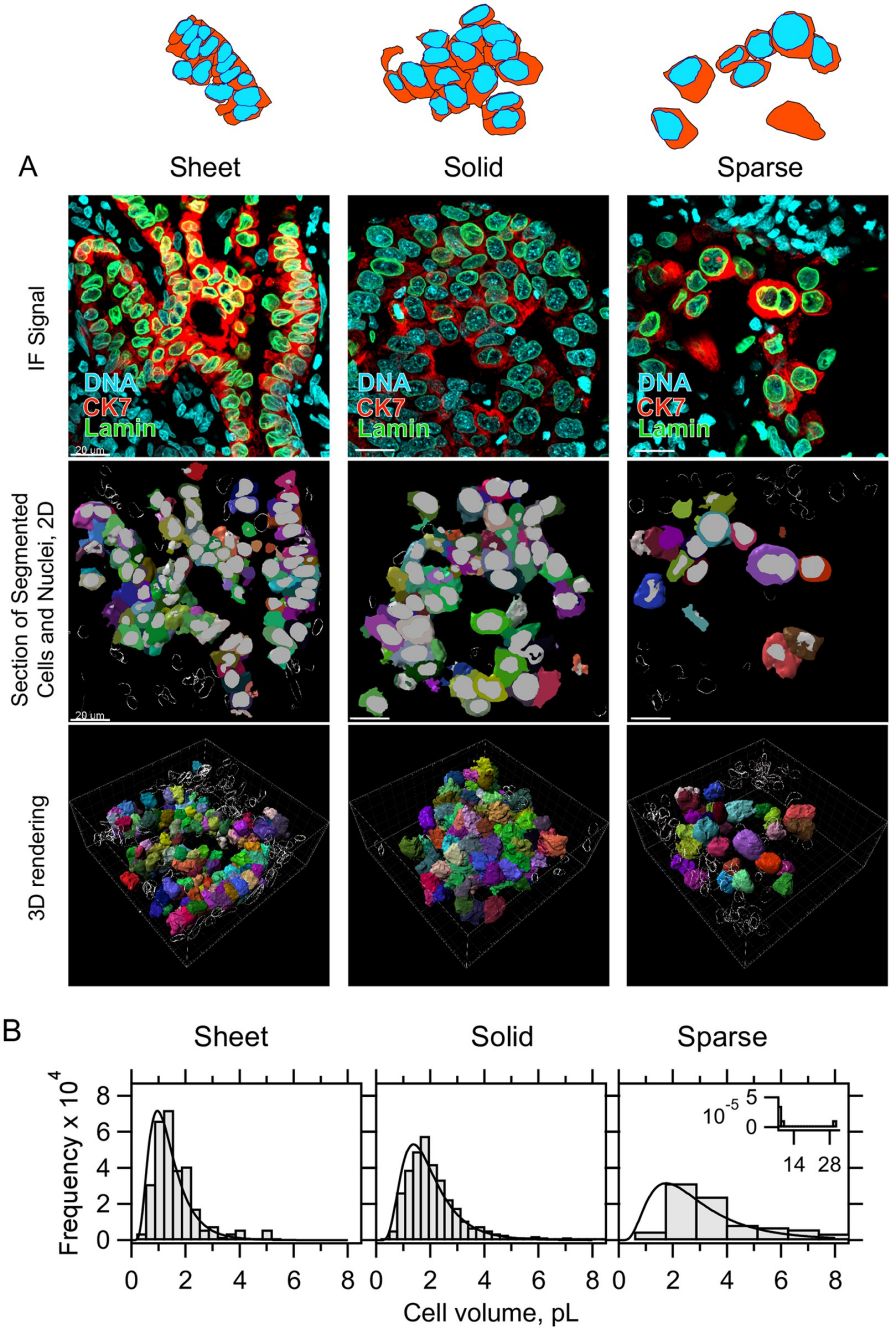
Tumor tissue organization correlates to the extent of cell size dysregulation. A, Schematic: sheet, solid, and sparse patterns of tissue organization describe most lung tumor data. Top: representative IF signal for three types, immunostained for CK7 (red) and Lamin A+C (green), observed in both EGFR+ and KRAS+ genotypes, EGFR+ patients shown. middle: Renderings of 2D segmentations; nuclei (gray) and cell surfaces (rainbow), Scale bars, 20 μm. bottom: rendering of 3D segmentations, with stromal controls shown as bubble depictions. White box shows perspective scale, 2 μm per small tick mark. B., cell volume distributions taken from segmented image stacks similar in morphology to the canonical ones shown, exclusively from stage 2 LA and from both genotypes. Sample sizes N patients and n cells, parentheses show respective (EGFR, KRAS) samples: N_Sheet_ = 4 (1,3), n_sheet_ = 155 (63,92), N_Solid_ = 4 (2,2), n_Solid_ = 438 (275,163), N_Sparse_ = 2 (1,1), n_Sparse_ = 73 (22,51).

We wondered whether tissue organization within LAs might relate to the phenotype of cell size dysregulation. Gut-like morphologies–sheets of epithelial cells–are not normally found in lung alveoli, but are commonly seen in LAs [42]. Within our three-dimensional image reconstructions of LAs we identified three general categories of tumor tissue organization (Fig 4). A gut-like sheet pattern of cells we refer to “sheet growth” and is present in 61.0% of our image stacks (45/75, Fig 4, left, S1, S4 and S5 Videos, and S1 Data, tab H.). A second pattern of more “solid growth” contained highly packed cells that took on either a cuboidal appearance or one of spheroid tangled cells with elongated tails (46.7%, 35/75 image stacks, Fig 4, middle, and S2 Video). A third pattern of “sparse growth” often neighbored the other two patterns and tended to contain a lower density of cells (8.0%, 6/75 image stacks, Fig 4, right, and S3 Video). Quantification of image stacks from both genotypes predominantly containing each growth type at stage 2, shows that the extent of cell volume dysregulation correlates with tissue pattern type (Fig 4B). Both the mean and breadth of cell volume distributions tracks with tissue organization in the tumor (μ_Sheet_ = 1430 ± 850, μ_Solid_ = 1963 ± 1083, μ_Sparse_ = 3439 ± 3480, errors SD, Fig 4B and S1 Data tab G.). Cells with sparse organization tend to achieve largest sizes. Additionally, smaller cells tend to be more elongated, consistent with patterns of sheet like growth (S15 Fig). These data suggest that cell size dysregulation may be linked to loss of local tumor organization and cell density.

### Human lung tumor cells of near-euploid DNA content can be purified on the basis of cell size

To confirm cell size dysregulation occurs in near-euploid LA and rule out artefacts related to imaging or sample preparation, we decided to isolate cells from resected tumors, select for cells of lower, near-euploid DNA amounts, and sort them by size using flow cytometry. Building on a general framework for separating human cells correlating their dimensions to light scattering profiles [63], we developed a FACS-based strategy for separating near-euploid human LA epithelial cells on the basis of their sizes.

This approach combines three protocols developed previously: a gentle digestion cocktail to preserve CD45 receptors for removing blood cells [64, 65], fixing cells to preserve their shape in the flow cytometer, and quenching fixative with glycine to prevent cell clustering [66]. Our cell suspensions were CD45- after depleting immune cells. Importantly we determined that for epithelial cells from LAs, side-scattering width (SSC-W) parameter was proportional to cell cross-sectional area (A_Cell_ = (-159 ± 44) + (4.35 ± 0.48)SSC-W μm^2^, N = 12, S16 Fig) [63].

**Fig 5:**
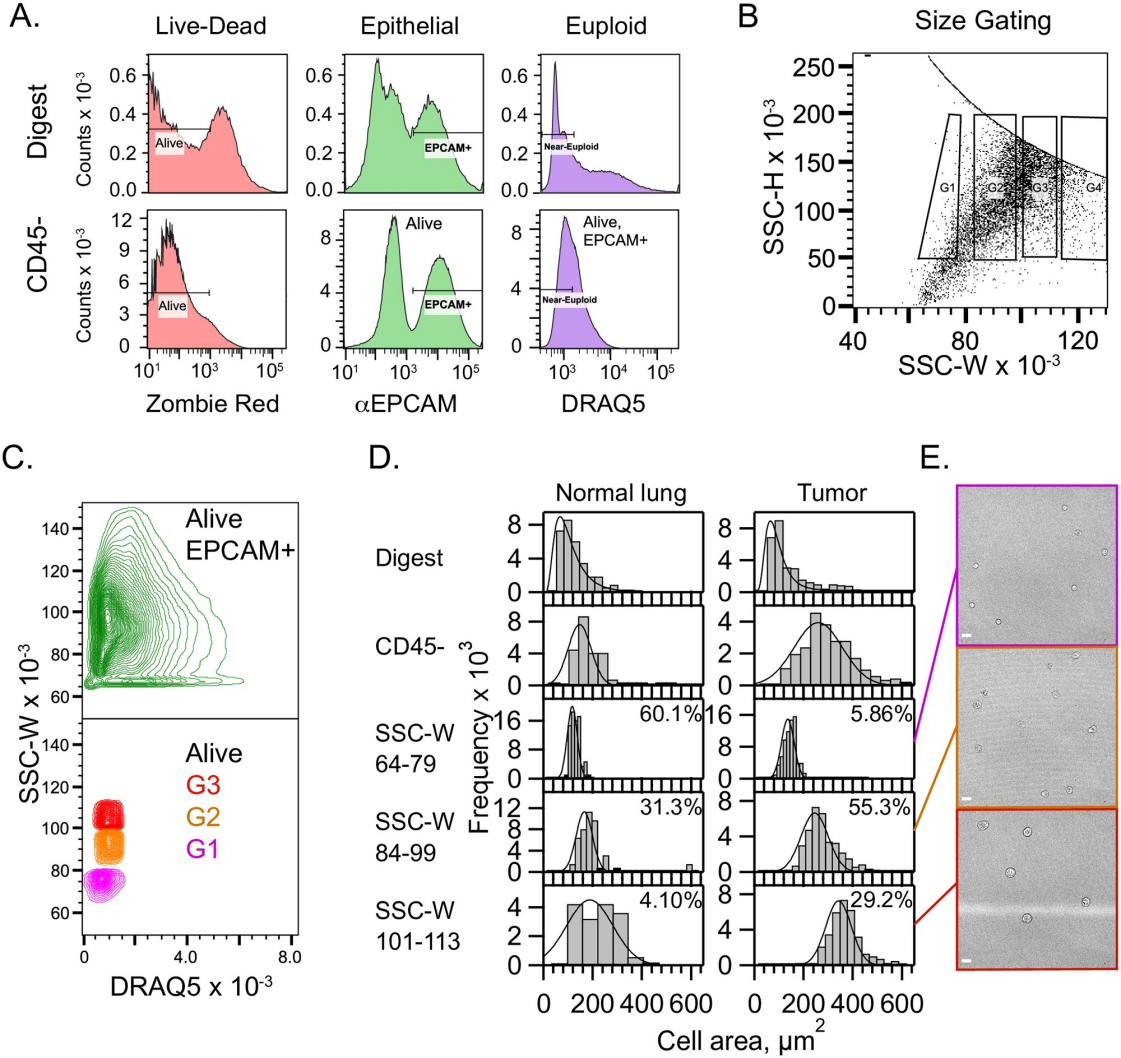
Near-euploid tumor cells can be purified and sorted by cell size using flow cytometry. A. 9.43 x 10^5^ total events shown for a dissociated tumor from patient 5296 of unspecified genotype and grade. Signal gates to purify epithelial cells that were alive when fixed, and with near-euploid DNA amounts are shown left-to-right overlaid on fluorescence histograms for the parent digest, top row, and CD45- MACS flow-through, bottom row. The DNA gate is 1.5x the mode shown for CD45-. B., The SSC-H vs SSC-W plot is gated as shown to separate Zombie Red-/EPCAM+/DNA- cells selected from A. C., Contour plot of the SSC-W as a function of DNA for Zombie Red-/EPCAM+ cells, green, as compared with DNA-size-sorted cells as shown. Size-sorted tumor cells have SSC-W that is essentially independent of DNA amount. D., Representative histograms of cell areas measured from brighfield images of cells on a hemacytometer as shown in E., for normal lung tissue, left, and a LA tumor, right. Top to bottom, each row shows the parent, the MACS CD45- flow-through, and the results from the three size gates. Percentages, relative cell yields for gates G1-G4. G4 always contained low yield aggregates. E., brightfield images of cells sorted by gates G1-G3, and with mean cell areas close to those shown in 5D. Scale bar, 20 μm.

To enrich cells of interest we gated for live and dead cells using Zombie Red, epithelial cells using EPCAM, and lower ploidies using the low-DNA DRAQ5- gate (<1.5x mode, Fig 5A). We further separated cells into size bins using SSC-H vs. SSC-W gates as shown (Fig 5B, SSC-W = 64–79, 84–99, 101–113). Reassuringly, cells were in clearly distinct bins of SSC-W but had normal DRAQ5 content (Fig 5C). After sorting, the bins showed clear separation by cell area (Fig 5D). As expected, normal lung tissue displays only modest shifts in size distribution and a small yield of cells in the largest cell-size gate (Fig 5D and S17 Fig). SSC-A vs FSC-A plots show that FSC-A does trend upward with SSC-W gates G1-G3 as expected for objects of increasing cross-section (S18 Fig shows a traditional). These results are consistent with those from whole mount tumor imaging, and further demonstrate the high prevalence of epithelial cell size dysregulation in LA, independent of high ploidy.

### Cell-size dysregulation is AT2-like, and correlated with loss of a novel network of cellular processes

**Fig 6:**
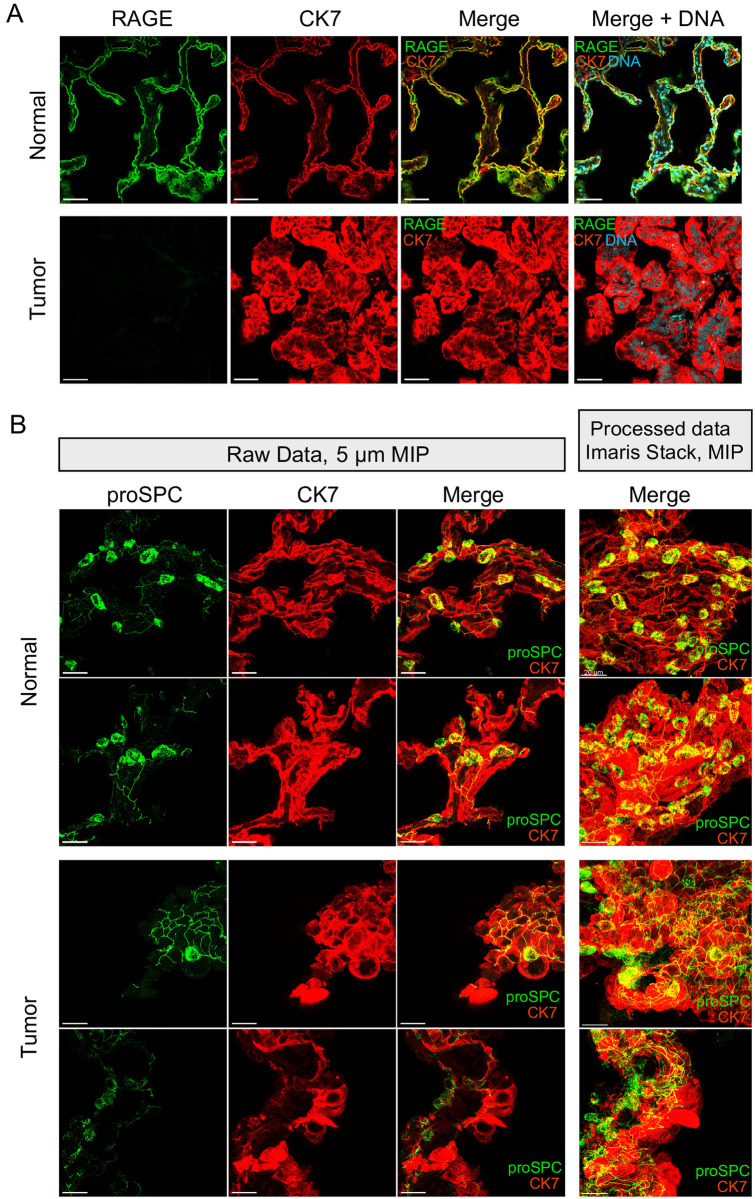
AT2-like cells display cellular processes whose organization is altered in lung adenocarcinoma. A., 25X images at 0.3779 μm resolution showing a single plane display RAGE, (green) is expressed highly in AT1 cells in normal tissue, top, but we could not detect expression in either genotype of tumor cells bottom. KRAS+ shown. Cells immunostained for CK7 (red) and DNA (cyan). B., 63X images shown. Left three columns, raw data shown in a maximum-intensity projection (MIP) 5 μm thick illustrates the behavior of the AT2 process network in normal distant controls, top, and tumor lung, bottom. Right column, a snap from the Imaris workstation of a MIP over an entire processed image stack shows the overall structure of the process network. Each row shows a different specimen (see Table 5). proSPC (green), CK7 (red). Bottom row, transition regions are observed in both genotypes of LA and contain presumptive diseased AT2-like cells that neighbor enlarged CK7-expressing cells. EGFR+ tumors shown (also S4 and S5 Videos).

In another attempt to explain cell size dysregulation in LA, and given the observation that sheet-growth cells are smaller and retain size regulation, we considered whether cell size dysregulation in is in any way linked with preexisting pathways for differentiation. AT2 cells differentiate into AT1 cells through a process of flattening coupled to a loss of expression of the AT2-specific marker prosurfactant protein C (proSPC, [45]). As AT1 volumes are unknown, we considered whether cell size dysregulation results from AT1-like differentiation, sans flattening. However, RAGE, a marker specific for AT1 cells in normal tissue showed no signal in LA cells (Fig 6A). In addition, we observed significant flattening of LA cells only rarely (S19 Fig). Thus, our data are not consistent with a model of AT1-like differentiation leading to cell size dysregulation in LA.

**Table 5:** Patient identifiers corresponding to data used to create each image shown and select figures.

Figure	Patient ID(s)
1A and 1B	4850
1C	4839
1D	4843
1E	4852
2,3B and 3C	See Table 7
3D, left to right	4843, stack N3, cell 18; 4856, stack T3, cell 34; 4848, stack T2, cell 882; 4850, stack T3, cell 82,
4A (left, middle, right)	4843, 4840, 3437
4C	Sheet: 4842T1,4842T3, 4851T2, 4852T4,4853N4
	Solid: 4840T1, 4840T2, 4842T2, 4842T5, 4850T4, 4850T5, 4853T3.
	Sparse: 4842T4 4850T3
5	5283,5296,5301 (5296 shown)*
6A	4852, 4839 RAGE also imaged but not shown
6B (top to bottom)	4841, 4852, 4837, 4843.
7B	4848, 4855 (4848 shown)
7D	4855
7F	4855
7H	Normal, 4852, Voids, 4843
	Lamin-associated DNA, 4841, Granular, 4840, wagonwheel, 3437, cytoplasmic inclusions, 4855
S1	4843
S2	4843
S3	4840
S4	4854
S5 (left to right)	4846, 4840, 4838
S14A	4854
S16	5283,5296,5301,5230,5231,5338,5353,5356,5365,5367,5376,5428
S17 (reading order, also labeled)	5230, 5231, 5283, 5301, 5296*
S18	5296*
S19	4854
S20	4855, 5531 (the mouse ID)
S21A (by column, left-right)	4841, 4841, 4837, 4843
S21B	4854
S22 (reading order)	4839, 4837, 4848, 4849, 4854, 4839
S23, top to bottom (also labelled)	4846, 4855, 4846
S24 (N = 8 of each genotype)	4837, 4838, 4840, 4841, 4842, 4843, 4844,
	4845, 4846, 4847, 4851, 4852, 4853, 4854, 4855, 4856
S30	4844
S1 Video	4843
S2 Video	4840
S3 Video	3437
S4 Video	4843
S5 Video	4855
S6 Video	1484*
S7 Video	1484*
S8 Video	1484*
S9 Video	1484*
S10 Video	1484*
S11 Video	1484*
S12 Video	1484*
S13 Video	1484*

*ID#’s not included in Table 7 were of unspecified genotype or grade.

Next we considered the alternative possibility that LA cells express AT2-specific markers and show morphological changes characteristic of AT2 cells, as suggested by results in mice [44]. Fortuitously, IF revealed a previously unreported feature of human AT2 cell anatomy, along with a corresponding pathology in LA cells (Fig 6B). We refer to these structures as “processes” or “process networks”. Processes are robust to antibody titration and display incomplete colocalization with E-cadherin and CK7 (S20 and S21 Figs). We supposed that if processes are a cross-reaction with extracellular matrix (ECM), they should disappear during a mechanically gentle collagenase/dispase digestion over several hours. However, observations revealed they are clearly not elements of ECM (S6–S12 Videos). During digestion, processes remain wrapped in shafts of intracellular cytokeratin (S11 Video), can remain connected between AT2 neighbors (S8 and S9 Videos), and can even be freed from ECM entirely (S7 and S12 Videos). Although exceedingly fragile, processes loosely follow the paths of CK7 bundles and show a level of complexity not typical of cross-reaction artefacts (S21 Fig, S4A and S4B Video). Amino acids 1–100 of proSPC that were the antigen for our antibody do overlap with the 35-residue region that makes up only 10% of the mass of the secreted form, yet we observe no significant reaction with the inner alveolar walls [48]. We hypothesize that processes are components of the cytoplasm of human AT2 cells. We do caution that a second antibody frequently used in mouse studies, raised against residues 1–32 of human proSPC was less effective in our hands and was not able to detect processes like those shown (S20 Fig and Table 6). Neither antibody we tested detected processes in normal or LA mouse samples. Thus, if mice possess this trait, an antibody raised against the mouse propeptide 1–100, with identity of 86% with the human sequence, may be required to reveal these structures (S20 Fig). In human LA, process-like staining is visible in sheet and solid growth patterns (Figs 1C and 6B, S22 Fig). Sparse CK7-positive cells are usually visible in proximity to sheet and solid regions, where they lack process-like staining (S22 Fig). Conversely, collagenase-resistant clusters of LA cells retain process-like staining at their interior junctions (S13 Video). These results support a link between cell size dysregulation and alterations in surfactant physiology, and strongly suggest human LA cells are AT2-like.

**Table 6:** Primary and secondary antibodies used in this study.

Antigen	Host	Clonality/Conjugation	Manufacturer	Catalog number or Name	Application/Fold dilution
Human Pulmonary surfactant-associated protein C, propeptide 1–100 (proSPC)	Rabbit	Polyclonal/-	Abcam	ab90716	1° IF / 1:200
Human Pulmonary surfactant-associated protein C, propeptide 1–32 (proSPC)	Rabbit	Polyclonal/-	Millipore Sigma	AB3786	1° IF / 1:200
Proprietrary synthetic lamin A/C propeptide (Lamin A+C)	Rabbit	Monoclonal/-	Abcam	ab108595	1° IF / 1:200
Proprietary synthetic Fibrillarin peptide (Fibrillarin)	Rabbit	Polyclonal/-	Abcam	ab5821	1° IF / 1:200
Human SON/DBP5 1463–1598 (SON)	Rabbit	Polyclonal/-	Abcam	ab121759	1° IF / 1:200
Keratin, type II cytoskeletal 7 (Cytokeratin 7, CK7)	Mouse	Monoclonal/-	Novus Bio	OV-TL 12/30	1° IF / 1:100
Rabbit Gamma Immunogobulin Heavy and Light Chains (IgG)	Goat	Polyclonal/AlexaFluor-488	Thermo Fisher	A-11034	2° IF / 1:500
Mouse Gamma Immunogobulins Heavy and Light Chains (IgG)	Goat	Polyclonal/AlexaFluor 568	Thermo Fisher	A-11004	2° IF / 1:500
Human Epithelial cell adhesion molecule (EPCAM)	Mouse	Monoclonal/AlexaFluor-488	BioLegend	324210	1° Flow / 1:50
Proliferation marker protein Ki-67 (Ki-67)	Rabbit	Polyclonal/-	Abcam	Ab15580	1° IF / 1:500
Advanced glycosylation end product-specific receptor 39–58 (RAGE)	Rabbit	Monoclonal/-	Abcam	ab37647	1° IF / 1:200

### Significance of dysregulated cell and nucleus sizes to cell and tumor biology

**Fig 7:**
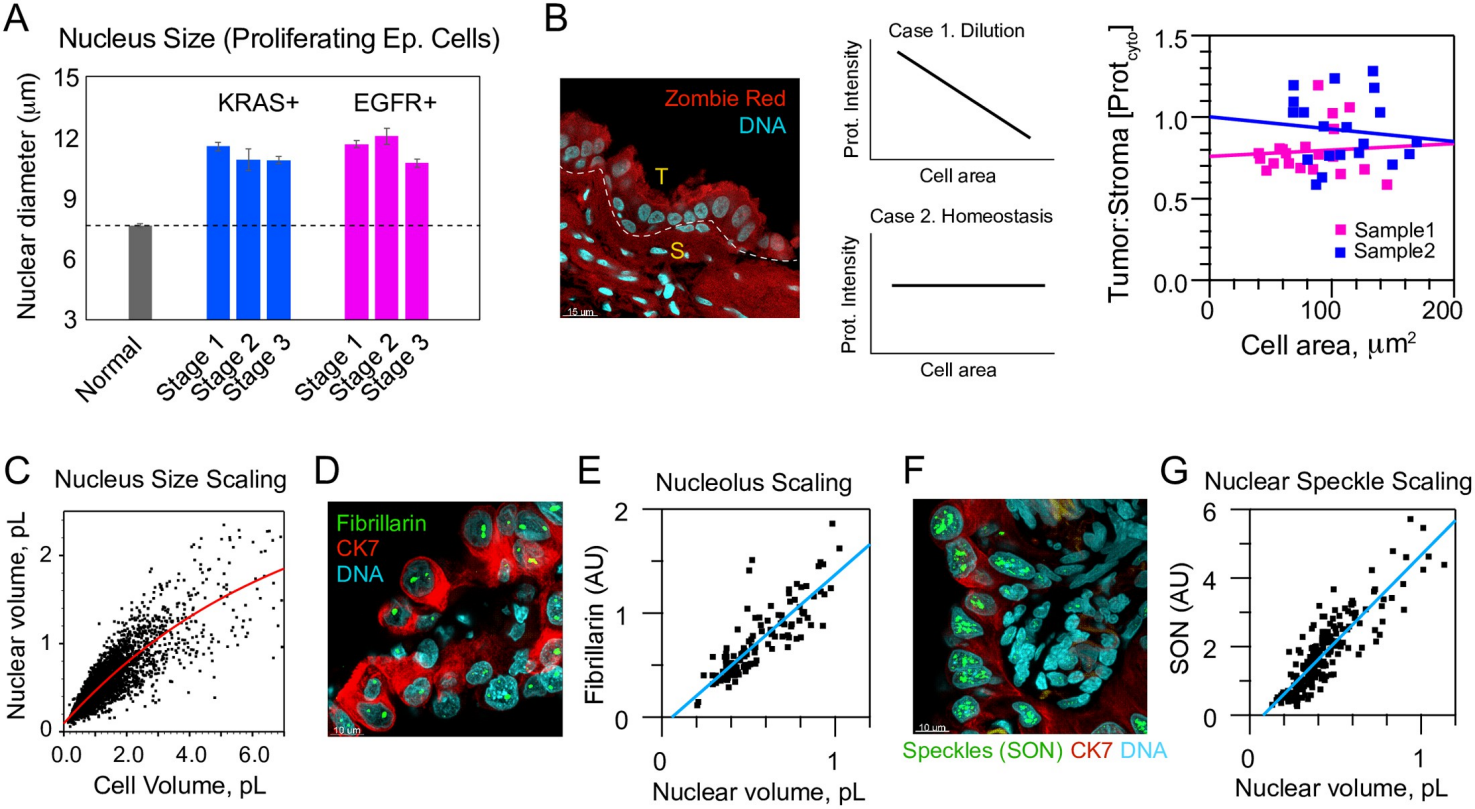
Enlarged EGFR+ or KRAS+ lung adenocarcinoma cells can proliferate, possess similar protein density to stroma, and display organelle size scaling. A. Nucleus sizes from proliferating EGFR+ and KRAS+ tumor cells identified by Ki-67 staining, relative to non-proliferative normal AT2 cells (N_EGFR_ = 8, N_KRAS_ = 8, n = 435). B., Zombie Red (red) is a broadly amine-reactive dye that in these FFPE samples reacts equally with small stromal cells, S, as it does with enlarged cancer cells, T, which is inconsistent with cells swelling via water uptake (N = 2). DNA stained with TO-PRO-3 (cyan). Right, Zombie Red intensity of KRAS+ tumors, a proxy for protein density in the cell, does not show the downward trend with cell area expected for water-diluted cells (N = 2, n = 20). C. Allometry of nuclear volume as a function of cell volume for LA (N = 21, n = 2082). Red line, double exponential fit to data. D., Fibrillarin (green) marks nucleoli comparing tumor cells of varying size in a KRAS+ stage 3 patient’s tumor (ID# 4855). CK7 (red), TO-PRO-3 (cyan). E., Total fibrillarin content in nuclei scales with segmented nuclear volume as expected for productive nucleoli (related to D., N = 1, n = 212). F., SON (green) marks nuclear speckles (also KRAS+ patient 4855). CK7 (red), TO-PRO-3 (cyan). G., Quantification of total SON content shows speckles also scale with segmented nuclear volume as they do in living cells (related to G., N = 1, n = 125).

The high prevalence of dysregulated cell sizes in LA suggests that these cells are contributing to tumorigenesis. However, an alternative hypothesis is that these enlarged cells are no longer functional or proliferative and perhaps senescent. To test this, we measured the average nucleus size of proliferating normal and LA cells across genotypes and stages. We found that the average nucleus size of proliferating cells was dramatically increased in LAs (Normal, 7.64 ± 0.04 μm, EGFR, 11.4 ± 0.12 μm, KRAS, 11.1 ± 0.12 μm error SEM, p = 2.5 x 10^−69^), suggesting that these enlarged cells are not senescent but are contributing to tumor growth (Fig 7A, S23 and S24 Figs).

A recent study proposed that water uptake is a consequence of aneuploidy, based on stiffness measurements of culture cells and plots of cell-size vs. ploidy for the NCI-60 panel [30, 31, 67]. To simplify our analysis of swelling, we considered two extreme cases. In the first case, water uptake fully explains cell volume differences predicting a linear negative correlation between protein density in cytoplasm and volume (Fig 7B, Case 1). In the second model, protein density is constant with cell size, implying no dependence (Fig 7B, Case 2). To test these models, we examined both intensities of Zombie Red, which reacts with all lysines, and CK7, a cytoskeletal protein that scales expression with cell volume [22]. The Zombie Red analysis shows that relative intensities in cytoplasm are not dissimilar from smaller, immediately-adjacent stromal cells (Fig 7B, compare S and T), but does not necessarily support either model. CK7 staining also supported neither swelling nor independence, but did suggest the very largest cells rarely possess the upper 50% of possible protein density (8.3% of cells >2 pL, 18.8% of cells ≤ 2 pL, S25 Fig). Rounded bodies and nuclei are another defining characteristic of turgid cells in hypotonic medium [68]. Such morphologies do exist within our samples, but are not conspicuous (e.g., a handful of nuclei in Fig 4A, center and right are representative). Consistent with this interpretation, cell model sphericity was 0.47 ± 7.1 x 10^−4^, with sphericities of ≥0.6 present in only 0.6% of all measurements (n = 4133, S1 Data, tab B). Similarly, lamin A+C staining of nuclei showed highly irregular surfaces (final portions of S2 Video, Fig 4 and S26 Fig). Nuclear models show a minority of oblate (15.0%) or prolate (26.0%), ellipticities less than 0.3. Overall, our data supports a view in which a minority of LA cells swell, but in a manner that is not proportional to cell size.

For enlarged cells to function and proliferate, they would need to expand their organelles and output of pathways for RNA production and ribosome biogenesis. We first noticed that enlarged cells properly maintained nucleus size scaling (Fig 7C). To determine whether nucleolus size and rRNA processing might be upregulated, we stained LA samples for fibrillarin. Indeed, we found a clear size scaling trend in which nucleolus size was increased in epithelial cells with enlarged nuclei (Fig 7D and 7E). To determine whether splicing and RNA production might also be upregulated in enlarged cells we stained for SON, a splicing marker that localizes to nuclear speckles [69, 70]. These nuclear bodies also showed a clear size scaling trend with nucleus size (Fig 7F and 7G). Together these data indicate that enlarged LA cells minimally maintain their protein and organelle homeostasis, scaling it as a function of cell size. These results contradict the notion that these enlarged cells are only swollen or senescent and hints at their higher functional output.

### Genomes of oversized lung adenocarcinoma cells are physically expanded

**Fig 8:**
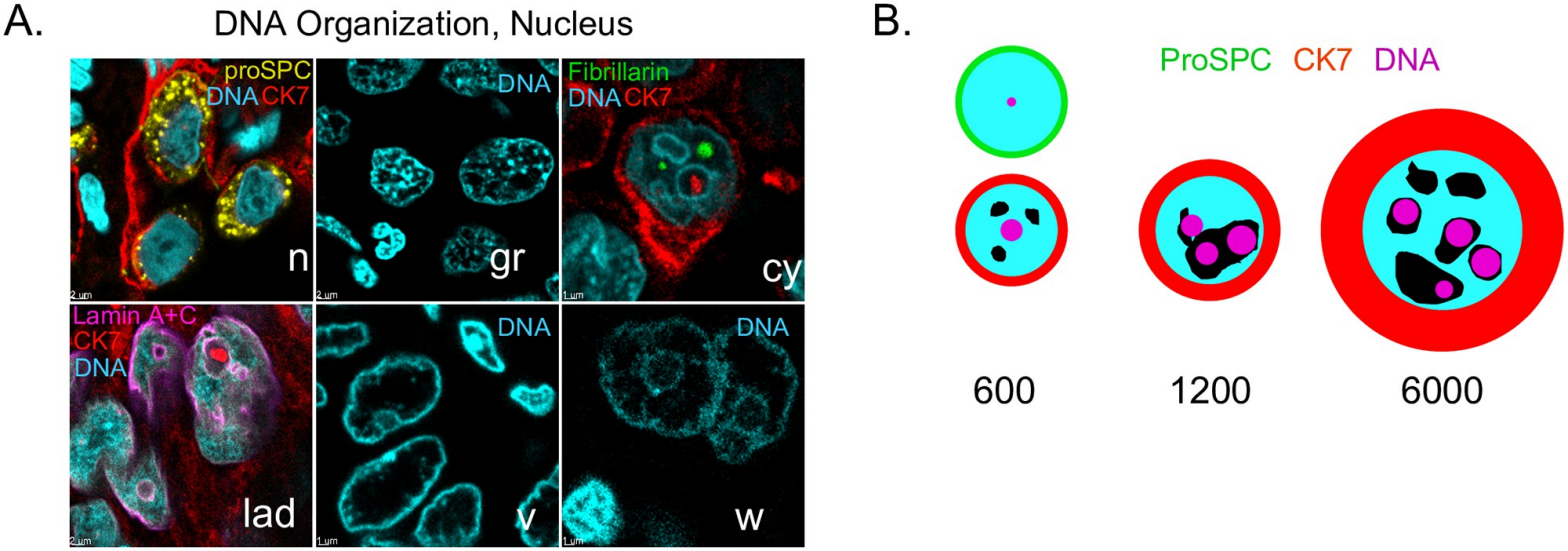
Diversity of nuclear organization and a revised 2D perspective of human lung adenocarcionoma. A., typical patterns of DNA staining observed in both EGFR+ and KRAS+ LA. n, normal AT2 cells with proSPC (yellow), CK7 (red), DNA (cyan). gr, granular DNA. cy, cytoplasmic inclusions, with fibrillarin (green). lad, lamin associated DNA, with Lamin A+C (magenta). v, DNA voids. w, “wagon-wheel” DNA. Idealized representations of nuclear structures shown for clarity. See Table 5 for associated patient ID information. B., Visual depiction of the relative equatorial cross-sectional area in μm^2^ of typical LA cells, if they were spheres, relative to the nucleus (cyan). green, AT2, cyan, DNA, black, DNA voids, magenta, nucleoli/speckles.

Given the correlations observed between cell and nuclear volume, and findings that changes in chromatin organization can be observed in cancer, we asked whether DNA staining displayed any special patterns in LA nuclei. We observe five common classes of DNA staining, and mixtures of these that describe most DNA data in LA (Fig 8A). Normal AT2 cells have mostly even DNA-staining profiles, with little granularity, and are so consistent that neoplasms are usually conspicuous. In LA, the most common pattern consists of a granular appearance suggestive of local heterochromatic regions. Cytoplasmic inclusions are ringed by concentrations of DNA at their boundary. Lamin-associated DNA is concentrated at the nuclear periphery and sometimes at the periphery of inclusions. DNA voids consist of large volumes that stain poorly for DNA, but are otherwise not significantly bordered. “Wagon-wheel” arrangements are reminiscent of nucleoli, but may not contain them, as suggested by weak DNA staining at their central “hub” regions.

We took particular note of Lamin association and thus we asked whether nuclear size dysregulation is correlated in any way with Lamin A+C wrinkling. We observe high Lamin A+C expression in all our patient samples, as has been observed previously for the LA subtype [71]. Lamin A+C is such a strong marker that it enabled segmentation of most of our dataset with the membrane detection algorithm of Imaris^™^. This algorithm creates objects that are significantly smaller than the nuclear volume, but contains very accurate shape information about the sphericity, i.e., wrinkling, of the nuclear envelope (S26 and S27 Figs). We reject any significant relationship between lamin wrinkling and nuclear volume, and by proxy cell volume (S28 Fig). However, Lamin A+C data support a similar trend as nuclear volume data with disease stage as measured by DNA thresholding, supporting that method (Fig 2D).

The presence of DNA-poor voids led us to ask whether genomes could be diluted in larger cells. To test this, we plotted the cell volume as a function of the ratio of ploidy:nuclear volume, where the latter tracks the amount of genome dilution (S14B Fig), showing that DNA in large LA cells >2 pL is diluted within the nucleus, consistent with voids.

Taken together with the above, and with the observations of lamin-associated DNA, we wondered if DNA movement to the envelope, sometimes associated with gene regulation, may be a hallmark of oversized cells [72]. To answer this question, we used the distance transform to create eroded shells of nuclei for four patients, found the amount of DNA within 1.5 μm of the nuclear surface, and then calculated the amount of nuclear-envelope enrichment of DNA as a function of cell volume. However, there is no trend with cell size, but shell-packing of DNA staining may be a signature of individual patients (S29 Fig).

Fig 8B summarizes how this study has revised our expectations about the 2-dimensional length scales of cell bodies, nuclei, nucleoli, and nuclear voids of AT2 or LA cells of different sizes, if cells and nuclei were actually perfect spheres.

### Conservation of size dysregulation in a mouse model lung cancer system

**Fig 9:**
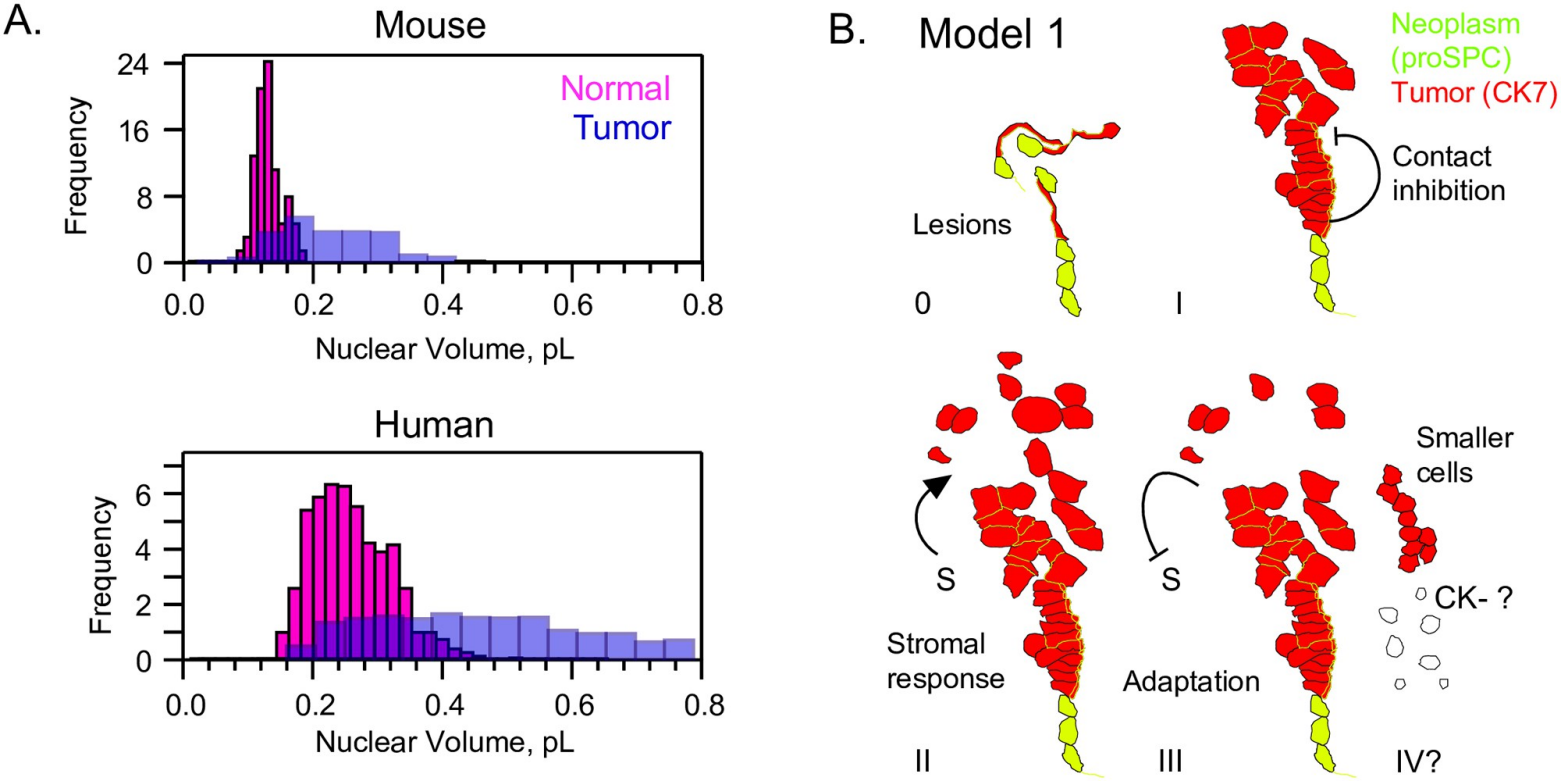
Epithelial cell-size dysregulation is also found in mouse tumors and may proceed with loss of tissue organization and upregulation of transcription. A., KrasLSL-G12D/+; p53fl/fl; Rosa26LSL-eYFP/LSL-eYFP mouse lung tissue shows nuclear size dysregulation, despite volumes of ~50% that of humans. B., Model 1: Oncogenesis is characterized by lesions, loss of flattening to an AT1 form and thus shortening of processes. At stage I, a contact-dependent mechanism preserves some cell size regulation. Stage II involves more mixed stroma/tumor regions and disorganization, leading to sparse cells and increased cell size dysregulation. Late stage (III-IV) cancers are dominated by adaptation towards maximal cell division rate, evolution of immune resistance, and also loss of cytokeratin staining. Smaller cells, and those of the more subproportional volume:ploidy relationship in KRAS+ cancers (Fig 3) may become enriched in late stages.

We were curious whether the phenomenon of cell and nucleus size regulation might be specific to human LA or might also be found in a mouse model of lung cancer. We segmented normal nuclei and also cancer nuclei from proSPC-staining cells in a KrasLSL-G12D/+; p53fl/fl; Rosa26LSL-eYFP/LSL-eYFP mouse. Our analysis showed that although mouse AT2 cells are smaller than human AT2 cells, both show loss of size homeostasis in cancer (Fig 9A). Mean nuclear volume for the mouse was 117.4 ± 21.36 (n = 58), 1.72-fold smaller than the first Gaussian of human AT2s. For tumor cells, which were filled with proSPC at all sizes, the nuclear volume was 254.7 ± 358.5 (n = 207), or 2.17-fold smaller than the overall human LA mean. Importantly, the mouse coefficient of variation for LA nuclear volume was 1.41, somewhat higher than the human average for all LA cells of 0.68. Altogether, the human model possesses a greater range of nuclear volumes and is more relevant to human disease, whereas the prevalence of volume variation in the mouse model provide opportunities for genetic characterization of pathways of size variation.

## Discussion

Our survey of authentic human tumors has underscored to us the limits of reductionist models in understanding a complex multicellular community. Clearly much is to be learned from in situ studies in 3D such as the one presented here [24, 25, 53]. Our findings are particularly relevant to recent discussions related to oncogenesis, giant superpolyploid cells (GSPPCs), and osmotic homeostasis [30, 44, 59, 73].

Although the oncogenic event that leads to LA is unknown, Lin et al. established in mice that AT2 cells can generate new tumors [44]. Our observations of proSPC staining support this association between AT2 cells and LA cells in humans. Others have suggested that the oncogenic event involves tetraploidy specifically [59]. Although we suspect this is true in some cases, our plots of cell-volume vs ploidy show that most cells are well within 2-4n range of DNA amount. Thus, if tetraploidy is the first oncogenic event then in our patients there must exist later events that enrich for lower DNA contents.

GSPPC’s are observed after cancer cell lines are subjected to chemotherapeutic drugs and ionizing radiation, and display persistent dormancy followed by a restart of division and even budding of multiple daughter cells [73]. This behavior supports a model wherein GSPPC’s cause recurrence of disease after treatment leads to remission. Our data do not rule out such a model, but show GSPPC’s are exceedingly rare observables in untreated LA patients (compare Fig 2B and S7 Fig and see Table 4). An upper limit for their prevalence could be estimated from Table 4, and cannot exceed 10% of abnormal cells in stage 2 EGFR. Thus, future estimates of the prevalence of GSPPC’s in treated patients may provide a more complete picture of the relevance of this phenomenon to disease.

Osmotic imbalance in cancer was not supported by our simple Zombie Red test or CK7 staining variation, or by nuclear rounding of Lamin (Fig 7H and S27 Fig). Thus, the model proposed by Tsai et al. that water uptake is increased in aneuploid cells could not explain our data as it could the data for yeast. Our own careful review of the Dolfi et al. data did not find a significant dependence of volume on odd ploidy number across the diverse NCI-60 panel [30]. The only trend we could find between aneuploidy and cell swelling in human LA was a depletion in CK7 staining in the very largest cells (S25, red box). Our data does not conflict with an assumption of nuclear stiffening of cancer cells however, if such stiffening can be explained by protein production, nuclear dilution, or RNA production (Fig 7B–7G).

Our 3D segmentation approach overcomes the general limitations of uniform 2D sampling, and is representative of a growing trend in which AI, combined with human expertise, can be leveraged to overcome methodological hurdles. We acknowledge that the resulting cell models are an approximation, and yet they provide an unprecedented level of detail with respect to measuring scaling changes of diseased cancer cells. Indeed, we observe slightly larger volumes for nuclei than can be estimated from nuclear areas reported by Nakazato et al. from paraffin blocks, which range from 242–508 fL. Taking the midpoint of that range to be 375 fL, and the midpoint of our range of nuclear mean volumes to be 650 for polyploid nuclei suggests the 2D approach underestimates the volume by the significant value of ~2-fold [17].

Our method required fixation and clearing for IF, so the volumes we report are smaller than those of live lung cells and their nuclei. Shrinkage artefact caused by neutral buffered formalin fixative is fairly small in an aqueous medium, ~3% for strips of rat kidney [54]. On the other hand, benzyl alcohol:benzyl benzoate (BABB) is reported to shrink tissue sections ~50%, with less data available for individual cells [74]. Exactly accounting for this shrinkage would have required live patient cells paired with each of our FFPE specimens. Yet size distributions of fixed tumor cells in aqueous buffer show similar size-change and size-distribution behavior as cells in BABB, suggesting, shrinkage affects large and small cells and nuclei to approximately the same extent (Fig 5D). In terms of staining, our approach was technically limited with regard to the use of the CK7 marker, and thus more work is required to characterize the scaling changes of cells that undergo loss of CK7 expression.

From the clinical perspective, KRAS+ patients displayed a lower N:C ratio at early stages (Fig 2D, bottom). Thus, careful cytometric statistics may be required for early KRAS+ cancers. Our data on individual patients’ cell volume-ploidy profiles support the important conclusion that observations of cell-size variation are not sufficient to conclude high ploidy overall. Our shell analysis of envelope packing also shows that patients that have DNA voids may fall into a distinct class (Fig 8A and S29 Fig).

Most importantly, our results using both microscopy and flow cytometry have shown that the influence of DNA amount on cell-size dysregulation and cell-size increase in LA is greatly overemphasized (Fig 2). A century ago and more, Levine and his contemporaries established the paradigm that “giant” cancer cells are of high ploidy, and raised the question whether such cells contributed to disease or were “amitotic”, i.e, irrelevant to disease [75]. To address these long-standing concerns, our study found that the size-ploidy paradigm holds for cells of volume 7–30 pL which are exclusively of the polyploid variety, but that the majority of LA cells are abnormally large, irrespective of ploidy (Table 4). These oversized cancer cells represent the vast majority of tumor cells throughout all stages, a situation sufficient to imply that they contribute to LA progression.

Cell enlargement is a consequence of cell growth, which takes place up until the start of cell division. Therefore, the enrichment of abnormal cells that is specific to stage 2 LA (Fig 2B) likely reflects changes in cell cycle length [4, 6, 76, 77]. Pauses in the cell cycle do occur during dormancy, one cause of which is proposed to be immune response [78]. In this context, it is therefore interesting to note that B cells and macrophages that may be competent to engage tumor cells are specifically enriched at stage 2 [79]. We hypothesize that stage 2 engagement of non-cancerous cells may lengthen the cell cycle for tumor cells, explaining some of the enlargement of near-euploid cells that is special to that stage.

Our analysis of proliferation using the Ki-67 marker established the existence of proliferating oversized cancer cells, but clearly, there exist many cells that are oversized but do not stain with Ki-67 (S23 Fig). These cells may be arrested in G0, and if so, whether or not they remain so may be relevant to some forms of chemotherapy resistance.

It remains an open question what pathways lead to giant near-euploid cells in LA. Some have proposed that increased volume supports additional glycolytic machinery as a consequence of the Warburg effect [80]. Some LA cells switch to a mucinous form, but these phenootypes were absent from our data [81]. However, our data support the idea that giant lung adenocarconima cells retain some aspects of a secretion genetic programme. We hypothesize that giant LA cells inappropriately overexpress factors related to secretion, in the absence of a delivery system for multilamellar bodies. More recently, it has also been proposed that p38 and Cdk regulate cell size, and that retinoblastoma may control a cell size set point. In addition, future work may clarify whether G1 length alteration contributes to the cell size enlargement observed in the LA cells characterized in this work [82–84].

Our new flow cytometry method for sorting tumor cells by size offers many new opportunities for studying such oversized cells and cell size dysregulation in disease. The method may be attempted with any epithelial cancer, if the SSC-W parameter is robust across cell types. We stress that our success with the method was a direct consequence of recommendations of Tzur et al. [63].

Cell density or packing appears to be a necessary condition for cell size regulation in LA. Combined with the observation that proSPC staining and processes are lost at cell boundaries, this strongly suggests loss of multicellularity in LA is sufficient for loss of epithelial determination. One might envision that our “shedding” and sparse size-dysregulated LA cells may be enriched with pioneers for escape to the nearby blood (S4 Video). It has also been observed that cells which lose rigidity sensing can become transformed and overgrow [85].

Although our study set out to study cell size, we were fortunate enough to discover networks of AT2 processes. These structures have likely gone unnoticed due to the specific markers required, their extreme fragility, and their requirement for an intact alveolus. A thorough search of the literature suggests that processes may have been observed, but were misidentified as part of other structures. In particular, Weibel refers to a ridge at the boundary of the AT1 cell body in a scanning electron micrograph, and clearly labels it part of AT1 cells [47]. However, our digests showed these structures can be biochemically isolated from AT1 cells and ECM, but remain attached to and linked between AT2 cell bodies, even as part of a CK7-staining structure (S10 and S11 Videos). We find no evidence that processes that surround AT1 cells terminate, although smaller “subprocesses” do so. We hypothesize that process networks work like a system of pipes to distribute surfactant over the alveoli of larger mammals, a model that has implications for any disease that involves surfactant. Their networking also suggests the possibility of intercellular communication. Perturbations of this network could have profound effects on normal lung function. Chronic diseases with variable amounts of scarring over time would likely interfere with and even destroy these networks of processes. Although further investigation of AT2 processes was beyond our initial scope, we suggest worthy future directions include study of living, intact AT2 networks by careful tracking of mature surfactant protein C, as well as examination of these structures in the context of other diseases [48].

While scaling of organelles with cell size in nature is well-established, our data has contributed to a perspective that cancer cells can possess scaling, albeit a type of scaling which is offset from that of normal cells. For LA cells, nuclei are swollen with nucleoplasm, and their nucleoli are aberrant in shape and number but otherwise scale appropriately with volume as do wild-type cells (Fig 7C, and S28 Fig shows an enlarged version).

Considering all our observations, we propose Model 1, which invokes a contact-inhibition mechanism–loss of contacts between cells may contribute to cell size dysregulation (Fig 9B). Before stage 1 in humans, cell size dysregulation is still mostly limited by contact inhibition and AT2-like differentiation. This intrinsic cell-size variation is not size dysregulation, but comprises ranges of cell size consistent with growth and division and remains as a component of all stages. By stage 1 in humans, loss of differentiation and organization leads to a population of solid and sparse cells with disrupted contact inhibition. In the absence of cell-cell contact, cells fully de-differentiate, reorganize their chromatin, and activate genes appropriate for a solitary life, as observed in loss of heterotypic contact-induced inhibition of locomotion, or the mesenchymal-to-amoeboid transition [86, 87]. Stage 2 cells are larger because immune responses are still competent and thus contribute to mixed presentation of stroma and consequently additional production of solitary cells. By stage 3, evolutionary adaptation results in cells that both compartmentalize to sheets and also divide at the maximal rate, producing the minimum degree of cytoplasm. If they are even smaller, from Fig 4B we could speculate that the end-point for stage 4 or in the most aggressive cancers may be mostly smaller sheet-like cells (Figs 4B and 9B, IV) [42]. It is an open question what the cell-size variation is in mesenchymal-like cells that do not express CK7.

We have shown that the near-euploid fraction of human and mouse LA are useful models for studying the molecular underpinnings of cell size dysregulation and variation. In combination with new flow cytometry tools we have suggested, these models provide a convenient means to study the genetic basis of cell-size regulation in mammals.

## Methods

### Patient samples, genotyping, formaldehyde fixation and paraffin embedding

Formaldehyde-fixed paraffin-embedded (FFPE) samples were kindly provided by the University of Pennsylvania Tumor Tissue and Specimen BioBank (TTAB). Samples included ten blocks from untreated patients genotyped as EGFR-positive/KRAS-negative and eleven blocks from patients genotyped as KRAS-positive/EGFR-negative. Staging was determined by standard measurements of tumor diameter and interpretation of H&E by a pathologist and were as indicated in Table 7.

**Table 7:** Stage, genotype, and pathological classification of lung adenocarcinoma patients.

Patient ID	Genotype	Stage
3437	EGFR+/KRAS-	IA
4838	EGFR+/KRAS-	1A
4837	EGFR+/KRAS-	1B
4839	EGFR+/KRAS-	1B
4842	EGFR+/KRAS-	2A
4840	EGFR+/KRAS-	2B
4841	EGFR+/KRAS-	2B
4843	EGFR+/KRAS-	3A
4844	EGFR+/KRAS-	3A
4845	EGFR+/KRAS-	3A
4848	KRAS+/EGFR-	1A
4849	KRAS+/EGFR-	1A
4846	KRAS+/EGFR-	1B
4847	KRAS+/EGFR-	1B
4850	KRAS+/EGFR-	2B
4851	KRAS+/EGFR-	2B
4852	KRAS+/EGFR-	2B
4853	KRAS+/EGFR-	2B
4854	KRAS+/EGFR-	3A
4855	KRAS+/EGFR-	3A
4856	KRAS+/EGFR-	3B

Fixation was performed in neutral buffered 10% formalin for a minimum of six hours, with all steps including stepwise dehydration, clearing with xylenes and paraffin-embedding performed on a commercial tissue processor. Processing was performed by the Hospital of the University of Pennsylvania Department of Pathology and Laboratory Medicine Division of Anatomic Pathology.

Normal distant control tissue was identified and marked by Dr. Feldman by inspection of H&E clinical slides. FFPE blocks were then subdivided, such that distant control regions were used for imaging normal AT2 cells.

### Dewaxing and sectioning

Up to 12 FFPE blocks, including 6 distant controls and 6 tumor specimens, were dewaxed for 24–72 hours in two six-well aluminum Reacti-blocks (Fisher, TS18811) immersed in 3 L of xylenes (Fisher Chemical, X3S-4). Samples were next washed once with 50% ethanol and then twice in anhydrous ethanol for storage at -20°C. Storage was for up to two months for principal IF in which channel 1 was proSPC, Lamin AC, Fibrillarin, or SON, up to six months for Ki-67 proliferation testing. S30 Fig shows that IF was stable over the period of principal imaging for both normal controls patient 4844, with 25X images shown in Fig 7B and S6K Fig representative of sample response after 6 months storage. For whole-mount sectioning, specimens were rehydrated to 50% ethanol for 5 minutes with nutation followed by one wash with water and one wash with PBS. Human specimens were sectioned to 150–200 μm on a Leica Vibratome (VT1000S) in PBS, mounted with superglue but otherwise with no solid or gelatinous infusion such as agarose, with maximum amplitude settings and frequency of from 50–100% depending on tissue softness. i.e., 100% normal lung distant controls required the highest setting. After sectioning, specimens were again dehydrated for storage at -20°C.

### Antigen recovery, immunofluorescence, clearing and mounting

All staged and genotyped tumor FFPE blocks were kindly provided by the University of Pennsylvania Abramson Cancer Center Tumor and Biospecimen BioBank (TTAB). FFPE sections stored in ethanol at -20°C were rehydrated as described. Antigen recovery upon 150 μm sections was performed in loosely capped 5 mL reaction vials using a Decloaking Chamber (BioCare Medical) at 110° for 37 minutes using a 10X TE buffer at pH 9 at a 1:100 dilution in dI water (Dako Target Retrieval). The sample was allowed to cool to room temperature and then washed 3 times with 0.5X SSC buffer (Corning). Samples were transferred to bleaching buffer (0.5X SSC, 2% hydrogen peroxide, 5% formamide) and bleached for at least 4 hours under a 130 V 100 W bulb at a distance of 20 cm at 4°C. Samples were returned to RT, then washed three times in quick succession and again every hour for four hours with PBT (PBS plus 0.1% triton and 2 mg/mL BSA). Blocking was performed for 2 hours at RT in blocking reagent with nutation. Blocking reagent was a 9:1 mixture of PBS containing 0.1% triton X-100 and no BSA and goat serum (abcam 7481). We stress that any BSA in blocking reagent specifically interfered with IF in our hands. Primary antibodies at the concentrations listed in Table 6 were applied in blocking reagent overnight in 2 mL glass reaction vials with nutation at 4°C. On the second day, samples were washed with PBT at room temperature on a rotator over a 7-hour period with the wash changed at least three times at intervals of at least 1 hour. Secondary antibodies as shown in Table 6 were applied overnight in the dark at 4°C in blocking reagent with nutation. On day three, the samples were again washed on a rotator for 7 hours in PBT. TO-PRO-3 at a 1:500 dilution in PBS was applied in one wash for 20 minutes on a rotator and then refreshed for 10 minutes such that a faded blue color remained in the solution, which we took as indication that DNA binding sites were saturated. To prevent washout of TO-PRO-3 stain that we have observed in mixtures of water/methanol, samples were immediately dehydrated to 100% methanol for 30 minutes with nutation, then washed again once with methanol and once with anhydrous methanol at intervals of 30 minutes. To clear tissue, samples were placed in 1:2 Benzyl Alcohol:Benzyl Benzoate (BABB) on a rotator in the dark for 10 minutes. Next the initial BABB was replaced, and samples were cleared overnight in a dark drawer. The next day the BABB was exchanged and the samples were cleared for another 24 hours. BABB was refreshed a final time at mounting. For mounting, samples were subdivided 1–3 times with a razor to lay flat, then placed in custom laser-cut chambers with 3.75 x 19 x 19 mm windows filled with BABB. Samples were pressed under a second 18 x 18 mm coverslip to lay flat before the chambers were filled to level. 40 μL of BABB was removed to leave a small bubble and ensure no overflow before the chamber was finally sealed with nail polish.

### Laser-scanning confocal imaging

Principal imaging relevant to all cell volume segmentation and high-resolution figures was performed at room temperature on a Zeiss LSM-880 with a Plan-apochromat 63X/1.4 oil DIC M27 objective with 0.19 mm free working distance, 0.17 mm coverglass thickness, 25 mm FOV, and 45.06 parfocal length. Scanning settings were 10 (fastest setting), with 900 x 900 pixels and 0.15 μm^3^ voxels, employing a 1X digital zoom. Laser lines were 488, 561, and 633 nm, employing transmittances of 5, 1, and 1% respectively, and each used a gain setting of 600.

Efforts were made to minimize sampling bias during imaging, but only regions staining brightly with either proSPC, for normal AT2 cells or mouse tumor cells, or CK7 (bonafide tumor cells) were imaged. To capture representative morphological patterns, a number of 25 X images were snapped to capture all the overall morphological variation present in a sample. This initial impression was used as a guide to gather from 1–4 scans at 63X until all the bright-staining representative morphologies available were captured. Any 63X image stack was constrained to include a minimum of 20 stromal nuclei controls for DNA amount. Therefore, cells with phenotypes which stain weakly with either proSPC or CK7 are not included in our data.

25X images were collected similarly to 63X images, except that the voxel size was 0.3779 μm^3^ and z-step of either 2 (Ki-67) or 0.5 μm (others).

### Attenuation correction and background subtraction of raw data

Attenuation is loss of signal as light scatters nonspecifically by interacting with matter as it passes through the object of interest. As a result, the signal from the fluorophores diminished as a function of z-depth. Data for channel 1 (488 nm) did not display any attenuation in our hands. Data for channels 2 and 3 displayed attenuation of differing degrees. To estimate the attenuation, images were opened in FIJI, and the mean intensity over at least 100 pixels was measured in a uniformly staining square region of a typical brightest object (e.g., nuclei) present near both the top and bottom of each image stack. For example, if a normal multilobed neutrophil nuclei was observed at the top of the stack that was the brightest observed, then a similar neutrophil was measured near the bottom of the stack that was also the brightest observed. If normal cells were not available at the top of the stack, then similar cancer cell features were selected, such as DNA concentrated near the outer edges of nuclei. Attenuation measured for channel 2 (CK7) was typically less than 20%, and attenuation for channel 3 was consistently about 2-fold over approximately 350 z-steps. To correct attenuation, mean intensities for top and bottom were used in combination with the Attenuation Correction MATLAB plugin for Bitplane Imaris 9.2.1. Background subtraction was performed in Imaris 9.2.1 using the Background Subtraction function in the Thresholding submenu of the Image Processing menu, with all three channels checked and the default parameter of 33.7 um filter width applied.

### Segmentation of normal AT2 cells using Bitplane Imaris^™^ software

Confocal IF image stacks of distant control samples stained with anti-proSPC and anti-CK7 were selected for segmentation if they contained alveolar arrangements in the CK7 channel. Cells were classified as non-neoplastic if they fulfilled the criteria of both presenting proSPC in a dense punctate arrangement outlining a cell body, extending into a network of long processes, as well as possessing signature DNA staining patterns lacking any inclusion structures, large voids, or granules (Fig 7H). 3D segmentation of cell bodies and nuclei was performed using standard manually guided procedures in the wizard of Bitplane Imaris^**™**^ 9.2.1, combined with some channel masking and erosion steps, and is detailed in the walkthrough in S4 Fig.

First, a surface object was built with a coarse level of 1 μm smoothing, with an isovolume threshold cutoff of from 7–12 applied to the proSPC staining such that the general form of the cell bodies and their nearby neighboring space was captured. Filters were applied to remove thresholded fragments of AT2 processes and cells at the borders of the imaging area. The resulting masked DNA channel highlighted inappropriately included neighbors and was used as a guide to erode the coarse SPC surface using the distance transformation plugin of Imaris^**™**^. The initial coarse cell body model was then eroded in 3D by from 0.7–1 μm, as minimally as possible, but as appropriate to minimize inappropriately included neighbor nuclei. This erosion of the coarse proSPC surface was then applied to mask the DNA channel, removing most of the inappropriately included neighbor nuclei.

The resulting masked DNA channel was used to create a near-accurate surface from both AT2 and some clipped neighbor DNA. To accomplish this a cell object was created with settings of 0.3 μm smoothing, threshold of 7, and splitting activated with 2 μm seed points and all seed points accepted. These surfaces were exported to a surface object and the resulting surface containing over-split nuclei and a few nearest-neighbor clippings were manually rejoined and shaped, using the editor with liberal use of the surface scissor tool, or deleted as appropriate, always with 3D visualization of the data as a guide. After manual editing, small clippings with near-zero volume or area resulted which were filtered and deleted. The resulting segmentable AT2 nuclear surfaces were then used as a final accurate mask for the DNA channel.

For final models of the cell body, the previously described erosion surface was used to mask the proSPC channel. This resulting masked channel for proSPC was added to the DNA channel that was masked previously by using the segmented AT2 nuclei. This channel addition was performed by using the channel arithmetics plugin. This final segmentation channel, which provides a full signal from the entire AT2 cell body, was used to create the 3D segmentations of the AT2 cell bodies. For this model of normal AT2 cell bodies, a cell object was created with the following settings. 0.3 μm smoothing, a threshold of 7–12 chosen with manual inspection using Imaris^**™**^ 3D view and wizard, and surface splitting with 5 μm seed points. Manual curation was performed on the cell body surfaces as described for the nuclei, with continuous reference to the unmasked SPC channel. To create fully segmented cells, a new cell object was created and both the cell body and nuclear surfaces were imported. S4 Fig summarizes this process and S1D Fig is representative of the high accuracy of this method relative to the pattern of proSPC staining.

We wish to note that AT2-like lesions or small neoplasms that present proSPC, but are sparse and border CK7+ tumor cells cannot be segmented by this method or the method described below for CK7+ cells, and are thus excluded from our analysis (S4 and S5 Videos).

### Image annotation for training set generation

To train our UNet-based convolutional neural network (UNet), we settled on an empirically guided, data-driven annotation methodology. All annotations were performed guided by the CK7 channel of an autoscaled merge of red CK7 and cyan DNA. We employed a conservative, low-inference boundary drawing approach, performed only on obvious cells with clear boundaries, which in tests produced the strongest predictions (S2 Fig). To establish a high-quality training set of 40 images, Dr. Sandlin annotated 80 images and a second expert in cell biology, Dr. Good evaluated the images with a score of 0–6 based on the representativeness and accuracy of the cell boundaries drawn. The highest-scoring 40 images from the original 80 with the clearest cell boundaries and brightest CK7 signals were selected for our final training set. Further testing revealed that masking the data supplied to the network external to the boundaries and eliminating non-cell marks improved prediction quality based on cell coverage and qualitative reduction in apparent false-positive marks (S1C Fig).

### Prediction of tumor cell boundaries using a 2D U-Net-based convolutional neural network

S1A Fig illustrates the architecture of the U-Net [88]. A deep convolutional neural network was trained with the aforementioned set of 40 images in order to detect cell boundaries. Training images with initial size of 900×900 were cropped to a size of 448×448 to obtain 160 images. Data augmentation was used to increase the size of this dataset in order to prevent over-fitting. This augmentation involves the application of rotation, shift, shear, zoom and flip to each annotation image. These operations increased the training set to 4960 images. This training set was normalized by dividing the 8-bit images by 255 and centralized by subtracting the mean over the z-dimension of pixel values for each image. In addition, the training set was split into 2 parts. 80% of the training set was used for training, and 20% for validation. A loss function was employed, with the form

$$L=\text{ylog}+\left(1-\overset{-}{y}\right)\text{log}\left(1-\overset{-}{y}\right),$$ (1)

where L is the binary cross-entropy. The optimizer used during training was Adam, and Dropout was used to prevent over-fitting [89, 90]. The criteria used to stop training and save the model was a cutoff for the loss function 1. of less than 0.001. UNet code is available on request from the corresponding author, and may be used with cytokeratin IF images to detect cell boundaries.

### 3D segmentation of tumor cells using Imaris software with UNet boundary predictions as input

S3 Fig illustrate the procedure. Cancer nuclei were manually annotated with a white mark in FIJI based on the presence of CK7 stain and recognition of abnormal DNA morphology. Cells which appeared cut off by the edge in the 2D image were not annotated. This white mark was used to approximate the positions of nuclear centroids using a custom MATLAB script to simulate an idealized spherical nuclear object with radius 5 or 10 pixels. Raw.czi files at 900 x 900 that were preprocessed as described and converted to.ims files were cropped to 896 x 896 by removing 2 pixels from each edge using the 3D crop interface in Imaris. The 896 x 896 U-Net prediction stacks and the idealized nuclei represented by white spheres were then imported into Bitplane Imaris^™^ 9.3–9.5. A cell object was created with the nucleus detection scheme, employing the aforementioned idealized nuclear marks with the U-Net predicted boundaries as an input for the nucleus channel. For nuclei detection, an object radius of 1.6 μm was used for 5 pixel radius spheres and one of 2.75 μm diameter was used for 10-pixel radius spheres. A smoothing factor of 0.3 μm was used in all cases and a threshold of 10 units was employed for the idealized nuclei isosurface. For cell bodies, we used the membrane detection algorithm. According to the training provided by Bitplane^™^, in the slice view, we measured the diameter across the smallest predicted cell boundary for a tumor cell and estimated an even value of 4–10 μm. Due to the high variability in the cell sizes in LA, a second fit was sometimes performed using a larger diameter of 4 um larger, although with this step we rarely observed an increase in yield of more than 1–2 successful cell models (<5%). All voids in the model with angular appearance fit by the cell module or those that touched the edge of the image were deleted, including all cells with more than an apparent ~10% of their volume cut off by the image edge. This cell object was exported as a surface object, made 50% transparent colored by ID and used to mask and duplicate the DNA channel. Using the 3D view, all the tumor cells that contained fragmented nuclei within them or extensive clipping into neighbors (~10% of a neighbor nucleus) were deleted as failed segmentations. Double nuclei were retained as correct if their boundaries were obviously fused or the overall morphology of channel 2 CK7 supported this choice, but in most cases double nuclei were deleted as failed segmentations.

A second round of manual curation involved creating a new cell object and saturating the CK7 and DNA channels (upper limits of 100 and 50% max, respectively). In combination with the clipping planes feature of Imaris^™^, we were able to visualize in the xy and xz or yz planes how the cell bodies as volume representations agreed with the CK7 and DNA staining. In this way, most inaccurate segmentations were removed from the data.

A third round of manual curation involved inspection of all cells with N:C ratios observed in the top 10% and bottom 10% of the data. These suspect models were revisited in 2D and 3D and were deleted if their models were erroneous in terms of significant overlap between nuclear segmentation and cell boundary, in either the 2D slice view or in the 3D Imaris slice view, in any of the X, Y, or Z directions (S3B Fig, frame 2).

A final round of manual curation involved generating 2D line representations every 5 μm along the z-direction and observing agreement between the cell body and nuclei segmentations and the channel 2 and channel 3 data. If significant errors in 2D area were noted, cell models were revisited using the clipping plane method in 3D as described and deleted if they disagreed with the UNet model or image data.

For a minority of cells with complex shapes that were viewed as erroneous in their initial models, but nonetheless possessed strong predictions, we attempted to recover the models for volume estimation by refitting the UNet boundary in a small ROI, and then combining the surfaces using the unify tool.

### 3D segmentation of tumor cell nuclei using Imaris^™^ software

Initial tumor cell body models were exported again as a surface object used to duplicate and mask channel 3 again. Due to the extremely crowded and irregular arrangements of tumor cells, even slight inaccuracies in cell-boundary prediction can still result in minor amounts of neighbor clipping. To eliminate the possibility of clipped neighbor DNAs affecting our nuclear segmentations, we masked these clippings with a new cell object, in a way that was similar to the procedure for AT2 nuclei. This clipping mask cell object was fit to tumor cell DNA with the following parameters: Cell body only detection, Smoothing 0.3 μm, Background subtraction 5 μm sphere diameter, cell body detection, threshold 1 unit, Split by Seed points 2 μm, with all seed points accepted. Clippings were thus negatively selected for and masked away via extensive manual curation. To accurately model nuclei, we found that our Lamin AC stain was insufficient, because the fitting algorithm takes the inner shell of the Lamina and always underestimates the size of tumor nuclei (S5 Fig, white surfaces). Instead, we chose to use the low-background far-red channel 3, strongly present in all our data. However, we found that the volume of an isosurface created in Imaris is dependent on the overall brightness of the image stack, as represented by the center-of-mass of the channel 3 histogram, as well as the threshold chosen (S5 Fig, magenta and red surfaces). To attempt to correct for this minor empirical drift, we sought a self-consistent rule by which to model the isosurface, in such a way that the surface would match the expected size and shape of nuclear envelopes, but also be of sufficient volume to encompass all the DNA data for quantification. To derive such a rule, we determined a linear relationship between the observed distance of a given isosurface based on DNA intensity, with dimensions that themselves depend on threshold, from an ideal Lamin AC surface. We now describe how we used available Lamin data to derive an empirical relationship between the threshold which we selected in Imaris to model nuclei, upon a given DNA channel intensity histogram’s center-of-mass.

Let t be the threshold selected to build an isosurface based on DNA stain, l the amount by which a lamin envelope surface is dilated to make a larger isosurface, and d be the DNA content inside of the resulting 3D surfaces. We approximate the DNA content as identical between these two isosurfaces, which are based on DNA or lamin dilation, for some corresponding values of t and l. For a given DNA channel histogram center-of-mass, I, the experimentally observed behavior of these dna-content functions, d, for small changes is approximately linear, obeying

$$d\left(t,I\right)=\beta_{t}(I)-m_{t}(I)t$$ (2)

$$d\left(l,I\right)=\beta_{l}(I)+m_{l}(I)l$$ (3)

I a constant for one image stack, where the β, m are the respective intercepts and slopes (see also, S5A Fig to understand the differences in sign). Restated in simpler terms, the dna content d inside each isosurface goes down when we increase t and up when we increase l.

We seek an empirical function for the dependence of the threshold we select, t, on some theoretical amount of lamin dilation (yet to be decided), which we will call L. From 2 and 3, we have rates

$$\frac{dd}{dt}=-m_{t}$$ (4)

$$\frac{dd}{dl}=m_{l}$$ (5)

Therefore,

$$\frac{dt}{dl}=\frac{dd/dl}{dd/dt}=-\frac{m_{l}}{m_{t}}$$ (6)

This provides the slope of the desired function. However, we need to determine the form of t(L), such that as L increases, t decreases. The intercept β_t_ represents the theoretical dna amount contained in the isosurface at zero threshold and as *L* → *∞*. This intercept is also the maximum value at which d(l) should be defined, because it is the amount of dna contained in a nucleus within a 3D reconstruction of infinite size. For L = 0 (no dilation of the lamin-based surface), we have d = β_1_, which we know to underestimate the real amount of DNA (see S5A Fig, Lamin) This results in range and domain for L and d of

$$L\in \left[0,\frac{\beta_{t}-\beta_{l}}{m_{l}}\right]$$ (7)

$$d\in \left[\beta_{l},\beta_{t}\right]$$ (8)

From these values we can use 2 to determine a range for our desired function t(L).


$$t\in \left[0,\frac{\left(\beta_{t}-\beta_{l}\right)}{m_{t}}\right]$$

So the form of the function we are constructing should depend on L, take on maximum value $\frac{\left(\beta_{t}-\beta_{l}\right)}{m_{t}}$, and have slope $-\frac{m_{l}}{m_{t}}$. Because m_t_ is negative, for clarity we use the absolute value $\left|\frac{m_{l}}{m_{t}}\right|$ below. This gives the function

$$t\left(L\right)\equiv \frac{\left(\beta_{t}-\beta_{l}\right)}{m_{t}}-\left|\frac{m_{l}}{m_{t}}\right|L$$ (9)

The dna content value we report for tumor cells in this work is

$$d_{90\%}=0.1\beta_{l}+0.9\beta_{t},$$ (10)

arbitrarily chosen as 90% of the theoretical maximum DNA that would be associated with zero threshold and infinite volume. S5A Fig illustrates how this choice qualitatively fits nuclei for different histogram brightnesses. The associated dilation amount, L, calculated from 3 and 10 is

$$L_{90}=\frac{0.9\left(\beta_{t}-\beta_{l}\right)}{m_{l}},$$ (11)

we calculated the appropriate thresholds associated with these values by substituting 11 Into 9, giving

$$t_{90}=\frac{\left(\beta_{t}-\beta_{l}\right)}{m_{t}}-\left|\frac{0.90\left(\beta_{t}-\beta_{l}\right)}{m_{t}}\right|$$ (12)

To determine the relationship between t90 in Eq 12 and histogram brightness, I, we chose several files of varying brightness and measured the DNA content at different thresholds and lamin dilation amounts. Dilation was accomplished using Bitplane Imaris with the surface object and distance transform XTension plugin. By averaging the regression slopes and intercepts across all fit nuclei in a stack, we were able to solve for the parameters of interest. S5B shows the nonlinear regression of the data, based on the model

$$t\left(I\right)=0.6+k_{1}e^{k_{2}I},$$ (13)

where the choice 0.6 was based on the observation of a breakdown in segmentation at thresholds below 0.6, which approach the level of the most common background noise after background subtraction (S1 Data, tab A). A fit to the six test files provided, spanning the available intensities provides the empirical formula 14 used for all subsequent threshold calculations for tumor nuclei.


$$t\left(I\right)=0.6+(0.219\pm 0.062)e^{(0.0665\pm 0.005)I}$$ (14)

Once this threshold was calculated, the masked tumor DNA were fit using cell body detection with the calculated threshold and 0.3 μm smoothing and 5 μm radius for background subtraction. To account for cavities that could be created using this method that would under-estimate the DNA amount, an additional step to fill holes was included (S3B Fig, panels 7.-8). The preliminary segmentation described above was used to mask a white channel, and these objects were subjected to the fill-holes binary operation slice-by-slice using FIJI. The resulting solid objects were imported back into Imaris, and the binary, filled tumor nuclei were fit to an isosurface with 0.3 μm smoothing and automatic settings. S3B Fig, panel 9. shows that this second isosurface is only slightly larger than that of the empirically thresholded original, except for the filled cavities which represent a much more significant volume change (blue vs. white surface). To provide normal controls, normal nuclei were fit using identical settings, except that they were automatically split with seed points of diameter 3 μm. The normal nuclei were exported from a cell to a surface object for curation, in which normal nuclei were filtered for a distance greater than 4 μm from an edge and lack of CK7 staining. At least 20 normal nuclei were measured in order to estimate the mode of the normal DNA as a control for tumor dna amount, with the modes taken from a Gaussian fit to the DNA histograms. All statistics for tumor cells and normal DNA were exported as.csv files, and these data and derived parameters are included in supplemental spreadsheet 1.

### Gentle dissociation of live and fixed human tumor specimens and labeling for flow cytometry and sorting

All tumor specimens were kindly provided by TTAB. For live tumor resections, material not required for pathological analysis was weighed and within 4 hours of removal placed in RPMI media at 4°C. Except for the following changes, gentle dissociation procedures were as described in [65] which shows that the enzyme cocktail used preserves the CD45 receptors present on blood cells. To minimize aggregation and clustering of cells further, we supplemented the cocktail with 0.3 mg/mL hyaluronidase (Worthington). 25 mL enzyme solution per g tissue was used, with an incubation period of 2 h 20 minutes and a shaker speed of 95 rpm. Post incubation, mechanical dissociation was performed with 25 expulsions of the 10 mL serological pipette at the most acute angle possible with the wall of the falcon tube.

All centrifugation steps utilized a Sorvall Legend RT centrifuge employing a swinging-bucket rotor at 1400 rpm for 5 minutes. After filtering the resulting digest with a 70 μm cell strainer, cells were resuspended in 25 mL of 1X RBC lysis buffer at RT for 9 minutes. The reaction was quenched by adding 25 mL PBS, the reaction gently inverted 3–4 times, volumes split into two 25 mL aliquots and cells were resuspended in 1–3 mL PBS. Cells were stained with 1:100 Zombie Red (BioLegend 423109) in the dark in PBS for 10 minutes, then resuspended in PBS for up to 10 minutes. To count cells, a sample was taken on a hemacytometer and cells were imaged on a spinning disk confocal with bright field and 561 nm excitation channels. At this stage the experiment was stopped if >30% dead cells were observed in the red channel.

For pellet-free fixation, cells were combined, in order, with volumes of cold Fluorescence-activated Cell Sorting buffer (FACS, PBS, 0.5% BSA, 2 mM EDTA), PBS, and PBS containing 10% formaldehyde to achieve a final concentration of 2% formaldehyde, 0.1% BSA, 0.4 mM EDTA, and less than 10 million cells/mL. Immediately on the last addition of formaldehyde at 10% concentration, the tube was capped and gently inverted continuously while being placed on a nutator. The fixation reaction was allowed to complete with nutation in the dark at RT for 15 minutes. To quench formaldehyde, we then added an equal volume of 150 mM glycine at pH 7.2 [66]. The quenching reaction was capped and gently inverted, then rocked on a nutator for 10 minutes in the dark at RT. In anticipation of RNA sequencing attempts, we added 1 tube of Superase In RNase inhibitor (ThermoFisher Scientific) to 2.5 mL of FACS buffer at 4°C, and washed cells twice with this solution before washing cells twice with cold FACS. In the final volume appropriate for MACS cells were resuspended in FACS containing this RNase inhibitor.

Cells were subjected to MACS purification using human CD45 beads and LS columns for depletion (Miltenyi Biotec) according to the manufacturer’s instructions, except for the following changes. During CD45 bead incubation, after 10 minutes a mouse anti-EPCAM-AlexaFluor488 antibody (BioLegend, Table 6) was added at 1:50 along with 1:500 DRAQ5 (BD Biosciences 564902). The tube was flicked 6–10 times until bluish throughout and incubated on ice for an additional 10 minutes. 1–2 mL of FACS buffer was added and the labeled cells centrifuged at 300 g for 10 minutes on a Sorvall Legend RT prep centrifuge with a swining-arm rotor.

The supernatant was completely removed and FACS was added to achieve a concentration no higher than 50 million cells per mL. A 40 μL sample of this parent population was saved for later imaging and a portion of this diluted with FACS to measure intensity histograms and calibrate sorting gates (Fig 5A). The supernatant of the parent digest was inspected visually for large particles and if necessary cells were strained again with 70 μm cell strainer.

MACS LS was performed as instructed by the manufacturer except that after a single wash with FACS, a plunge of the CD45 positive cells was performed with 5 mL of FACS and immediately used to load a second equilibrated column. This second column was washed twice with FACS and both CD45 negative fractions were combined and the CD45-positive plunge collected. 40–100 μL samples of CD45-negative and CD45-positive fractions were saved for later imaging. Samples saved for imaging were stored at 4°C for up to six days.

CD45-positive and–negative fractions were concentrated if necessary on a centrifuge.

### Sorting epithelial tumor cells by size using the Aria sorter via Live/Dead, EPCAM, DRAQ5, and SSC-W gating

All flow cytometry and sorting was performed using a BD FACS Aria II SORP with violet, blue, green, and red solid-state lasers and 14 color parameters, controlled by BD FACSDiva software. Laser calibration of fluorophore gates was performed using the parent sample, and laser lines employed were the 515/20 (495–525 nm), 610/20 (600–630 nm), and 660/20 (650–670 nm). The FSC and SSC lasers were calibrated in a standard fashion to enable maximal coverage of the data and prevent saturation of the upper limit of the SSC-H vs SSC-W plot (appropriate calibration is shown in Fig 5B). Compensation was not required due to high separation of the fluorophores employed, AlexaFluor-488 (EPCAM), Zombie Red (Live/Dead), and DRAQ5 (DNA). Gates were defined exactly in the manner shown in Fig 5A employing the signal histograms. The Live/Dead gate was drawn using the parent digest, with the upper limit intersecting the left slope of the positive signal at ~FWHM. The EPCAM gate was drawn using the CD45-negative sample, and intersected the valley between the obviously enriched positive and negative signals. The DNA gate was drawn using the CD45-negative fraction, such that the upper limit of the low-DNA gate was set to 1.5X the mode of the DNA signal. This was observed to mark a “shelf” suggestive of polyploid cells, and was settled on because higher levels allowed the appearance of doublets and higher order aggregates in the imaging data.

Size sorting of tumor cells employs the SSC-H vs SSC-W plot with ungated CD45- cells. Size gates were developed using an empirical curve based on the measured area of cells in imaging from gates, and the boundaries used produced optimal sorting (Fig 5 and S16 Fig). SSC-H boundaries were trimmed from 50–200 based on preliminary observations of aggregates arising from those regions, and size gate ranges are as shown in Fig 5D (G1 =“Smallest”, 64–79, G2 =“Small”, 84–99, G3 =“Large”, 101–113, G4 =“Largest”, 114+). After successful DNA gating, gate G4 contained sparing aggregates, cells with protein attached, and very usual cell shapes with very low yield (the remainder taken by subtracting the fractions G1-G3 in S17 Fig from 1). If DNA gating was unsuccessful, cell aggregates became obvious in G3 and rare giant superpolyploid cells could sometimes be observed in G4. We found overall that developing a figure like S16 Fig and quantifying aggregates was essential to calibrating our size sorting, as inspired by [63].

### Time-course videos of gentle digestion of FFPE extracellular material to isolate AT2 processes

Distant control and tumor FFPE blocks from a patient diagnosed with a highly invasive LA were provided by TTAB and was processed with IF as described, up to the point right before dehydration. Instead of methanol, tissue sections were immersed in custom round imaging wells 3.75 mm deep and filled with a 180 μL volume containing 1 part PBS and 1 part 1 mg/mL collagenase/dispase preparation (0.5 mg/mL final total enzyme content, Roche, 10269638001), also containing 1 mg/mL hyaluronidase (MolBio). To visualize DNA, 20 μL of 20x Hoescht 33342 was added at time zero. Reactions were allowed to proceed at room temperature for 100 minutes before imaging on a spinning-disk confocal microscope with a 60X/water objective at various times over a period of six additional hours. To prevent evaporation and inhibit any spurious protease activity upon processes, a different version of FACS buffer from that used with MACS (PBS, PH 7.6, 3 mM EDTA, 2% BSA) containing complete EDTA-free protease inhibitor (Roche) was added every two hours in dropwise quantities of less than 50 μL sufficient to maintain fluid level. To provide very gentle loosening of sections, upon addition of protease inhibitor/FACS a pipette tip was very gently moved back and forth in the liquid of the well at a position at least 2–3 mm from the tissue section. S6–S13 Videos were created using Bitplane Imaris^™^ in the 3D perspective view after background subtraction as described and after autoscaling of all channels. The small scale bars shown in the videos are for the point closest to the viewer in the perspective.

### Spinning-disk confocal imaging of flow cytometry digests and FFPE digests

Flow cytometry digests and gentle digestion kinetics were performed at room temperature with an Olympus IX81 inverted microscope with 405, 488, and 561 laser excitation lines, employing an Andor iXon3 EMCCD camera and controlled by Metamorph software. Flow cytometry digests and resultant sorted cells from gates G1-G4 were imaged with a 20x/air objective. Gentle digest kinetics were imaged with a 60x/water objective with 0.5 or 1 μm z-steps and varying time steps of from one second to one minute to document essential features of the phenomena shown in S6–S13 Videos.

### 2D analysis of long-axes of Ki-67-staining nuclei

Immunofluorescence, clearing, and imaging was performed as described above, except that imaging was at 25x magnification with a z-step of at most 2 μm and an imaging depth of greater than 200 μm. The rabbit marker used in this case was the highly specific marker Ki-67 marker for active DNA synthesis, as listed in Table 6 [91]. Tumor regions were selected for imaging if they possessed positive CK7 staining in a majority of the imaging volume and if they contained more than one of the developmental morphologies defined in the results. For example, if sheet and sparse (Fig 4) were observed in the same region this region was preferentially imaged over a region with only sheet. Sixteen image stacks were collected spanning eight from the EGFR group and eight from the KRAS group and stages 1–3 represented. The resulting images of nuclei and proliferation markers were segmented in order to mask irrelevant signals using Bitplane Imaris^™^ 9.2.1. The following parameters were used for channel 3, stained with TO-PRO-3. Cell body detection, Smoothing = 2 μm, Background Subtraction Sphere Diameter = 5 μm, Cell Threshold = 7.00, Split by Seed Points = 7, Filter by Quality selected manually with the lower threshold at the maximum of the displayed histogram. The resulting Imaris cell object segmentation was exported as a Surface object and filtered for all surfaces containing a sphericity > 0.6. Nuclear masks were also excluded if they were within 10 μm of the edge of the imaging volume.

To identify CK7-positive nuclei, a CK7 surface with default smoothing (.756 μm), Background Subtraction checked, Diameter of Largest Sphere which fits into the Object determined by measurement in the slice view, and lower threshold of 3 units was generated and made 50% transparent. Elements of this CK7 staining visual guide were excluded by a filter if they were of a smaller volume than 600 μm^3^, the typical volume of a wild-type epithelial type II cell, or they were manually deleted if they appeared to be disconnected from the major CK7 regions and were judged to be irregular elements of autofluorescence artefacts. A filter was applied to the nuclear Surface object for total CK7-staining units and the threshold of this filter was adjusted until nuclei were highlighted only within the CK7-thresholded region. Using this procedure, nuclei below 3 x 10^4^ total units of CK7 signal were always observed to be outside the envelope defined as the CK7-region eliminated from analysis. In some cases single nuclei were identified as non-CK7 by eye and deleted manually. The remaining nuclear surfaces were defined as cancer nuclei, and these were used to mask both the DNA channel and the Ki67 channel. The masked channel 1, which represented both proliferating and non-proliferating cancer cell nuclei was segmented using a cell object with settings of Cell Body Detection, Smoothing Filter Width = 1 μm, Background Subtraction not checked, threshold 20, and split by seed points = 7 μm, seed point quality = default, with only Cell objects with total Ki-67 > 2 x 10^4^, sphericity >0.5, and volume > 150 μm^3^ retained. These highly actively synthesizing nuclei were defined as the most stringently proliferating cancer nuclei and a surface object based on this cell object was used to mask channel 1 again. This channel masked to include only the highest Ki67 signals was exported to a.tif file along with the DNA channel masked only for the property of being CK7-positive. This 2-channel.tif was opened in FIJI and converted to a hyperstack, and the lookup tables changed such that the DNA channel was displayed as red and the Ki67 channel displayed as green. the stack was made into a color composite and converted to an RGB image. A set of integrated z-projections were created encapsulating each Ki-67 positive nucleus, and the cross-sectional area and long and short axes were measured using the polygon and line tools. Representative z-projections of the nuclei measured along their long axes are shown in S23A Fig.

### Estimation of fibrillarin and SON amount and nuclear volumes in human and mouse lung epithelia

Immunofluorescence was performed as described, with channel 1 stains for the appropriate markers applied using conditions shown in Table 7. Segmentation of nuclear volumes for the depictions shown in Figs 7E, 7G and 8A were performed using the Bitplane imaris^™^ surface tool using straightforward thresholding approaches to generate isovolume surfaces for nuclei, followed by splitting using the surface scissors tool to separate any closely apposed nuclei. Settings were as described above for cell segmentations, except the isovolume threshold chosen was 1 in all cases.

### Analysis of DNA enrichment near the envelope of tumor nuclei

Representative files with tumor cells segmented as described, and from each of the listed patients in the legend of S29 Fig was used to create an eroded set of nuclear volumes using the distance transform tool, with 1.5 μm distance erosion. This surface, representing the core of the nuclei was associated with the original, non-eroded nuclear segmentations. To calculate shell enrichment, the total amount of DNA in the core was subtracted from the total DNA in the nucleus, and the concentration of DNA in the shell was related to the concentration of DNA in the nucleus as a whole. The enrichment shown in S29 Fig is the ratio of those two densities.

### Plotting software, general figure notes, and figure graphical enhancements

Figs 1A, 2A, 3A, 4A and 9B, Model 1, were created in Adobe Illustrator. Fig 2B histogram bin regions were colored in red and green for clarity using Adobe Illustrator with the paint bucket tool. Fig 7A was recolored separately in Adobe Illustrator and overlaid for clarity. Scatterplots and histograms were plotted using Igor Pro 6.35A. Violin diagrams in S9 Fig were plotted using Graphpad Prism^™^ 8.0. Fig 5A–5C and S17 Fig were created using FlowJo 10, and Fig 5A was colored-in using the Adobe Illustrator Paint Bucket. Boundary lines calculated from 2D segmentations in S2B Fig were calculated using a custom MATLAB script and displayed using Bitplane Imaris^™^ in the slicer view. All images shown of normal and tumor lung were captured using Bitplane Imaris^™^ using either the 3D view in orthogonal mode, for the case of z-projections, or using the slicer view for 2D depictions of single slices.

### Statistics and quantification of percent abnormal cells

Mean, standard deviation, interquartile range and maximum values for volume data were calculated using Igor Pro 6.35A for histograms and plots, or with Excel for supplemental spreadsheet 1. Histogram bins were calculated using the Freedman-Diaconis rule, where bin-width = 2 x IQR/N^⅓^. Non-parametric ANOVA and Mann-Whitney difference statistics for S9 Fig were calculated using Graphpad Prism^™^ 8.0 with the significance values shown, where all “***** entries have p-values <0.0001. The standard deviation of the fold-change was estimated using the Taylor approximation, assuming independent variables,

$$Var\left[\frac{X}{Y}\right]\approx \frac{1}{{\overset{-}{Y}}^{2}}s_{x}^{2}+\frac{{\overset{-}{X}}^{2}}{{\overset{-}{Y}}^{4}}s_{y}^{2}-2\frac{\overset{-}{X}}{{\overset{-}{Y}}^{3}}Cov\left[\frac{X}{Y}\right]\approx \frac{1}{{\overset{-}{Y}}^{2}}s_{x}^{2}+\frac{{\overset{-}{X}}^{2}}{{\overset{-}{Y}}^{4}}s_{y}^{2}$$ (15)

Where X and Y correspond, respectively, to the tumor and normal mean parameters and s is the sample standard deviation. The error in the fold change is reported as standard error, approximated as,

$$SE_{FC}=\frac{\sqrt{Var\left(\frac{X}{Y}\right)}}{\sqrt{n_{x}^{2}+n_{y}^{2}}}$$ (16)

Categories of subproportional, proportional or supraproportional cells, were classified simply according to their ratios of their cell model volumes to estimated ploidy. These CV/n values were compared to the μ+σ and μ-σ cutoffs for Normal AT2 cells, assuming 2n ploidy, from the first Gaussian fit of the mixture model (S1 Data, Tumor Cell Data, columns AI and AM, related to Model statistics and fitting tab, Cell V56 ± Cell W56/$\sqrt{2},\;$ see below). Thus, if CV/n < (582–127)/2 = 227, then a cell was called subproportional, and this is the slope of the lower dotted line in Fig 3A, S13 and S14 Figs. Similarly if CV/n > (582+127)/2 then a cell s called supraproportional.

Categories of subproportional, proportional, or supraproportional for patients for Fig 3B were assessed by a conservative compound statistical test defined as follows and illustrated in columns Q-Z of the Patient statistics tab of S1 Data. A chi-square test for the number of cells observed in each category of S13 and S14 Figs against the null distribution of uniformly distributed cells was performed (Columns Q-W of Patient statistics). If the null hypothesis was retained (even distribution) with α = 0.05, then a patient was categorized overall as “Proportional”. If the null hypothesis was rejected (non-uniform distribution), then the supraproportional cells were compared to the subproportional cells using a binomial test with probability 0.5 and α = 0.05 (Column X of Patient statistics). If the null hypothesis was retained a second time (patient 4854 was the only case of this), the patient was categorized as “Proportional”. Otherwise, the patient was categorized as “Abnormal” and potentially in the supraproportional or subproportional categories. If neither the supraproportional or subproportional bin was 2-fold enriched over the other (Columns Z and AA of Patient statistics), then the patient was still categorized as proportional. Otherwise, the patient was categorized simply according to the cell bin with the highest number of cells. For example, if a patient has a non-uniform distribution of cells, and the number of cells in suprapraportional cell bin was 3-fold enriched over subproportional cells, the patient was called “Supraproportional”. On the other hand, if that patient only had 1.35 enrichment in the supraproportional bin, it was still called proportional. See also, S1 Data, Patientwise statistics, columns Q-AB, where cyan cells indicate progression through the decision rule.

Least-squares regression fits to distributions of cell and nuclear volume and N:C ratio used three competing models, all three are invoked only as empirical models for fits to tumor cells, as we assume a host or ensemble of cells living in a community. For normal AT2 cells, we assume a model with two populations with Gaussian distributions related to 2n and 4n.

Regression model 1, two-Gaussian mixture, empirical except for normal cells

$$Y\left(k_{1},k_{2},x_{1},x_{2},width_{1},width_{2},x\right)=k_{1}e^{-{\left(\frac{x-x_{1}}{width_{1}}\right)}^{2}}+k_{2}e^{-{\left(\frac{x-x_{2}}{width_{2}}\right)}^{2}}$$ (17)

Regression model 2, Single Gaussian, empirical

$$Y\left(k_{1},x_{1},width_{1},x\right)=k_{1}e^{-{\left(\frac{x-x_{1}}{width_{1}}\right)}^{2}}$$ (18)

Regression model 3, Lognormal, empirical

$$Y\left(k_{1},x_{1},x,width1\right)=k_{1}e^{-{\left(\frac{\text{ln}\frac{x}{x_{1}}}{width_{1}}\right)}^{2}},$$ (19)

where the baseline was fixed to zero in the regression. The choice of each model is justified using the rationale briefly recorded in S1 Data, and initial guesses are also recorded therein. We report the parameters from the regression and use the 2n populations as the normal control Gaussians for our main figures (S1 Data). The width parameters from these Gaussians (S1 Data, Model statistics and fitting tab, Cells W56-W57) are used to calculate σ as,

$${\left(\frac{x-x_{1}}{width1}\right)}^{2}=\frac{1}{2}{\left(\frac{x-x_{1}}{\sigma }\right)}^{2}={\left(\frac{x-x_{1}}{\sqrt{2}\sigma }\right)}^{2}\Rightarrow \sigma =\frac{width1}{\sqrt{2}}$$ (20)

For example, 179 was the fitted value of width1 for the first gaussian peak of AT2 normal cell volumes under the 2-gaussian mixture model (S1 Data, Model statistics and fitting tab, cell W56), which gives σ = 179/1.414 = 127. This value is used to formulate the upper and lower bounds of proportional cells for Fig 3A and 3B and for classification of patients in S13 and S14 Figs.

For quantification of percent abnormal cells, an image of a fitted curve was generated and the areas not within the regions specified by the normal cell gaussian were selected using the magic wand tool in FIJI, and measured against the total area under the curve. To clarify analysis, we approximate the standard error of the curve by that traditionally used for that of a proportion,

$$s_{prop}=\sqrt{p\left(1-p\right)}/\sqrt{n}$$ (21)

i.e., as though we could actually count cells that were outside the normal gaussian, where n is the number of cells in each stage (Table 4).

### Alignment of mouse and human Pulmonary Surfactant-Associated Protein C N-terminal sequences

Amino acids 1–100 from sequences with Uniprot ID’s P21841, MDMSSKEVLMESPPDYSAGPRSQFRIPCCPVHLKRLLIVVVVVVLVVVVIVGALLMGLHMSQKHTEMVLEMSIGAPETQKRLAPSERADTIATFSIGSTG, and P11686, MDVGSKEVLMESPPDYSAAPRGRFGIPCCPVHLKRLLIVVVVVVLIVVVIVGALLMGLHMSQKHTEMVLEMSIGAPEAQQRLALSEHLVTTATFSIGSTG, were aligned using the Needleman-Wunsch Global Alignment Tool at NCBI (https://blast.ncbi.nlm.nih.gov/) with the default parameters Existence: 11, Extension: 1.

### Rationales for selection of images shown in the figures

Normal distant controls: These cells displayed no noticeable trends with genotype or stage of the patients with respect to cell or nuclear size or staining phenotype. A perusal of S1 Data, tab C across patient IDs should convince the reader that normal distant controls are highly invariant in terms of scale. Any images of normal distant control samples were selected based on the ease of viewing particular features or with respect to their ability to provide high contrast and detailed depiction of clearly-staining features such as cell bodies or processes. For normal cells, CK7 staining was assessed for its ability to display alveolar physiology (e.g., Figs 1A and 6).

Fig 1C: The tumor sample shown possesses many features we commonly expected to see when seeing a sample for the first time, including granular TO-PRO 3 staining, fibrous-looking red CK7 staining, nuclei about the size of normal AT2 cells, stromal cells nearby, and some process-like staining with proSPC at cell boundaries.

Fig 1D: The tumor sample shown was selected due to the fact that its flat structure greatly facilitates depiction of the cell boundary simulation by our machine learning algorithm and it allows the reader to gauge for themselves the accuracy of the overall method. Furthermore, it was used during early development of the machine learning algorithm as a testbed and displays a sheet morphology present in about half of our image stacks.

Fig 3D: Cells were selected with a volume:ploidy proportionality and X,Y values close to the center of the datasets shown in 3B. 2D images were selected to display both the cell of interest and their neighborhood and do not show the widest cross-section, showing that the 3D segmentation provides a more accurate depiction of the cell size than the 2D view.

Fig 4A: Growth types shown were observed in tumors regardless of patient genotype, and were selected due to the clarity with which the sheet, solid, and sparse behaviors could be distinguished, as well as the staining quality. Features were also very carefully selected to give the reader a greater sense of our authentic experience of discovery at the microscope. These include the elongated nuclei of the stroma in the sheet depiction, the metaphase nuclei seen in the solid depiction strongly suggestive of proliferation and vitality of the tumor cells, and the doublet nuclei with inclusions seen in the sparse depiction.

Fig 5E: Spinning disk microscope images were first filtered for their depiction of easily distinguished round cells with good brightfield contrast. Cells within these “pretty” images were measured and the mean cell area within a given image was matched as closely as possible to the mode of the peaks shown in the bottom three rows of Fig 5D, Tumor.

Fig 6: RAGE, Tumor was chosen due to its depiction of a tumor-like phenotype, easily distinguished from the Normal alveoli structure above it. Neither genotype of tumor displayed any RAGE expression. proSPC, tumor were selected to display the complexity of the process structure and the greatest diversity of the proSPC staining within the tumor structures for both genotypes. We felt this was especially important because this aspect of human lung physiology has never been reported before.

Fig 7B, 7D and 7F: Figures were chosen for their capacity to convey the specific features being discussed or studied. 7B was selected because it shows that stromal cells have the same protein distribution as giant cancer cells. 7D was selected because it adequately displayed nucleolar staining behavior and was typical of such images. 7F was selected because it displayed speckle-like structure typical of such images.

Fig 8A: Features shown were observed in both genotypes and were chosen as idealized examples to demonstrate existence of the phenomena as they are depicted, and for ease of reference for those wishing to follow-up on these observations.

## Supporting information

S1 Fig Overview of machine learning approach to predict cell boundaries. A., Our machine learning approach employed a UNet algorithm, trained with manual annotations of cell boundaries. Both inputs and outputs of the algorithm are 2D pixel arrays, which involve a downsampling leg to conserve computational resources (left side of “U”) and an upsampling leg to recover positional information (right side of “U” and grey arrows). Data in a region local to a pixel of interest is used to generate a confidence score for that pixel that describes the likelihood that a cell boundary would have been drawn by the human annotator. This 2D array of confidence scores is the output for each of the ~250–500 images per stack, and represents a simulated cell boundary channel. B., representative manual annotations of cell boundaries, used to train the neural network. C., data showing marginal improvement of cell coverage which resulted by masking data outside of the annotation region. Cell models, y-axis, varied with cell density in stacks, x-axis. D. AT2 cell (proSPC) raw staining, left, segmented using Imaris to reveal individual cell surfaces, middle, which accurately predict cell boundary outlines (white), shown in 2D slice right. E. CK7 stain, red, left, after machine learning and segmentation of cell surfaces, middle, correctly predict cell boundaries of tumor cells (white); shown in 2D slice, right, and are comparable in accuracy to Imaris segmentation shown in D.
(PDF)

S2 Fig Representative 2D depictions for stack 4843T1, comparing annotations, data, UNet predictions, and Imaris surface fits. Cells passing manual curation display agreement between manual annotation, UNet prediction, Imaris 3D surface fit, and IF data. Yellow, annotation. White, Imaris fit to cell bodies. green lines, UNet predictions. Magenta, nucleus fit. Green data, Lamin A+C. Cyan data, DNA. Red data, CK7. Related to Figs 1D and 4A, left, S1 Fig, top, and S1 Video.
(PDF)

S3 Fig Walkthrough of Bitplane Imaris fitting of UNet predictions, quality checking, and fitting of nuclei to masked and filled tumor DNA. See Methods for detailed walkthrough. A: 1., CK7 data which was annotated by hand was used to train UNet, which, 2., predicts cell boundaries at every slice. 3., centers of nuclei are marked and transformed to spherical white regions to provide an idealized nucleus to guide the Imaris fitting algorithm. 4., Imaris fits the UNet-predicted “ribs” of each cell model. 5., Imaris creates volumes out the edges of the 3D stack volume, which are deleted. 6., The initial Imaris model contains both correct and incorrect cell models. B: 1., nuclear segmentation and all available IF staining and UNet predictions were used to assess the accuracy of every cell model in the X, Y, and Z view using clipping planes in Imaris. 2., cell models which disagreed with IF or UNet channels in Imaris or obviously conflicted with nuclear segmentation by >10% volume were discarded. 3., example of a lower-quality cell model which barely passed agreement with manual curation, presenting clipping of the cell body model with neighboring stromal nuclei. 4., view of the conflict in the DNA channel that illustrates the lower-limit of acceptance for model quality. 5., to quantify DNA accurately, any DNA clipped into a model were masked, first by creating a volume and then by deleting the data in Imaris. 6., illustration of a low-quality cell model with nucleus containing masked DNA associated uniquely with the cell model. 7., initial nuclear volume rendering, based on initial binary segmentation of the DNA channel made offline in FIJI. 8., a cavity present in the binary DNA channel was frequently seen in the larger nuclei of tumor cells, and was filled-in using FIJI to eliminate the void (related to Fig 8A). 9., after curation, overall agreement between Imaris models of cell bodies and nuclei, taken together with UNet and DNA channels is re-assessed. Blue and white, insignificant changes in volume and shape occurred after cavity-filling using FIJI. 10., typical overall appearance of coverage of cell models passing curation, green, and or failing curation, red.
(PDF)

S4 Fig Annotated walkthrough of Bitplane Imaris segmentation procedures for proSPC-staining Normal AT2 cells. 1. A coarse mask, translucent surfaces, is generated using a smoothed isosurface of proSPC data, green. 2., masked proSPC data contains some small process fragments which are further masked away. 3. DNA, cyan, is masked with the coarse proSPC model and contains inappropriate DNA clippings from abundant stromal cells. 4. a clipping model is generated, and clippings retained so that they can be deleted from the DNA channel. 5. over-split surfaces are joined using the unify tool. 6. joined DNA isosurfaces created using the unify tool in Imaris. 7., masked normal AT2 DNA, magenta, is added to 8., masked proSPC, to create a segmentation channel, 9. 10., 9. is used to generate the final cell body model using an isosurface guided by eye using the Imaris wizard. Scale, 20 μm.
(PDF)

S5 Fig Lamin A+C-based envelopes provide a minimum volume upon which to base an empirical model for DNA isovolume thresholds. For a DNA isovolume-based 3D surface, the observed volume is a function of the threshold selected (e.g., 0.6, 3, shown) and the brightness of the DNA. A., 1 μm projections associated with files containing three levels of DNA intensity. Surfaces, isovolumes of DNA with thresholds 0.6, red and 3, magenta, Lamin A+C model, white and 1 μm dilation of same, green. Cyan surface shows thethreshold selected, based on B. B., empirical fit to six files, see methods, Eq 14.
(PDF)

S6 Fig Polyploid and euploid cell volumes binned over all stages. Top, Normal AT2 cells (n = 802). All cells, N = 21, n = 4082. EGFR+, all ploidies, N = 10, n = 2194. KRAS+, all ploidies, N = 11, n = 1939. All euploids, cells with estimated ploidies of from 1.6–4.4n (n = 2683). EGFR euploids, n = 1411. KRAS euploids, n = 1260. Fits are a Gaussian mixture model, top, and lognormal, other histograms. Small Gaussians overlaid are the first Gaussian from the mixture model fit to normal AT2 cells, corresponding to the 2n population. Goodness-of-fit statistics available in S1 Data, tab G.
(PDF)

S7 Fig Polyploid and euploid nuclear volumes binned over all stages. Top, Normal AT2 cells (n = 802). All nuclei, N = 21, n = 4082. EGFR+, all ploidies, N = 10, n = 2194. KRAS+, all ploidies, N = 11, n = 1939. Euploids, nuclei with estimated ploidies of from 1.6–4.4n (n = 2671). EGFR euploids, n = 1411. KRAS euploids, n = 1260. Fits are a Gaussian mixture model, top, and lognormal, other histograms. Small Gaussians overlaid are the first Gaussian from the mixture model fit to normal AT2 nuclei, corresponding to the 2n population. Goodness-of-fit statistics available in S1 Data, tab G.
(PDF)

S8 Fig Polyploid and euploid N:C ratios binned over all stages. Top, Normal AT2 cells (n = 802). All nuclei, N = 21, n = 4078. EGFR+, all ploidies, N = 10, n = 2194. KRAS+, all ploidies, N = 11, n = 1884. Euploids, N:C ratios from cells with estimated ploidies of from 1.6–4.4n (n = 2668). EGFR euploids, n = 1411. KRAS euploids, n = 1257. Fits are a Gaussian mixture model, top, and lognormal, other histograms. Small Gaussians overlaid are the first Gaussian from the mixture model fit to normal AT2 cells, corresponding to the 2n population. Goodness-of-fit statistics available in S1 Data, tab G.
(PDF)

S9 Fig Polyploid cell volumes by stage and genotype. Related to Fig 2B, histograms of cell volumes for all segmented cells regardless of ploidy (N = 21, n = 4082). Fits, upper left, Gaussian mixture model, other fits, lognormal. Small Gaussian fits, first 2n population from a mixture-model fit to normal AT2 cells. Goodness-of-fit statistics and parameters from regression fit to normal AT2 cells as well as sample sizes by stage and genotype available in S1 Data, tab G.
(PDF)

S10 Fig A., Polyploid (n = 4082) and B., euploid (n = 2671) nuclear volumes by stage and genotype. Small Gaussian fits, first 2n population from a mixture-model fit to normal AT2 nuclei. Fits with two apparent modes, two-gaussian mixture models, other fits, single gaussians. Goodness-of-fit statistics and parameters from regression fit to normal AT2 cells as well as sample sizes by stage and genotype available in S1 Data, tab G.
(PDF)

S11 Fig A., Polyploid and B., euploid N:C ratios by stage and genotype. Small Gaussian fits, first 2n population from a mixture-model fit to normal AT2 cell nuclei and cell bodies. Fits, top row, two Gaussian mixture, other fits, lognormal. Goodness-of-fit statistics and parameters from regression fit to normal AT2 cells as well as sample sizes by stage and genotype available in S1 Data, tab G.
(PDF)

S12 Fig Cell volumes of each EGFR+ patient as a function of ploidy. "-", " = ", "+", classifications based on cells with sub-, green, proportional, blue, or supraproportional, red, behavior, used to construct Fig 3B. See Methods and S1 Data tab E for classification regime. Each dot represents a single cell measurement (n = 4082, related to S1 Data, tab B). Rows, stages 1–3. Some outliers are excluded from the edges of the plot for clarity and their data can be found in S1 Data, tab B.
(PDF)

S13 Fig Cell volumes of each KRAS+ patient as a function of ploidy. "-", " = ", "+", classifications based on cells with sub-, green, proportional, blue, or supraproportional, red, behavior, used to construct Fig 3B. See Methods and S1 Data tab E for classification regime. Each dot represents a single cell measurement (n = 4082, related to S1 Data, tab B). Rows, stages 1–3. Some outliers are excluded from the edges of the plot for clarity and their data can be found in S1 Data, tab B.
(PDF)

S14 Fig Cell proliferation and genome dilution in lung adenocarcinomas. A., Ki-67 proliferation marker is observed in cells across the range of volumes for LA. Green, Ki67, with merge to CK7, red and DNA, cyan at right. Quantitation of nuclear size data in proliferating cells is shown in Fig 7A, and related to S23 and S24 Figs. B, Estimated ploidy per unit volume within the nucleus, or genome concentration, plotted as a function of cell volume shows that nearly all cells with a volume greater than 2 pL display diluted genomes. n = 4082, related to S1 Data, tab B.
(PDF)

S15 Fig Smaller cells possess more elongated bodies and nuclei than larger cells. Left, plot of cell elongation, defined as the ratio of the prolate ellipticity to the oblate ellipticity, as a function of cell volume suggests smaller cells are more elongated, as they are observed in sheet growth (Fig 4 and S1 Video, and related to Fig 9B). Right, cell and nuclear elongation are weakly positively correlated (R^2^ = 0.21).
(PDF)

S16 Fig Cell area is proportional to SSC-W. Y-axis, the mean cell area collected from a SSC-W gate. X-axis, the midpoint of the size gate used to sort cells. For reference, an area of 100 μm^2^ is equivalent to a spherical volume of 0.75 pL, and 400 μm^2^ is equivalent to a spherical cell of ~6 pL Note also that the x-intercept is near the lower limit of detectable SSC-W. Fit, linear regression with fit parameters and errors as shown. All data is for near-euploids as described in Fig 5. N = 4 patients, with three size gates each. Error bars, SEM.
(PDF)

S17 Fig Cell-enlargement of near-euploid epithelia cells is a signature feature of resected lung adenocarcinoma tumors that can be detected using the side-scattering parameter in flow-cytometry. Related to Fig 5. CD45-/Zombie Red-/EPCAM+/Near-euploid cells gently dissociated from normal, top, or tumor, bottom surgical resections were further split according to side-scattering width (SSC-W). SSC-W gates: 1 = 64–79, 2 = 84–99, 3 = 101–113. Bars represent the fraction of all cells in gates G1-G4 for SSC-W vs SSC-H (See Fig 5B). Gate G4 for SSC-W is not shown, and contains sparing aggregates and debris. Right plot, mean of all fractions found in G3, containing the largest singlet cells. Error bars, SEM.
(PDF)

S18 Fig SSC-A vs FSC-A plots for samples at various stages of our protocol to sort tumor cells by size. Parent, the digest stained before MACS CD45+ depletion. Plunge, the column retentate containing CD45+ and some CD45- cells. Flow-through, CD45- cells. Zombie Red -, EPCAM+, Euploids, related to the corresponding gates shown in Fig 5A left-to-right. G1-G3, size gates for SSC-W, related to Fig 5B, show the expected increase in FSC-A and SSC-A with increasing cell size occurs, but data resolution and range is not optimal.
(PDF)

S19 Fig Only a handful of examples of flat-looking tumor cells were observed. A., z-projections at 63X magnification of flat-looking cells are still many times the thickness of an AT1 cell, suggesting if any developmental programme remains for flattening, it is altered from the wild-type. B., corresponding cell models. Scale, 20 μm.
(PDF)

S20 Fig Polyclonal proSPC antibody ab90261 is a sensitive and necessary marker for observing AT2 process networks in human lung. Green, proSPC. h, rabbit antibody ab90716 (abcam) raised against residues 1–100 of human proSPC. m, rabbit antibody ab3786 (Millipore Sigma) raised against residues 1–32 of human proSPC, but previously used in mouse studies. A., antibody and concentration (2000, 200, 20 show dilutions) dependence of IF signals from process networks. Antibody h showed minimal background staining even at high concentration, but nonetheless showed some evidence of processes even at low concentration. The m antibody could not detect processes in humans at the standard 200:1 concentration. B., process networks are not visible in mouse lung using the antibody raised against residues 1–32 at the standard 200:1 concentration. C., titration of the h antibody in mouse, showing that no process-like structures are visible in mouse even with nonspecific binding. D., a zoomed-in view of the C., h200 panel with the upper threshold reduced from 255 to 50 units still does not reveal any processes in mouse. Scale bars, 20 μm.
(PDF)

S21 Fig Behavior of AT2 process networks at various scales in human normal and tumor lung tissue and relation to cell-cell junctions. A., zoom views of proSPC, green, and CK7, red, showing how process networks follow the network of intracellular cytokeratin without displaying consistent colocalization (yellow in merge, relative to green in merge). This behavior may be expected in the case of multilamellar vesicles, structures which explain the puncta observed in AT2 cell bodies. White arrows, the distinctive pathology of AT2 processes in tumor cells is a progressive depletion of multilamellar bodies (puncta) and enhancement at cell-cell junctions. Neighbors often display a gradient in vesicle expression, from cells that appear AT2-like, to those with weak process staining and aberrant-appearing paths (see also, S4 and S5 Videos). Scale, 5 μm. B., colocalization of proSPC, green, and E-cadherin, red, is partial at the cell boundary, but “subnetworks” and what may be terminating branches are apparent (right image, darker regions). The “loop” that is visible is typical of the shape of large, flat, AT1 cell bodies in these alveoli. Scale, 20 μm.
(PDF)

S22 Fig proSPC processes tend to disappear with loss of cell-cell contact. 25x single images illustrating how variations in local cell-cell contact relate to variations in process phenotypes in LA. Sparse tumor cells, arrows, lack process-like proSPC expression relative to their more well-packed neighbors. green, proSPC, red, CK7, cyan, DNA. Top-left, top-center, and lower-right, EGFR+. Top-right, lower-left, and lower-center, KRAS+ (See Table 5 for all Patient ID associations). Scale, 50 μm.
(PDF)

S23 Fig Immunofluorescence of Ki-67 shows proliferation of epithelial cells showing size dysregulation. KRAS+ patient ID’s as indicated, with behaviors shown observed in both genotypes. Green, Ki-67, red, CK7, cyan, DNA. Bottom row, a higher density scan of the sample for patient 4846 displays the structural details of unusual proliferation centers in dividing tumor cells of varying size and DNA content. Scale, 50 μm, top and middle rows, 10 μm, bottom row. Normal AT2 cells have such a small fraction of proliferating cells that they were undetectable in the objective.
(PDF)

S24 Fig Proliferating nuclei display stage and genotype-dependent length changes. An alternative 2D-based approach was implemented as an expedient way to ascertain whether tumor nuclei classified as proliferating were also longer than normal AT2 nuclei. A., 2D projections of proliferating nuclei, illustrating typical measurement of the long axis, with dimensions near the means shown in B (see Methods). Green, Ki67, red, DNA.” Each row’s images are from a different patient. Scale bars, 10 μm. B., Mean long axis of 2D projections of proliferating nuclei as a function of genotype and disease stage. Black point, normal AT2 nuclei, which proliferate minimally in healthy tissue. Error bars, SEM.
(PDF)

S25 Fig Cytokeratin 7 concentration in tumor cells >2 pL does not reach high values seen in smaller cells, yet it is also not proportional to size. X-axis, intensity of CK7 IF measured in the cytoplasm of cells, employed as a proxy for concentration of intermediate filaments, expected to scale with cell size [22]. X-axis, cell volume. Inset, region discussed in text that is depleted of data, >127.5 A.U. CK7 in cytoplasm and >2 pL volume. 15.5% of data points shown are >2 pL, whereas only 1.3% of all data points are in the region shown (only 8.3% of cells >2 pL). In contrast, 18.2% of cells ≤ 2 pL display such high intensities. n = 4020, from analysis in S1 Data, Tab I.
(PDF)

S26 Fig Lamin A+C envelope volumes are maximal at stage 2 of lung adenocarcinoma, whereas wrinkling increases slightly in stage 3 EGFR. Top, histograms of volumes of nuclei, as reported by direct 3D segmentation of available Lamin A+C staining using Imaris software (S5 Fig, white surface cutaway). Data is of slightly smaller magnitude than that from DNA thresholding, but also supports specific enlargement of nuclei at stage 2 (S7 Fig, see Methods and S1 Data, tab G). Bottom, Lamin A+C surface segmentation effectively captures shape information about nuclear envelopes, as indicated by the reported sphericity, x-axis, as a function of genotype and stage. EGFR+ stage III notably displayed lower mean ± SD sphericity (μ = 0.810 ± 0.055) than other treatments (all other μ ≥ 0.816) with EGFR+ showing lower mean sphericities in all stages than KRAS+ patients (see panels). EGFR,+ left, and KRAS+, right, with disease stages as shown. Sample sizes: N, biological replicates, n, number of envelopes segmented. N = 21, n = 5668. N_EGFR_ = 10, n_EGFR,S1_ = 1272, n_EGFR,S2_ = 807, n_EGFR,S3_ = 1132, N_KRAS_ = 11, n_KRAS,S1_ = 524, n_KRAS,S2_ = 974, n_KRAS,S3_ = 959.
(PDF)

S27 Fig Lamin A+C envelope wrinkling is only weakly associated with nuclear volume. Related to S26 Fig, plots of the volume of the surface segmentation of Lamin A+C staining as a function of the sphericity reported by Imaris software, binned by genotype. Left, EGFR+, right, KRAS+. Lamin A+C Inner Volume = the volume of the envelope built from the Lamin A+C channel, which is significantly smaller than the volume of the nucleus as calculated from DNA thresholding (S5 Fig and Methods). Both large and small nuclei display a range of sphericities, which is not consistent with rounding of nuclei due to nuclear swelling.
(PDF)

S28 Fig Allometry of the nuclear volume as a function of cell volume for EGFR+ and KRAS+ lung adenocarcinoma. This figure is an enlargement of main Fig 7C. Red line, double exponential fit to data, with the equation and fit parameters shown on the plot. N = 21, n = 4082 Because cell volume mostly explains nuclear volume, organelle scaling is a feature of LA.
(PDF)

S29 Fig Envelope-localization of DNA is independent of cell size, but may vary between patients. X-axis, shell-enrichment, was defined as the calculated mean intensity of fluorescence within 1.5 μm of the surface defined by the tumor cell nuclear model, divided by the mean intensity not in this outer shell. DNA enriched in the shell does not explain cell volume. Colored dots, data from individual patients. R^2^: 3437, 6.46 x 10^−5^, 4842, 6.58 x 10^−5^, 4850, 5.65 x 10^−3^, 4852, 0.25. However, shell enrichment overall differs between patients. p-values less than 0.05 from Student’s t-test: 3437 vs 4850, p = 5.4 X 10^−7^. 4842 vs 4850, p = 1.05 x 10^−12^, 4850 vs 4852, p = 3.6 x 10^−9^.
(PDF)

S30 Fig Storage in anhydrous ethanol at -20°C preserves immunofluorescence signals from cytokeratin 7 and DNA. A., Images for control patient 4844 capture typical variability observed with immunofluorescence. Red, CK7, cyan, DNA. B., kinetics of overall stack intensities, as measured by the center-of-mass of the pixel intensity histogram, neither significantly decreased nor increased over the a two-month period when tissue sections were maintained dehydrated and at freezing temperatures.
(PDF)

S1 DataThis spreadsheet contains all data produced through the tumor cell segmentation project achieved through machine learning and employing Imaris, and contains additional statistics that may be of interest to researchers.Worksheets contain general statistics and fitting information for all treatments, genotypes, patients, and stages we interrogated. Calculation of DNA content (ploidy), the antecedent stromal controls and general statistics for all conditions are included in their own worksheets in this file. Each worksheet’s contents are described below: **Tab A. Stromal DNA controls**. This worksheet contains DNA-content data for segmented “stromal” (non-tumor) nuclei associated with each Image stack ID, which is directly related to tab B., Tumor Cell Data. Nucleus ID, the ID# given to each nuclei segemented by the Imaris software. Nuclear Area, the surface area of the nuclear segmentation object, an isovolume based on DNA thresholds. Nuclear Channel 2 intensity mean, the total intensity of CK7 IF inside the surface, is expected to be an insignificant value for non-tumor cells. Channel 3 Nuclear sum, the total intensity of DNA IF inside the surface. Nuclear voxels, the number of voxels counted by Imaris software inside the surface. Nuclear volume, the volume of the surface. Channel 3 Background Estimated, the value for any voxel used for background-subtraction for a given image stack. Normal Nuclei BS DNA, the total DNA IF intensity, column F, minus the total of all background inside the surface (voxels x background per voxel). Voxel volume, the local voxel volume reported by Imaris software. **Tab B., Tumor Cell Data**. Patient deID, the identifier given to each set of paired samples from a patient specimen. Genotype, EGFR+ or KRAS+ genotyping information provided by TTAB. Disease Stage, staging information on the specimen provided by TTAB. Differentiation, description of the pathological classification of differentiation, provided by TTAB. Image Stack ID, the unique filename identifier associated with each image stack. Growth Phenoypes Observed in Stack, related to Fig 4 and discussion, whether Sheet, Solid, Sparse, or giant cell phenotypes were specified as existing within a given image stack. Cell ID, the unique identifier given to each cell model by Imaris. Cytoplasm Channel 2 Intensity Mean, the intensity per voxel on average within the cell body, but not contained within the nuclear model, if present. Cytoplasm Volume, the volume of the cell body surface model, less the volume inside the nuclear model, if present. Cell Ellipticity (Oblate or Prolate), shape parameters provided by Imaris for the given cell surface model. Cell Sphericity, shape parameter provided by Imaris for the given cell surface. Cell volume, volume of the cell surface model. Columns P-R and W, similar to cell data, but for nuclear surface models, when present. Nuclear Channel 1–2 Intensity Means, control data showing average proSPC, Lamin A+C, or CK7 intensity contained within the nucleus. Channel 3 Nuclear Intensity Sum, total DNA IF signal contained within the nuclear surface model. Grey fields, multiple nuclei. Nuclear voxels, the number of voxels counted by Imaris within the nuclear surface model. Nuclear volume, volume of the nuclear surface model. # Nuclei, number of individual nuclei identified as present within the model as reported by Imaris. Channel 3 Background, the DNA IF designated as background for the stack and subtracted before DNA quantification. BS Nuclear DNA, calculation of the total DNA present inside the nuclear model, if present. Normal Mode of Gaussian Fit, related directly to tab A. for the same stack file ID, the center of the gaussian peak fit to the DNA intensities of all non-tumor cells segmented in the same image stack. DNA number, the ratio of the total background-subtracted DNA signal inside the nuclear volume to the mode of the total intensities of all the non-tumor controls. Stated another way, how many non-tumor “genomes” worth of DNA signal are measured inside the tumor nuclear volume. Estimated ploidy, twice the number of estimated genomes, assumes two chromosomes per genome equivalent of DNA signal from column AB. Nucleus S:V Ratio, the ratio of the nuclear surface model to the volume contained within that surface is a shape parameter. N:C ratio, the ratio of the nuclear volume to the cytoplasmic volume. Cell S:V, the ratio of cell model surface area to volume contained within that surface is a shape parameter. Cell Area:Nucleus Area, ratio of cell model surface area to nuclear model surface area is relevant to some flux models. Cell volume/ploidy, the ratio of the volume of the cell body model to the estimated ploidy, or an estimate of the amount of cell matter devoted to each theoretical chromosome. Conditional formatting, related to Fig 3, S12 and S13 Figs. Ploidy/Nuclear volume, the number of theoretical chromosomes, or estimated ploidy, per amount of nuclear volume within a nuclear model. Cell or Nucleus prolate:oblate ratio, a parameter we used to estimate the relative degree of elongation versus flattening, related to S15 Fig. Columns AM-AP, explicit counts of sub-, proportional, or supraproportional and near-WT cells, related to Fig 3, S12 and S13 Figs and referenced by tabs D-F. NM, not measured, as in the case of stack 4850T4, where nuclear models were not completed and these cell models are not included in any DNA-based analyses. **C. Normal Cell Data**. Stack ID, the unique file name identifier for normal sample image stacks, stained with proSPC and CK7. Cell model volumes, CV, nuclear model volumes, NV, and ratios of nuclear to cytoplasmic volumes, NC, binned for all patients. Columns F-J, Collected numbers of samples, related to tab E. Columns N-T, summary statistics for all normal distant control cells segmented (see Methods), cited in the main text for mean and SD. **D. Genotype statistics**. Columns A-P, numbers of stacks imaged, depth of imaged stacks segmented, and numbers of cells as well as associated data binned by genotype. Statistics are calculated directly from tabs B and E. Columns Q-X, data by stage, related to Fig 3, S12 and S13 Figs for sub-, proportional, and supraproportional cell classification and quantification. **E. Patient statistics**. Columns A-D., related to columns A-D., tab B. Columns E-J, reporting of imaging and segmentation sampling depth. Columns Q-AB, left-to-right cell formulae walk through the decision rule we used to classify patients according to the degree of proportionality of cell volume to DNA. Columns Q-S, uniform split of cells between three bins, used for Chi-square analysis, column W, relative to columns T- V. Column X, binomial “coin-flipping” test to determine whether, if the distribution from columns T-V was uneven, one or the other category was significantly enriched. Column Y, decision from statistical Chi-square and binomial tests. Abnormal, in this context not like a fusion of wild-type cells. Proportional, more similar to a fusion of wild-type cells. Columns Z and AA, the degree of fold-change for each category Sub- or Supra, relative to the other. Column AB, final classification based on the decision rule, see Methods. **F. Stage Statistics**. Genotype, whether normal, EGFR+, or KRAS+ as indicated. Stage, 0 = normal, 1, 2, 3, binnings of stages, where 1 contains 1A and 1B (related to Tab B). Number of cell models, counts of cell models in each genotype and stage, calculated directly from tabs B and C. Fold change mean TCV/mean NCV, ratio of the mean tumor cell volume to mean normal cell volume for each treatment. Fold change SE, standard error of the fold change, estimated from a Taylor approximation for two variables (see Methods). Columns G- AA, summary statistics, related to Tabs B-C. Columns AN-AU, calculation of relative contributions of each cell category, sub-, proportional, or supraproportional, calculated from Tab B (see formulas). **G., Model statistics and fitting**. This tab contains summary statistics as reported by Igor Pro software for each parameter, treatment, and ploidy type (all cells are all ploidies, euploids only = 1.6–4.4n estimated ploidy), and relevant to quantitation derived from data in Tabs B-C and from Imaris software outputs. Columns A-D, parameter of interest, treatments, and relevant species. # of models, the number of models included in the analysis. Max, the maximum value of the parameter in the analysis. IQR, the interquartile range for the data used in the analysis. Bin width, specified bin width for a histogram of data, based on the Freedman-Diaconis rule. Number of bins, the number of histogram bins used for plotting, derived from column H, and also the sample size used for distribution fitting. Columns J-L, summary statistics for datasets reported by Igor Pro. Columns M-AA, details on initial guesses, fitting function attempted, rationale for choice of best fit (column T), fitting function chosen (column S and green fields are relevant), and best-fit parameters for the chosen fit with their reported errors (columns U-Z). See also, Methods for the form of the fitting function. Cells V56-W57 show the source of the normal 2n peak values used for comparison to tumor cells in the main text, e.g., 582 is the center of the first gaussian from the mixture model used to fit normal AT2 cell data, cell V56, and 179 was the width1 parameter, which is $\sqrt{2}\sigma$. **H. Growth phenotypes observed**. Explicit quantitation of the frequency with which segmented stacks contained the specified growth patterns described in Fig 4 and the main text. Related to Tab B.r. **I. CK7 in cells > 2 pL**. Cited in the main text, and relevant to the drop in high-intensity CK7 staining seen in very large tumor cells, related directly to S25 Fig. Columns A-B, data copied from tab B for all rows containing cell and nuclear models. Columns C-F, counts of the respective categories of cells with greater than or less than half-max cytoplasmic CK7 intensity (127.5 = 1/2 x 255 8-bit units, see formulas), or cells with greater than or less than 2 pL volume (2000 fL = 2000 μm^3^). Columns H-N, quantitation of the percentages reflected in each region of S25 Fig and cited in the main text.(XLSX)

S1 Video Sheet growth model. A high-coverage, representative 3D structural model displaying sheet growth, shown several times in 2D z-projections or slice views and from EGFR+ patient 4843 (Figs 1 and 4, S1 and S2 Figs). In the first part, cell bodies are shown as a rainbow depiction, and “stromal” (non-CK7-staining) controls as white stone. In each image stack, these white-stone colored nuclei are internal controls used to estimate the DNA IF signal associated with 2n wild-type cells (related to S1 Data, tab A). In the second part of the video, cell bodies are shown translucent white and nuclear surface models are depicted as a rainbow.
(MP4)

S2 Video Solid growth model (A-B). A representative 3D structural model of solid growth in EGFR+ patient tumor 4840. A., cell bodies are shown in a rainbow depiction, and non-tumor intra-stackcontrols for wild-type DNA are shown in white stone. Observed in the video are several examples of non-tumor control nuclei which are closely associated with cell body model surfaces. In the second part of the video, cell bodies are shown as translucent white and nuclear models are shown in rainbow coloration. In this model nuclei often seem to fill up much of the space inside the cells B., demonstration of rendering of nuclear envelopes, rainbow, based on Lamin A+C staining (green). Coverage of modeling for this stain, which was of excellent quality, is representative. Lamin A+C-based envelopes are slightly smaller than nuclei, which is why the stain can be seen on top of the rendered surfaces (see S4 Fig and Methods).
(ZIP)

S3 Video Sparse growth model. A representative 3D structural model of sparse growth from EGFR+ patient 3437. In most of the video cell bodies are shown in rainbow. In the last part of the video, cell bodies are translucent white and nuclei are depicted as a rainbow. Note that cells and nuclei appear more rounded in this model.
(MP4)

S4 Video **(A, B)**, transition from lesions/neoplasms to sheet growth. Another structural model from EGFR+ patient 4843 of what we referred to as a “transition zone”, with proSPC-presenting AT2-like cells, presumed to be neoplasms, directly adjacent to sheet-like growth of enlarged CK7-overexpressing cells (Fig 4). Cells in sheets like these take on a “dripping” or “melting wax” appearance suggestive of shedding cells with size dysregulation. The enlarged cells show loss of AT2-like differentiation in their proSPC staining. Presumptive neoplasms have both sparse proSPC puncta, unlike AT2 cells, and also negligible CK7 staining. In this way we delineated neoplasms simply by the property that they can never pass curation as models using methods we used for either normal AT2 cells, or for tumor cells. A., Cell bodies shown in rainbow, with proSPC staining introduced in the green channel during the last half of the video to illustrate the full transition of the pathology from more AT2-like neoplasms to presentation of proSPC only at the joints of large shedding cells. Note that regions of the tumor cell model possess proSPC process-like staining, but no evidence of neoplasms from which they are connected to neighbors, suggesting the process-like staining at cell junctions between tumor cells is a connection between tumor cells. B., cell bodies shown in white translucent, and nuclei shown in rainbow, with abundant stromal controls in white stone. Flybys show long fiber structures and elongated stromal nuclei of unknown type that we commonly observed in LA (yellow circle, with long pause) proSPC (green), CK7 (red).
(ZIP)

S5 Video Sheet growth in a gland-like formation. 3D structural model from KRAS+ patient 4855 of the majority of a gland-like formation, with cell bodies shown as a rainbow. Also shown in the latter part of the video in proSPC channel 1 (green) and DNA channel 3, (cyan) are a handful of embedded proSPC-presenting neoplasms. This cell type is not segmentable by using either the normal or tumor method (see also S4 Video). The overall topology is reminiscent of an alveolus, which is a cavity surrounded by CK7-presenting cells, and which develop from proSPC presenting cells. However, in this case no flattening of tumor cells is observed and sparsely proSPC-presenting cells cannot be modeled in 3D. The video ends on a frame that highlights the appearance of these "cryptic” neoplasms (large yellow arrows).
(MP4)

S6 Video Processes act like hairlike projections from AT2 cells during intermediate stages of digestion. Gentle digestion with a collagenase/elastase/hyaluronidase cocktail at room temperature was employed upon an IF-treated FFPE specimen to test whether processes, like cells, were mechanically and biochemically separable from extracellular material. Shown in the video is a partial fracture of a tissue section during the gentle digest of ECM. As the fragment drifts away from the camera due to convection, stumps of fragments are seen rapidly waving but remain attached to cell bodies of still-embedded AT2 cells. proSPC (green), DNA (cyan). Normal lung shown. Scale bar, 2 μm.
(MP4)

S7 Video Processes are extremely fragile, but are biochemically and mechanically separable from lung tissue. proSPC (green) staining process fragment isolated from an IF-treated FFPE tissue specimen during gentle digest of ECM of normal lung using collagenase/elastase/hyaluronidase cocktail at room temperature. Scale bar, 30 μm.
(MP4)

S8 Video Processes which are biochemically and mechanically separable from ECM remain linked between neighboring AT2 cells. Another intermediate stage in digestion of IF-treated FFPE lung with a collagenase/elastase/hyaluronidase cocktail at room temperature. proSPC (green) staining AT2 process linking two cells is freed from extracellular material, but partway through the video can be seen swaying independently of the tissue and the AT2 cells due to convection. Normal lung shown. Scale bar, 2 μm.
(MP4)

S9 Video Processes which are biochemically and mechanically separable from ECM remain linked between neighboring AT2 cells. proSPC (green) staining AT2 process shown linking two AT2 cells is freed from extracellular material butcontinues to sway due to convection, during gentle digest of ECM of normal lung with collagenase/elastase/hyaluronidase cocktail at room temperature. DNA (cyan). Scale bar, 2 μm.
(MP4)

S10 Video Processes are fragile, but retain some mechanical integrity while remaining attached to tissue at one end. Brightfield image showing a broken hairlike process (yellow arrow) that is still stiffly moving due to thermal convection, during gentle digest of ECM of an FFPE specimen of normal lung at room temperature with a collagenase/elastase/hyaluronidase cocktail. Scale bar, 5 μm.
(MP4)

S11 Video proSPC and CK7 both separate from ECM in a hairlike projection that is part of the same structure. Two-color IF image of the same broken proSPC-staining process shown in S10 Video. proSPC (green) is wrapped in intracellular CK7(red). Scale bar, 2 μm.
(MP4)

S12 Video Autofluorescence of ECM is distinct from processes that stain with both proSPC and CK7. Three-color 3D projection based on a spinning-disk confocal image stack of an intermediate state of gentle digest of ECM for normal lung FFPE with collagenase/elastase/hyaluronidase at room temperature. ECM strands are visible as bundles of autofluorescence (blue arrow during pause)in the DNA channel (cyan) and take a divergent path from the process shown (white arrow during pause), which remains connected between two neighboring cells and stains with both markers, CK7(red), proSPC (green). IF highlights the string-of-beads appearance of processes that was visible with the spinning disk confocal under conditions of the digest. Scale bar, appropriate for region closest to viewer in this perspective, is 5 μm.
(MP4)

S13 Video Process-like staining is visible at intracellular junctions inside clumps of tumor cells. Three-color 3D projection of an intermediate state of gentle digest of ECM for tumor lung with collagenase/elastase/hyaluronidase cocktail.CK7 (red), proSPC(green), and DNA (cyan) illustrate that there is enrichment of proSPC at the intracellular junctions inside of this digest-resistant clump of tumor cells.
(MP4)
