# Supplementary figures and images for: Epithelial cell size dysregulation in human lung adenocarcinoma
Source: PLoS One. 2022 Oct 6;17(10):e0274091. doi: 10.1371/journal.pone.0274091 (PMC9536599; doi:10.1371/journal.pone.0274091)

Figure S1

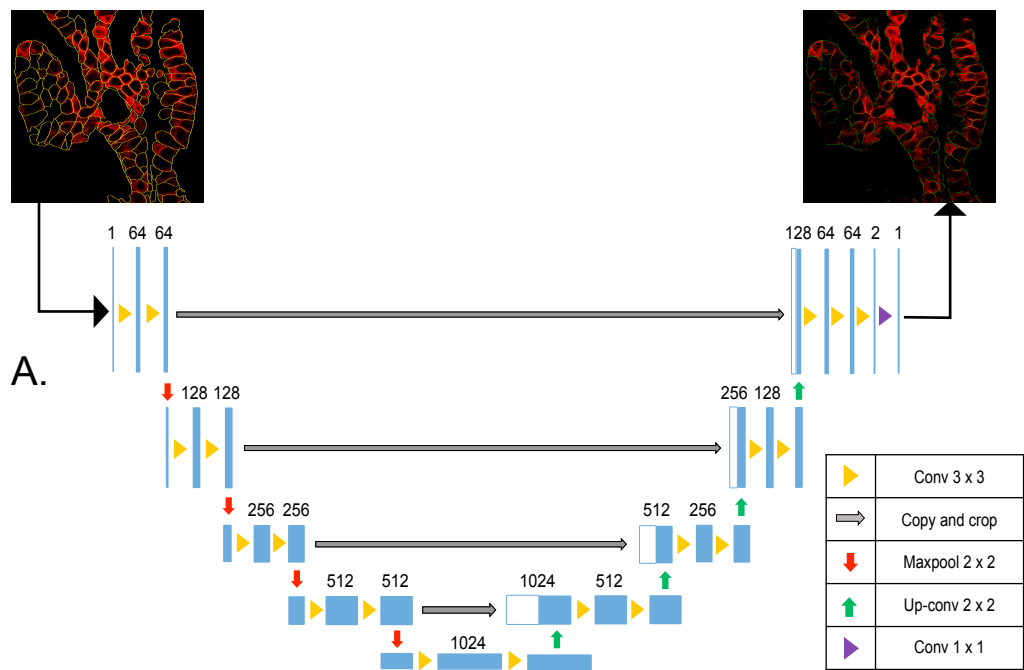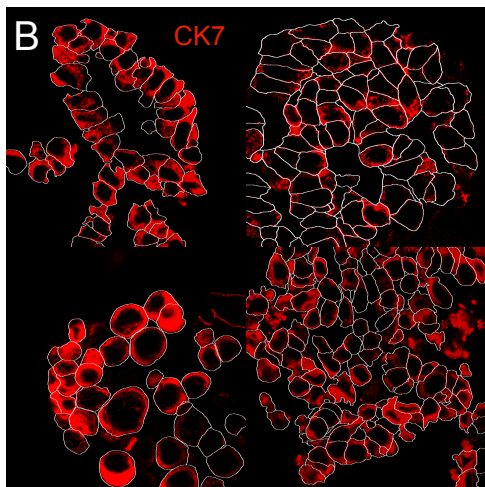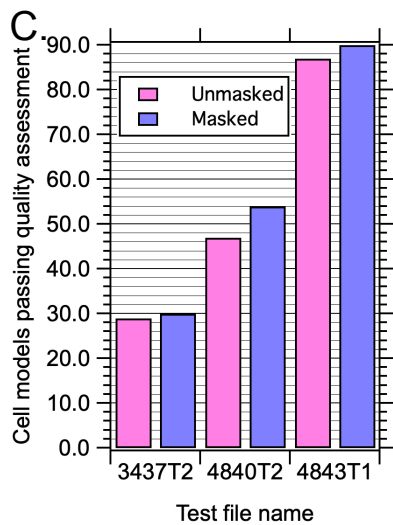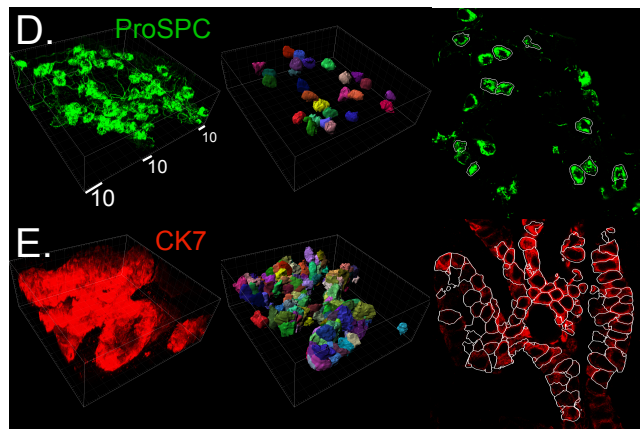

Supplement: S1 Fig — A., Our machine learning approach employed a UNet algorithm, trained with manual annotations of cell boundaries. Both inputs and outputs of the algorithm are 2D pixel arrays, which involve a downsampling leg to conserve computational resources (left side of “U”) and an upsampling leg to recover positional information (right side of “U” and grey arrows). Data in a region local to a pixel of interest is used to generate a confidence score for that pixel that describes the likelihood that a cell boundary would have been drawn by the human annotator. This 2D array of confidence scores is the output for each of the ~250–500 images per stack, and represents a simulated cell boundary channel. B., representative manual annotations of cell boundaries, used to train the neural network. C., data showing marginal improvement of cell coverage which resulted by masking data outside of the annotation region. Cell models, y-axis, varied with cell density in stacks, x-axis. D. AT2 cell (proSPC) raw staining, left, segmented using Imaris to reveal individual cell surfaces, middle, which accurately predict cell boundary outlines (white), shown in 2D slice right. E. CK7 stain, red, left, after machine learning and segmentation of cell surfaces, middle, correctly predict cell boundaries of tumor cells (white); shown in 2D slice, right, and are comparable in accuracy to Imaris segmentation shown in D. (PDF) [file pone.0274091.s001.pdf]

Figure S2

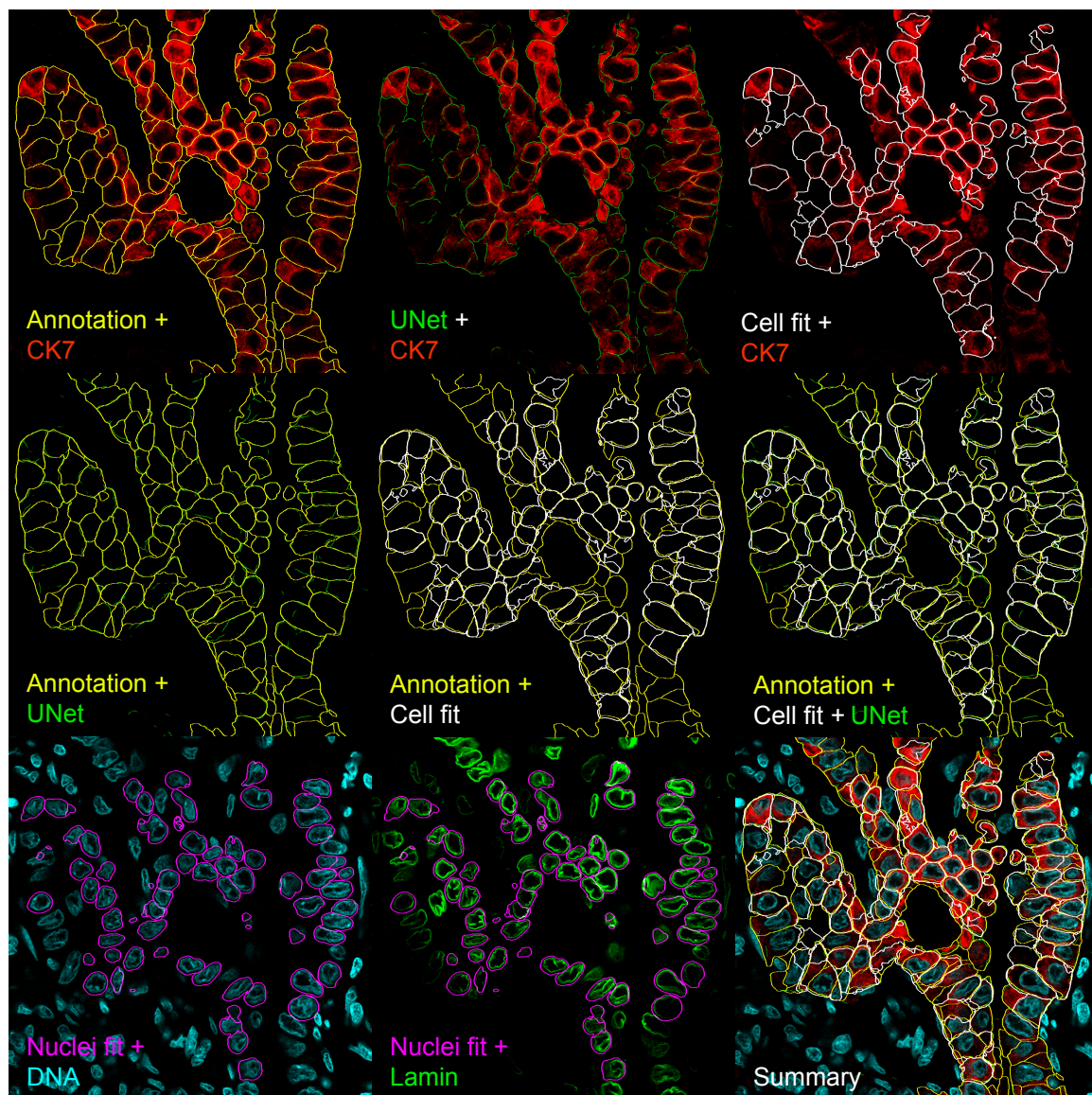

Supplement: S2 Fig — Cells passing manual curation display agreement between manual annotation, UNet prediction, Imaris 3D surface fit, and IF data. Yellow, annotation. White, Imaris fit to cell bodies. green lines, UNet predictions. Magenta, nucleus fit. Green data, Lamin A+C. Cyan data, DNA. Red data, CK7. Related to Figs 1D and 4A, left, S1 Fig, top, and S1 Video. (PDF) [file pone.0274091.s002.pdf]

Figure S4

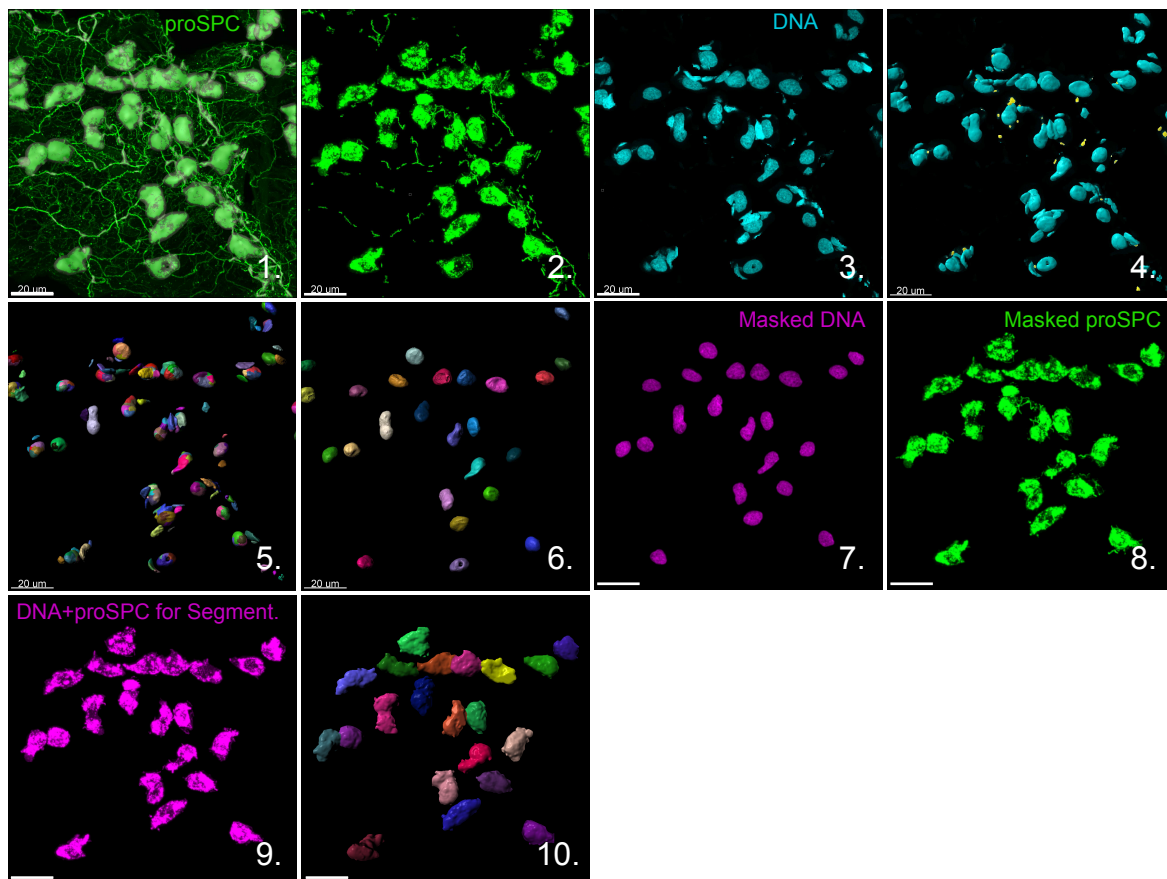

Supplement: S4 Fig — 1. A coarse mask, translucent surfaces, is generated using a smoothed isosurface of proSPC data, green. 2., masked proSPC data contains some small process fragments which are further masked away. 3. DNA, cyan, is masked with the coarse proSPC model and contains inappropriate DNA clippings from abundant stromal cells. 4. a clipping model is generated, and clippings retained so that they can be deleted from the DNA channel. 5. over-split surfaces are joined using the unify tool. 6. joined DNA isosurfaces created using the unify tool in Imaris. 7., masked normal AT2 DNA, magenta, is added to 8., masked proSPC, to create a segmentation channel, 9. 10., 9. is used to generate the final cell body model using an isosurface guided by eye using the Imaris wizard. Scale, 20 μm. (PDF) [file pone.0274091.s004.pdf]

Figure S5

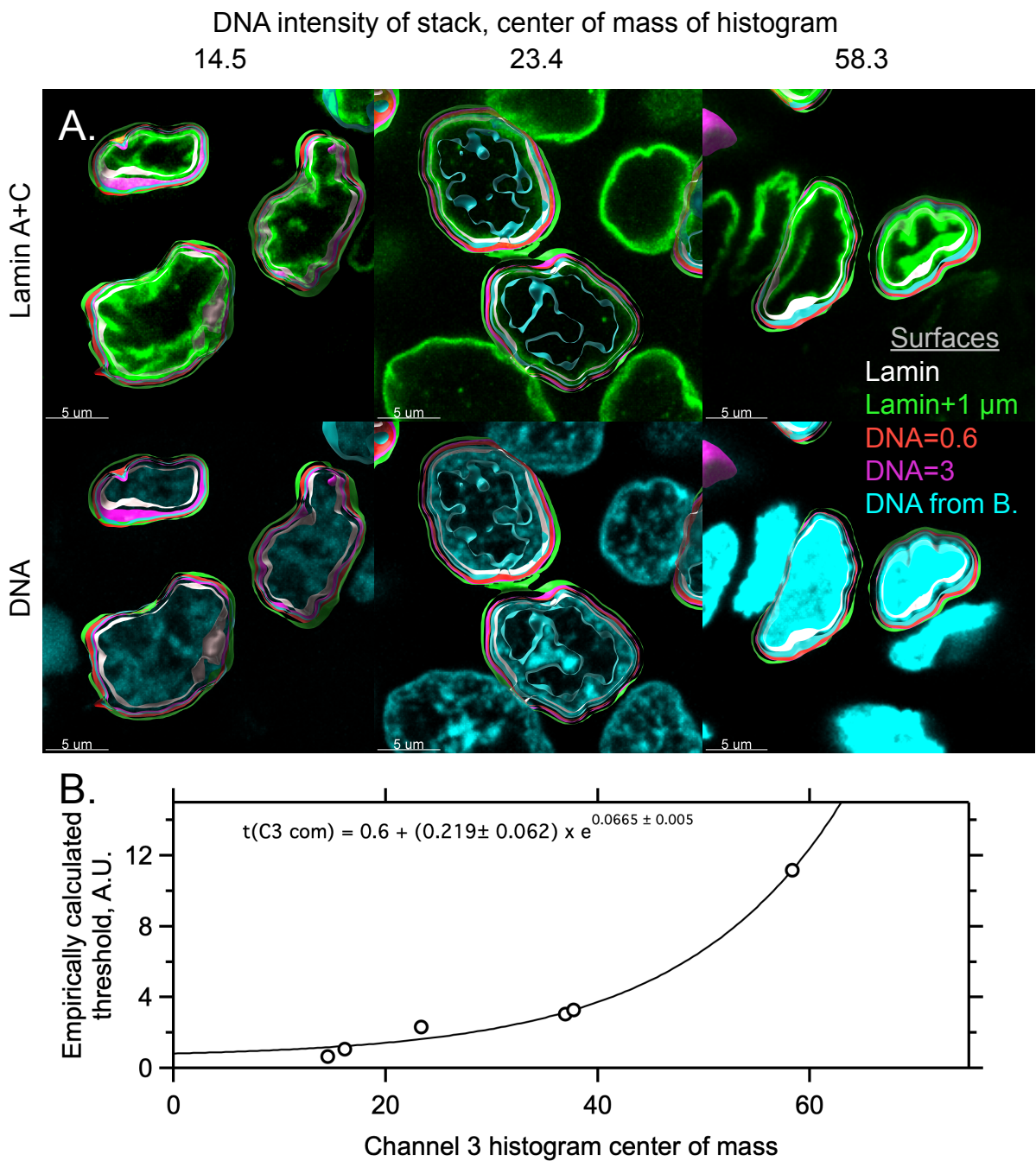

Supplement: S5 Fig — For a DNA isovolume-based 3D surface, the observed volume is a function of the threshold selected (e.g., 0.6, 3, shown) and the brightness of the DNA. A., 1 μm projections associated with files containing three levels of DNA intensity. Surfaces, isovolumes of DNA with thresholds 0.6, red and 3, magenta, Lamin A+C model, white and 1 μm dilation of same, green. Cyan surface shows thethreshold selected, based on B. B., empirical fit to six files, see methods, Eq 14. (PDF) [file pone.0274091.s005.pdf]

Figure S6

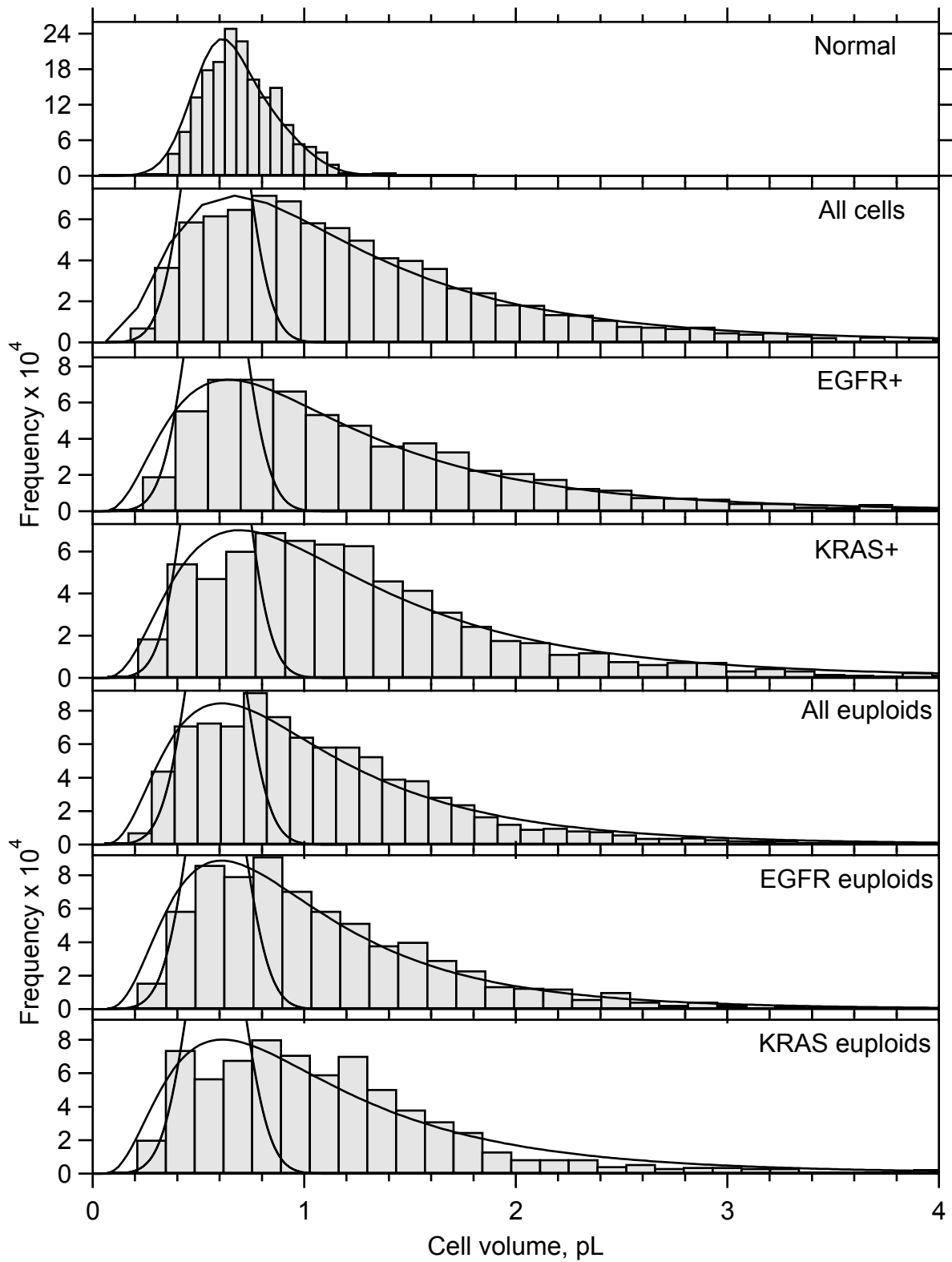

Supplement: S6 Fig — Top, Normal AT2 cells (n = 802). All cells, N = 21, n = 4082. EGFR+, all ploidies, N = 10, n = 2194. KRAS+, all ploidies, N = 11, n = 1939. All euploids, cells with estimated ploidies of from 1.6–4.4n (n = 2683). EGFR euploids, n = 1411. KRAS euploids, n = 1260. Fits are a Gaussian mixture model, top, and lognormal, other histograms. Small Gaussians overlaid are the first Gaussian from the mixture model fit to normal AT2 cells, corresponding to the 2n population. Goodness-of-fit statistics available in S1 Data, tab G. (PDF) [file pone.0274091.s006.pdf]

Figure S7

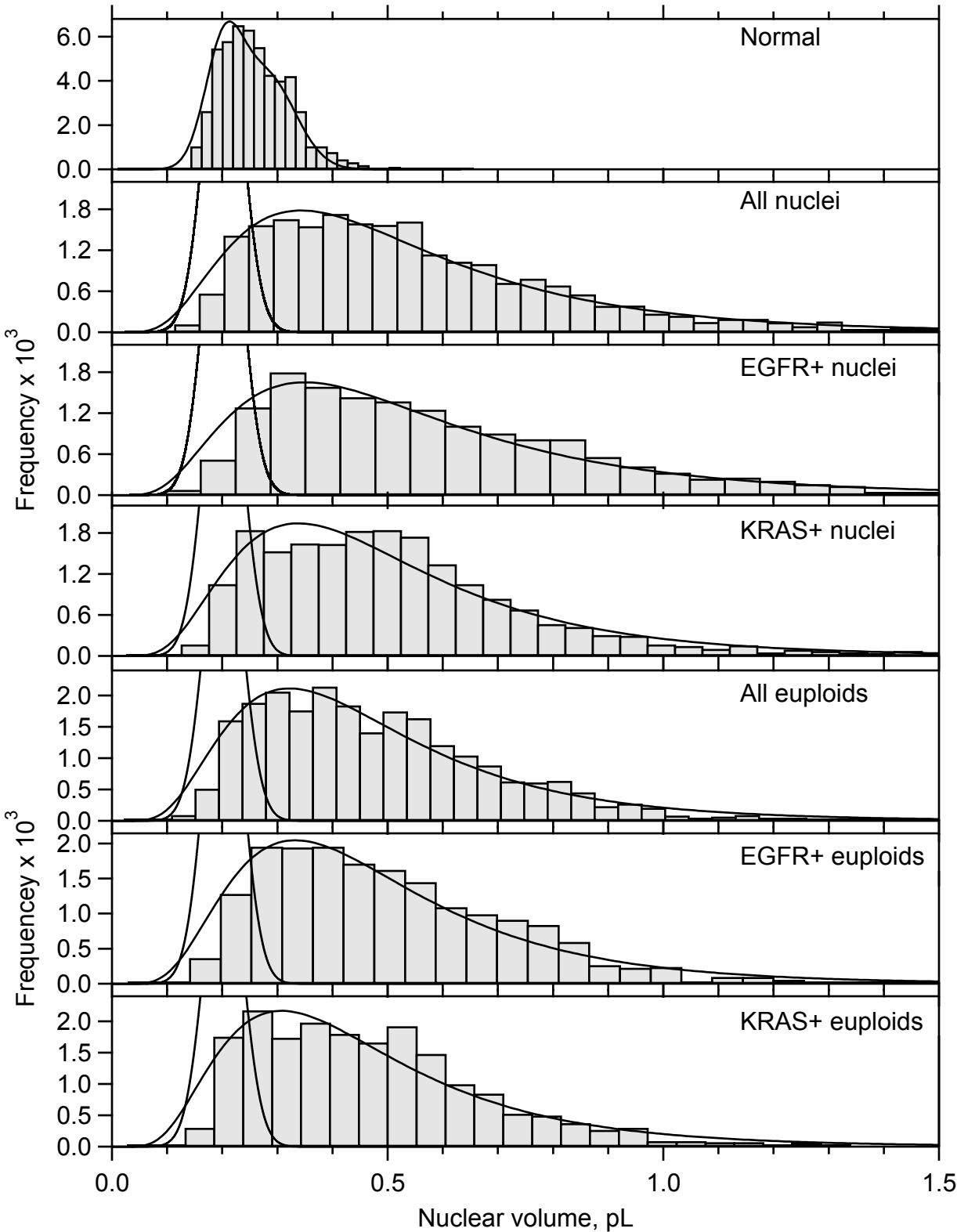

Supplement: S7 Fig — Top, Normal AT2 cells (n = 802). All nuclei, N = 21, n = 4082. EGFR+, all ploidies, N = 10, n = 2194. KRAS+, all ploidies, N = 11, n = 1939. Euploids, nuclei with estimated ploidies of from 1.6–4.4n (n = 2671). EGFR euploids, n = 1411. KRAS euploids, n = 1260. Fits are a Gaussian mixture model, top, and lognormal, other histograms. Small Gaussians overlaid are the first Gaussian from the mixture model fit to normal AT2 nuclei, corresponding to the 2n population. Goodness-of-fit statistics available in S1 Data, tab G. (PDF) [file pone.0274091.s007.pdf]

Figure S8

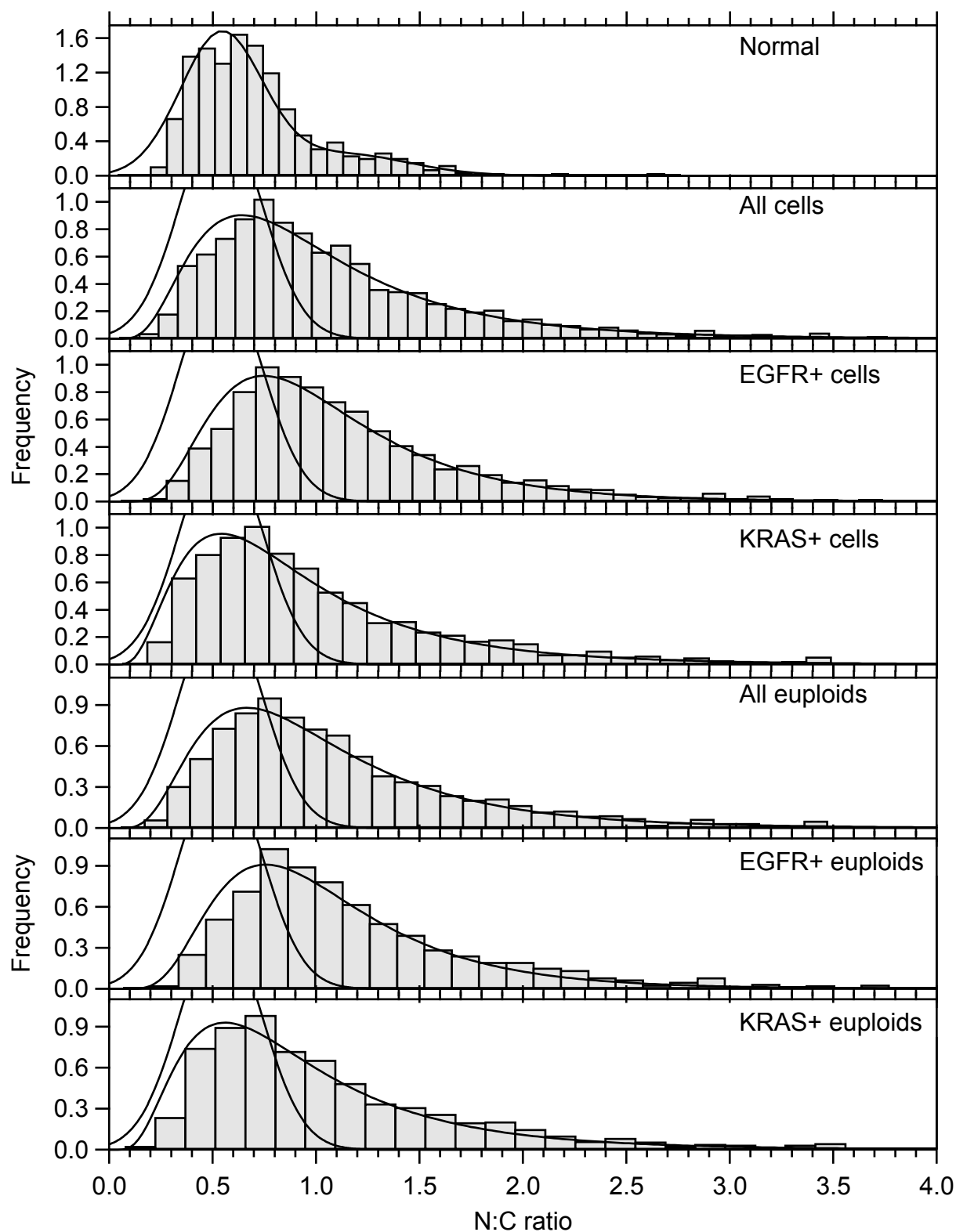

Supplement: S8 Fig — Top, Normal AT2 cells (n = 802). All nuclei, N = 21, n = 4078. EGFR+, all ploidies, N = 10, n = 2194. KRAS+, all ploidies, N = 11, n = 1884. Euploids, N:C ratios from cells with estimated ploidies of from 1.6–4.4n (n = 2668). EGFR euploids, n = 1411. KRAS euploids, n = 1257. Fits are a Gaussian mixture model, top, and lognormal, other histograms. Small Gaussians overlaid are the first Gaussian from the mixture model fit to normal AT2 cells, corresponding to the 2n population. Goodness-of-fit statistics available in S1 Data, tab G. (PDF) [file pone.0274091.s008.pdf]

Figure S9

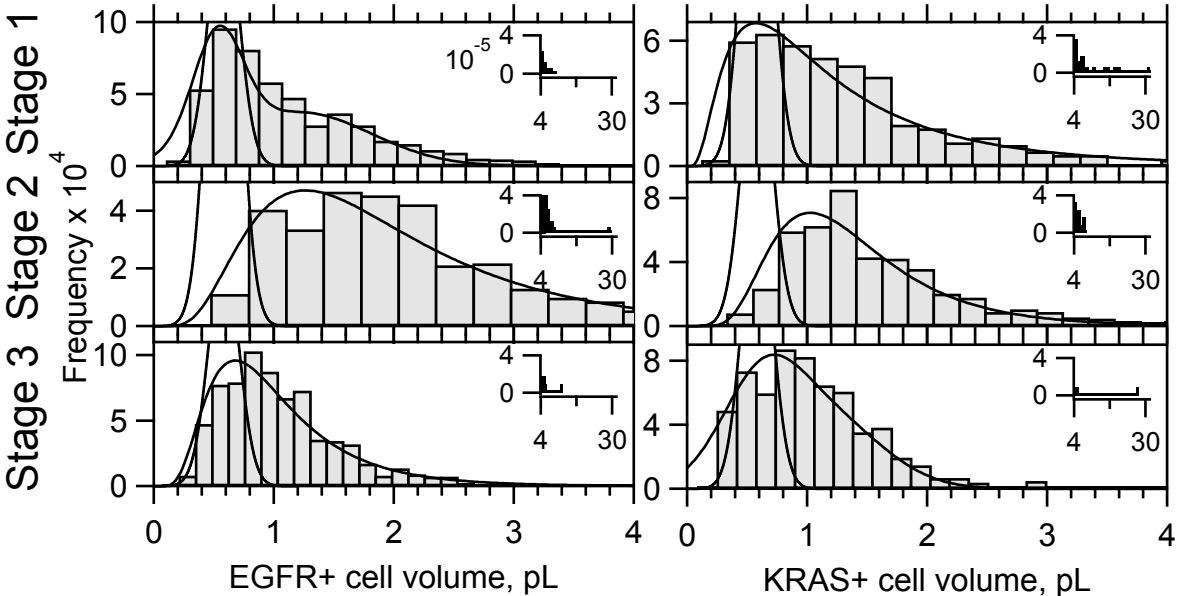

Figure S10. Polyploid cell volumes by stage and genotype.

Supplement: S9 Fig — Related to Fig 2B, histograms of cell volumes for all segmented cells regardless of ploidy (N = 21, n = 4082). Fits, upper left, Gaussian mixture model, other fits, lognormal. Small Gaussian fits, first 2n population from a mixture-model fit to normal AT2 cells. Goodness-of-fit statistics and parameters from regression fit to normal AT2 cells as well as sample sizes by stage and genotype available in S1 Data, tab G. (PDF) [file pone.0274091.s009.pdf]

Figure S10

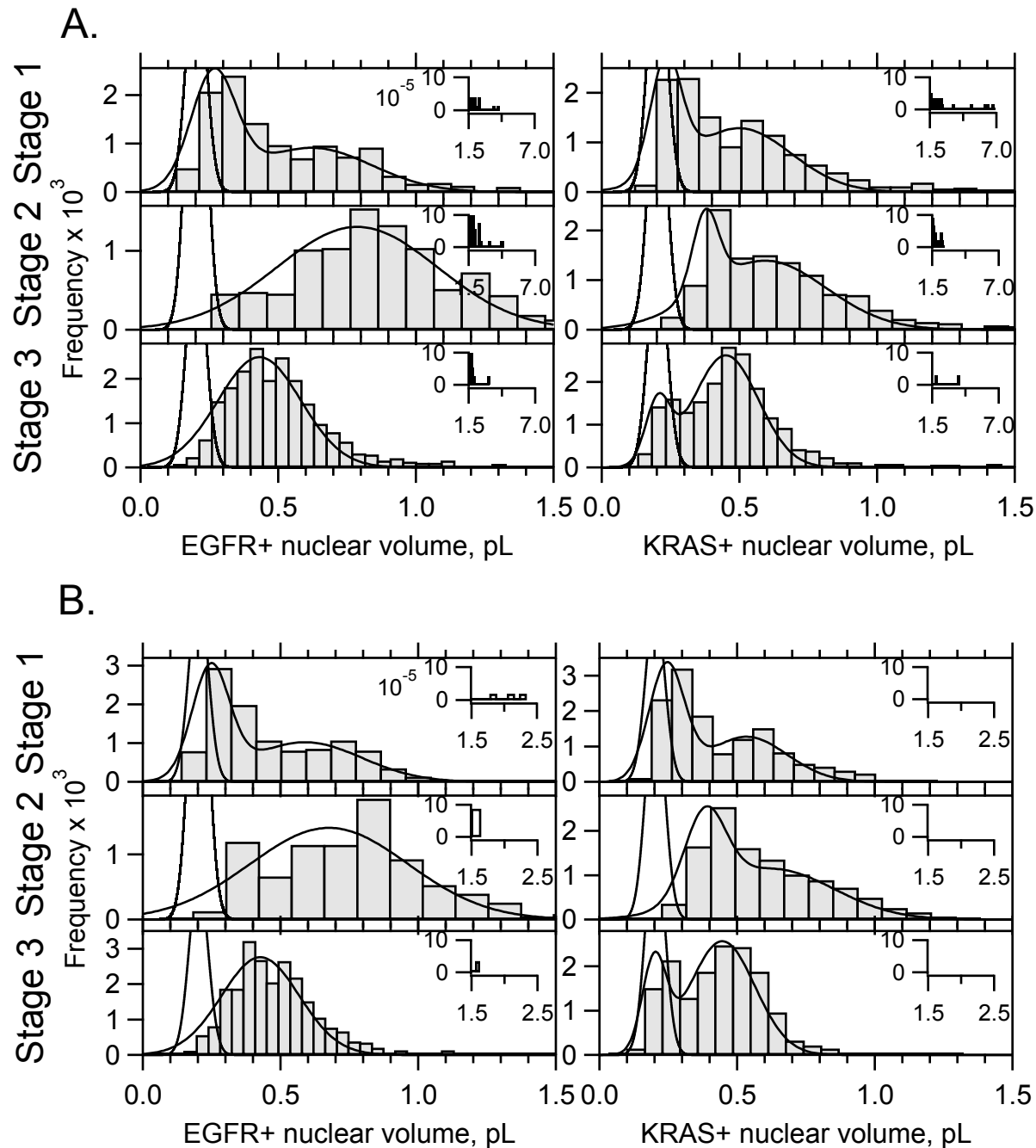

Supplement: S10 Fig — A., Polyploid (n = 4082) and B., euploid (n = 2671) nuclear volumes by stage and genotype. Small Gaussian fits, first 2n population from a mixture-model fit to normal AT2 nuclei. Fits with two apparent modes, two-gaussian mixture models, other fits, single gaussians. Goodness-of-fit statistics and parameters from regression fit to normal AT2 cells as well as sample sizes by stage and genotype available in S1 Data, tab G. (PDF) [file pone.0274091.s010.pdf]

Figure S11

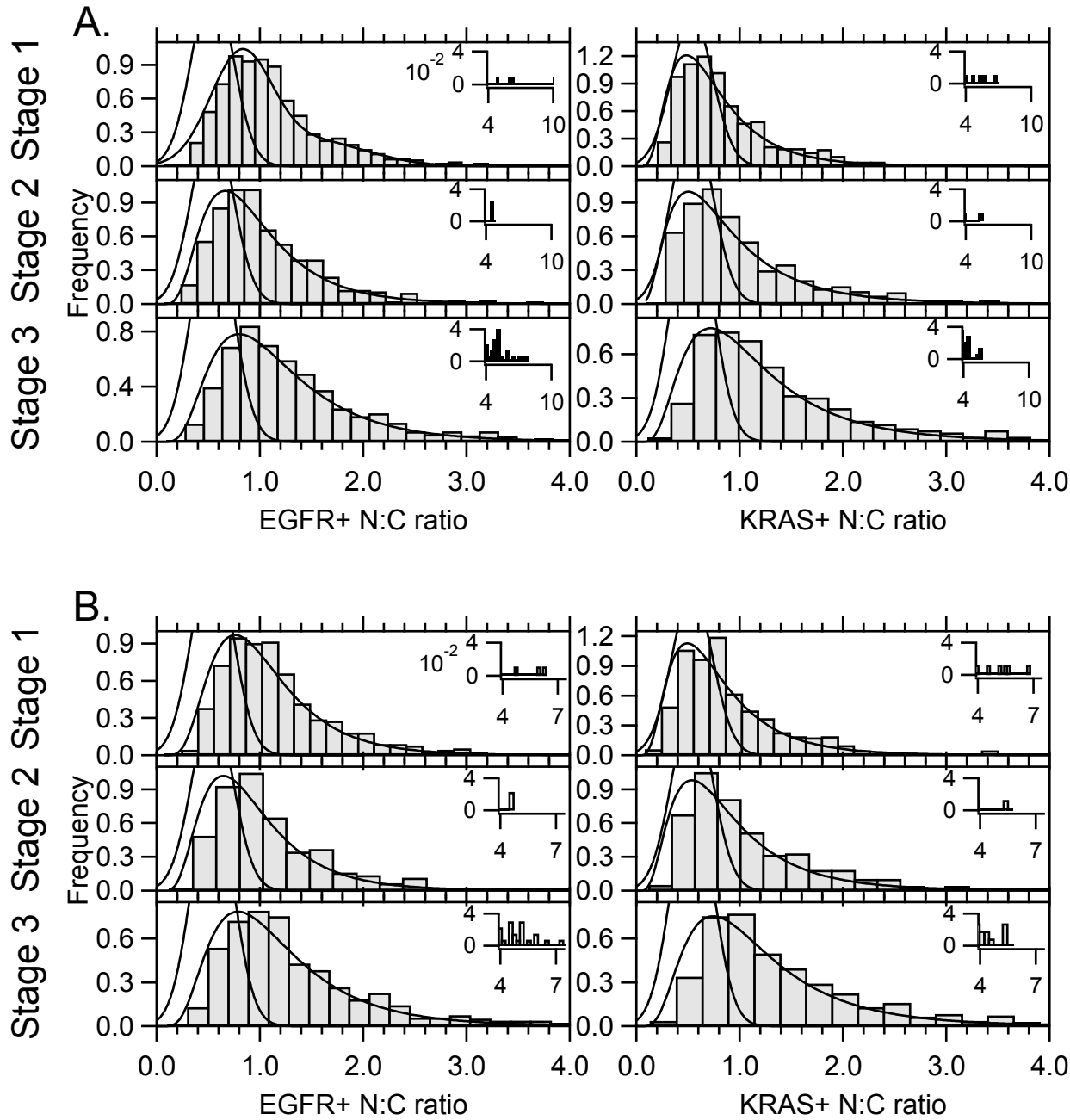

Supplement: S11 Fig — A., Polyploid and B., euploid N:C ratios by stage and genotype. Small Gaussian fits, first 2n population from a mixture-model fit to normal AT2 cell nuclei and cell bodies. Fits, top row, two Gaussian mixture, other fits, lognormal. Goodness-of-fit statistics and parameters from regression fit to normal AT2 cells as well as sample sizes by stage and genotype available in S1 Data, tab G. (PDF) [file pone.0274091.s011.pdf]

Figure S12

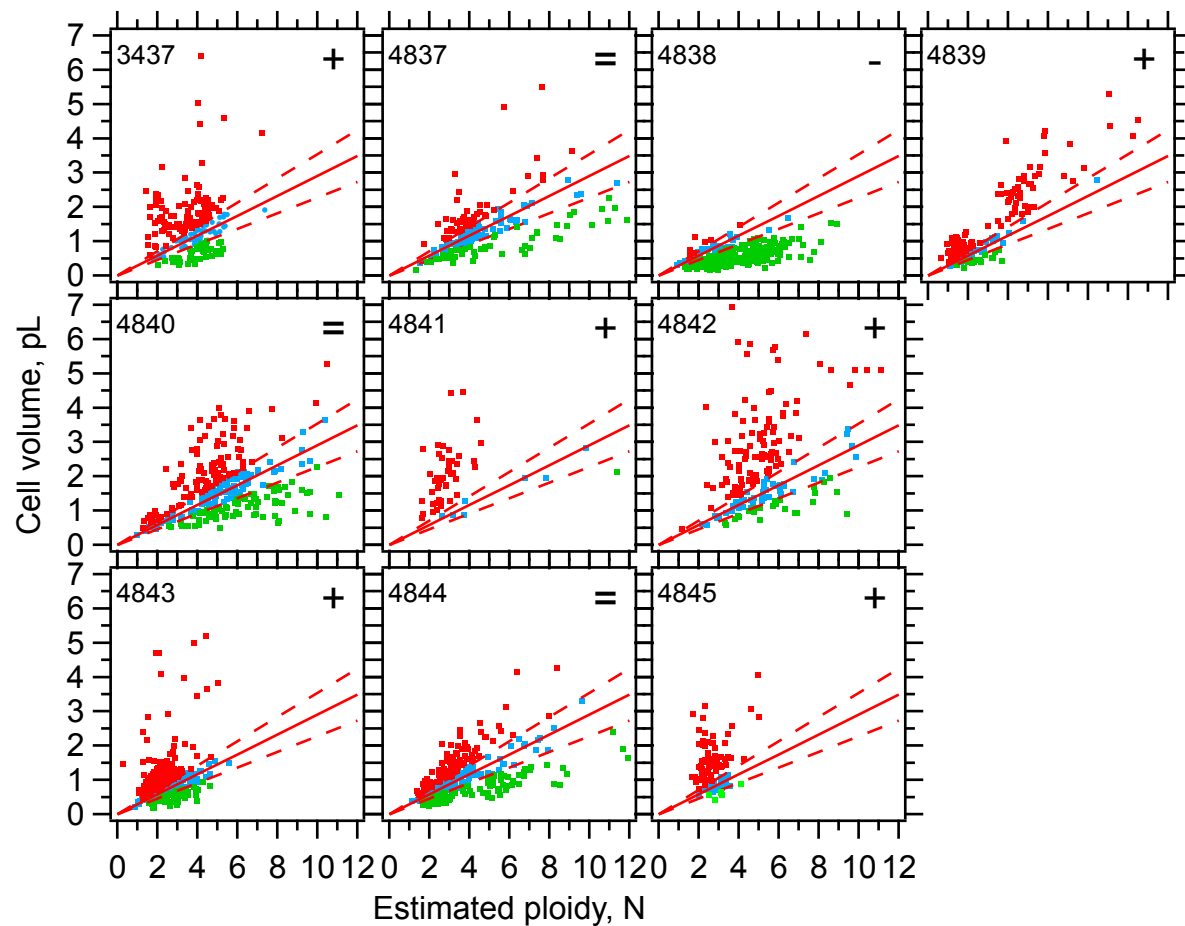

Supplement: S12 Fig — "-", " = ", "+", classifications based on cells with sub-, green, proportional, blue, or supraproportional, red, behavior, used to construct Fig 3B. See Methods and S1 Data tab E for classification regime. Each dot represents a single cell measurement (n = 4082, related to S1 Data, tab B). Rows, stages 1–3. Some outliers are excluded from the edges of the plot for clarity and their data can be found in S1 Data, tab B. (PDF) [file pone.0274091.s012.pdf]

Figure S13

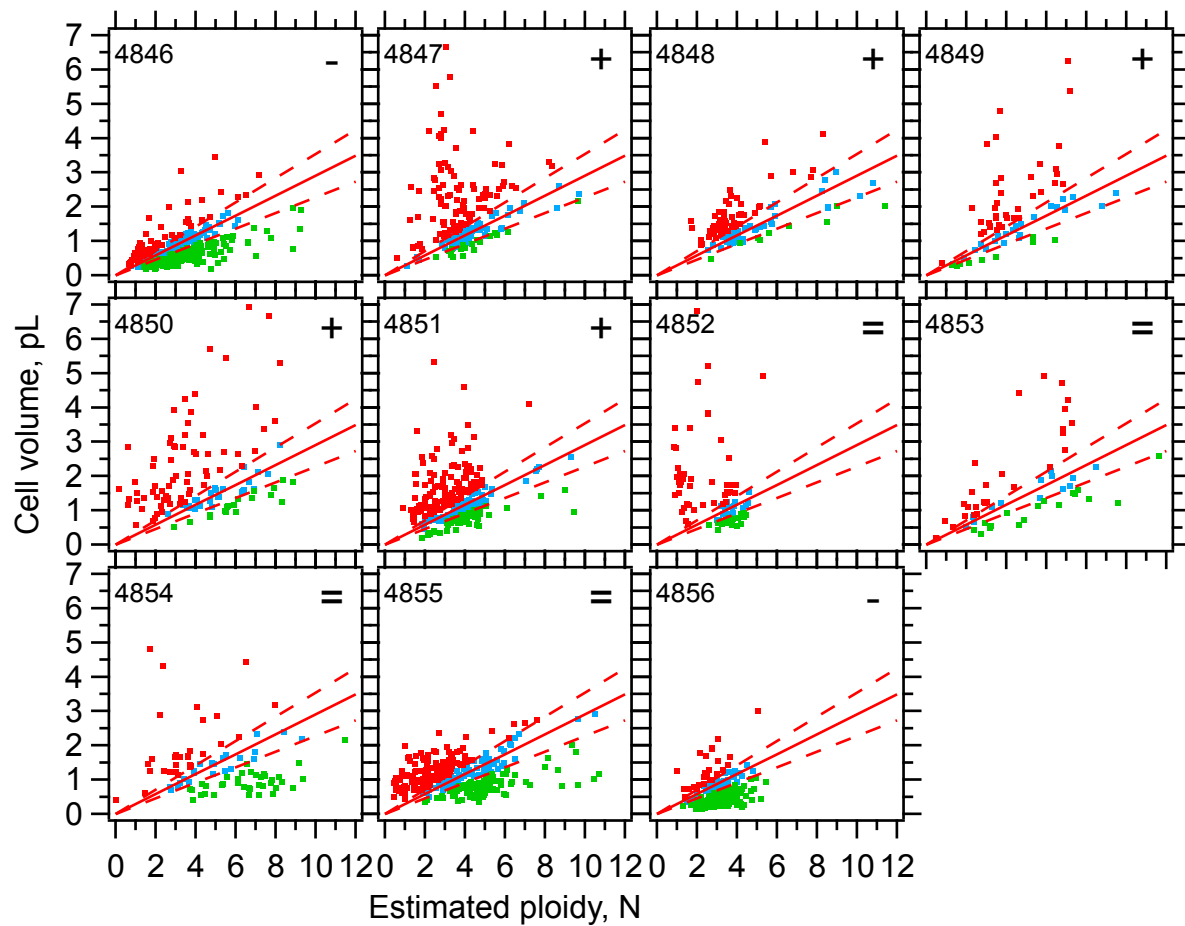

Supplement: S13 Fig — "-", " = ", "+", classifications based on cells with sub-, green, proportional, blue, or supraproportional, red, behavior, used to construct Fig 3B. See Methods and S1 Data tab E for classification regime. Each dot represents a single cell measurement (n = 4082, related to S1 Data, tab B). Rows, stages 1–3. Some outliers are excluded from the edges of the plot for clarity and their data can be found in S1 Data, tab B. (PDF) [file pone.0274091.s013.pdf]

Figure S14

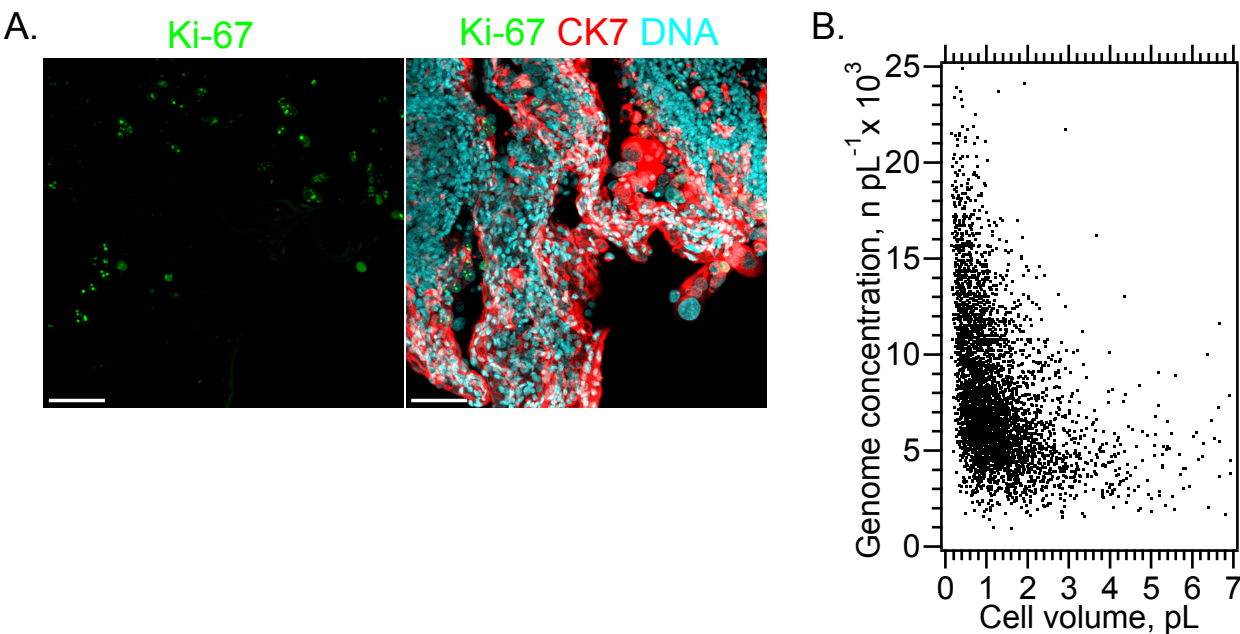

Supplement: S14 Fig — A., Ki-67 proliferation marker is observed in cells across the range of volumes for LA. Green, Ki67, with merge to CK7, red and DNA, cyan at right. Quantitation of nuclear size data in proliferating cells is shown in Fig 7A, and related to S23 and S24 Figs. B, Estimated ploidy per unit volume within the nucleus, or genome concentration, plotted as a function of cell volume shows that nearly all cells with a volume greater than 2 pL display diluted genomes. n = 4082, related to S1 Data, tab B. (PDF) [file pone.0274091.s014.pdf]

Figure S15

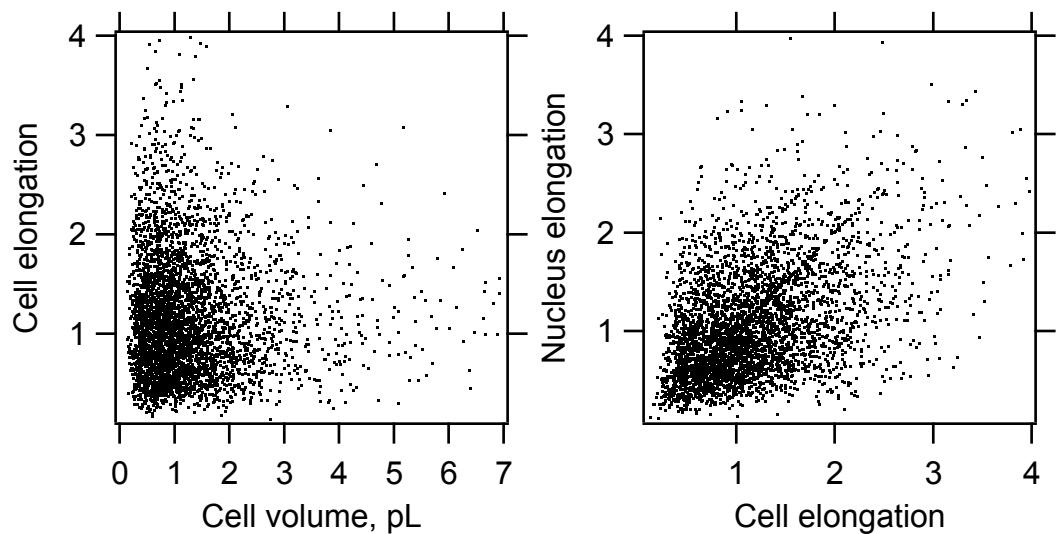

Supplement: S15 Fig — Left, plot of cell elongation, defined as the ratio of the prolate ellipticity to the oblate ellipticity, as a function of cell volume suggests smaller cells are more elongated, as they are observed in sheet growth (Fig 4 and S1 Video, and related to Fig 9B). Right, cell and nuclear elongation are weakly positively correlated (R2 = 0.21). (PDF) [file pone.0274091.s015.pdf]

Figure S16

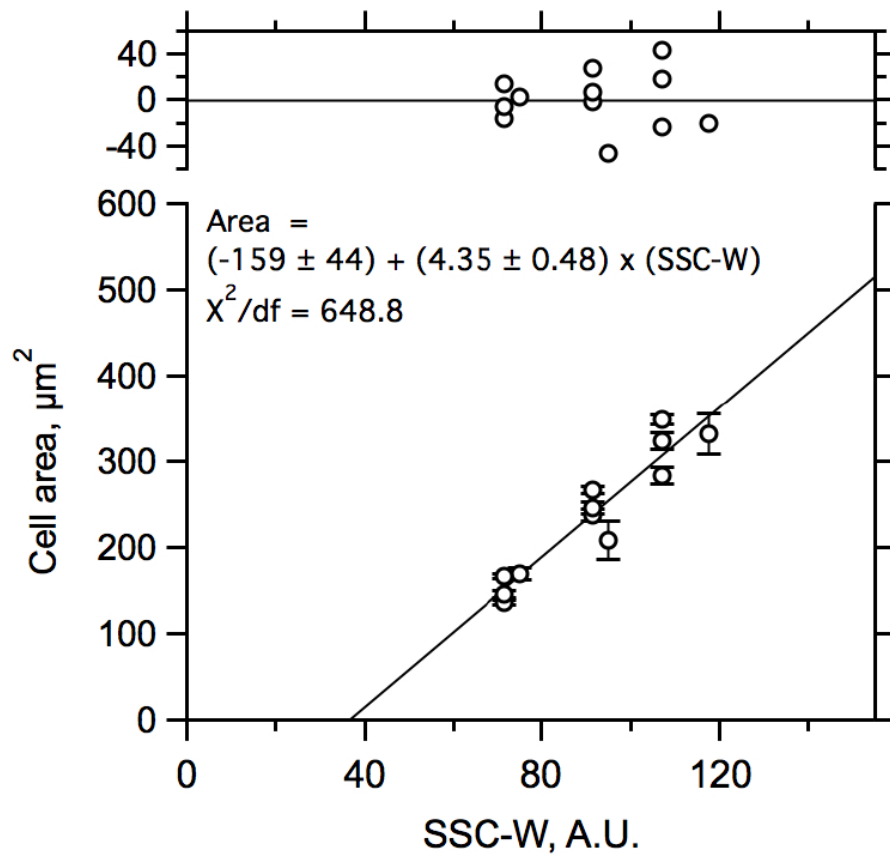

Supplement: S16 Fig — Y-axis, the mean cell area collected from a SSC-W gate. X-axis, the midpoint of the size gate used to sort cells. For reference, an area of 100 μm2 is equivalent to a spherical volume of 0.75 pL, and 400 μm2 is equivalent to a spherical cell of ~6 pL Note also that the x-intercept is near the lower limit of detectable SSC-W. Fit, linear regression with fit parameters and errors as shown. All data is for near-euploids as described in Fig 5. N = 4 patients, with three size gates each. Error bars, SEM. (PDF) [file pone.0274091.s016.pdf]

Figure S17

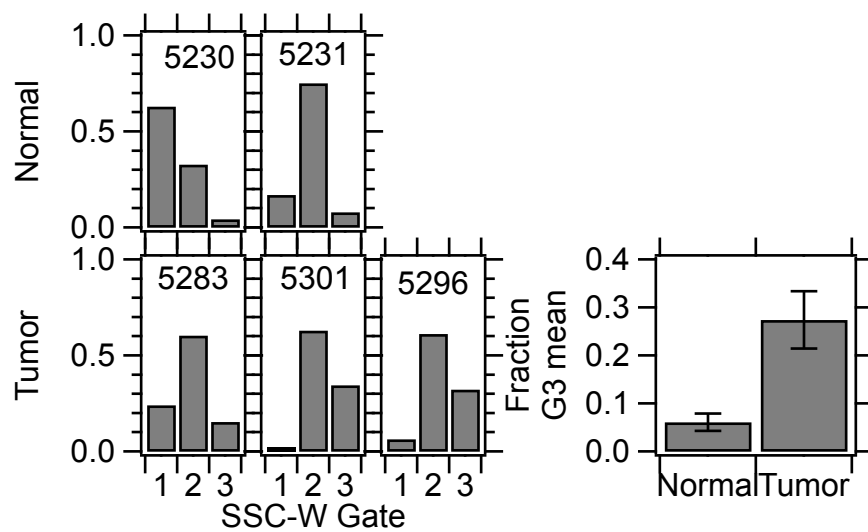

Supplement: S17 Fig — Related to Fig 5. CD45-/Zombie Red-/EPCAM+/Near-euploid cells gently dissociated from normal, top, or tumor, bottom surgical resections were further split according to side-scattering width (SSC-W). SSC-W gates: 1 = 64–79, 2 = 84–99, 3 = 101–113. Bars represent the fraction of all cells in gates G1-G4 for SSC-W vs SSC-H (See Fig 5B). Gate G4 for SSC-W is not shown, and contains sparing aggregates and debris. Right plot, mean of all fractions found in G3, containing the largest singlet cells. Error bars, SEM. (PDF) [file pone.0274091.s017.pdf]

Figure S18

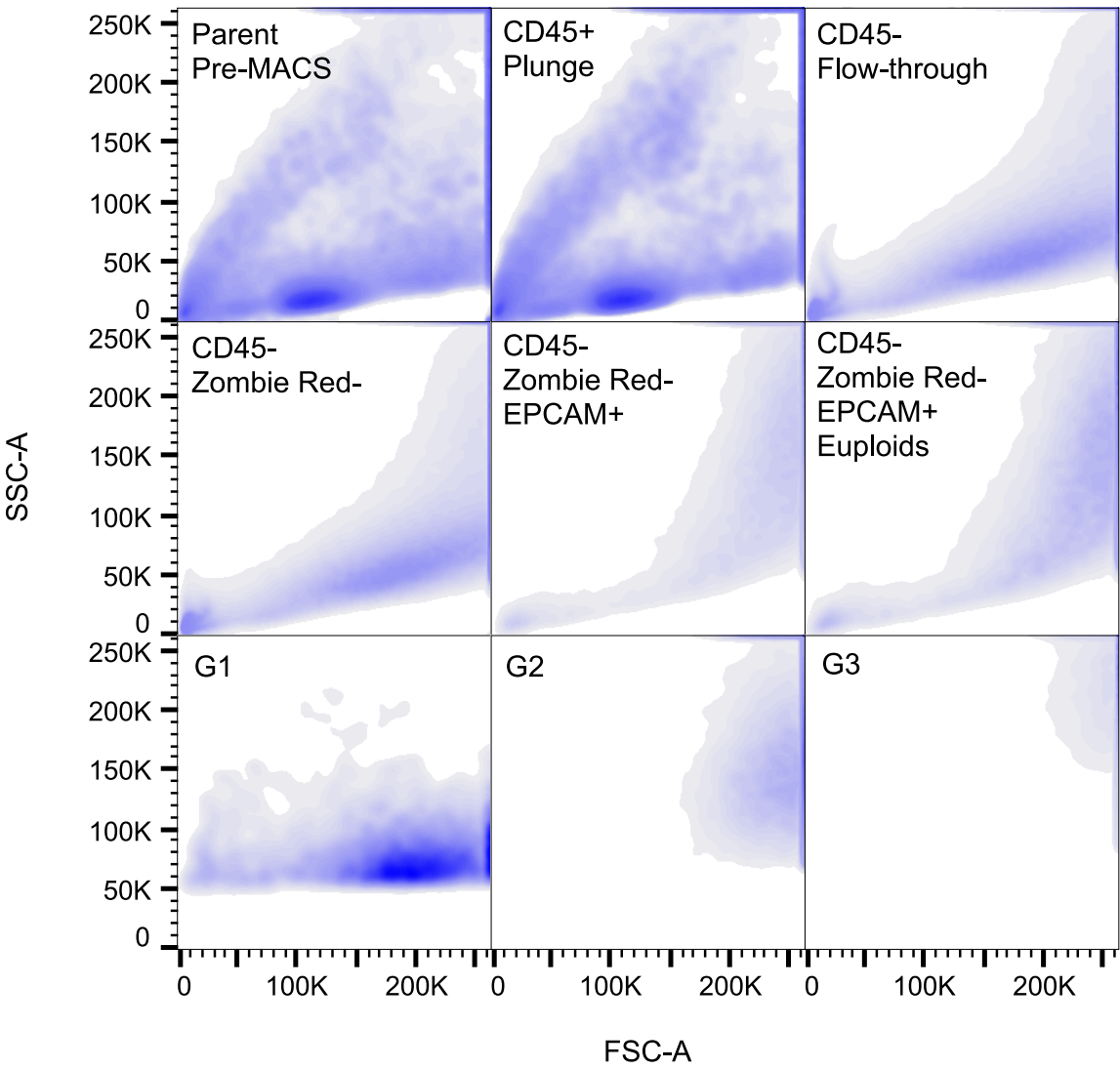

Supplement: S18 Fig — Parent, the digest stained before MACS CD45+ depletion. Plunge, the column retentate containing CD45+ and some CD45- cells. Flow-through, CD45- cells. Zombie Red -, EPCAM+, Euploids, related to the corresponding gates shown in Fig 5A left-to-right. G1-G3, size gates for SSC-W, related to Fig 5B, show the expected increase in FSC-A and SSC-A with increasing cell size occurs, but data resolution and range is not optimal. (PDF) [file pone.0274091.s018.pdf]

Figure S19

A.

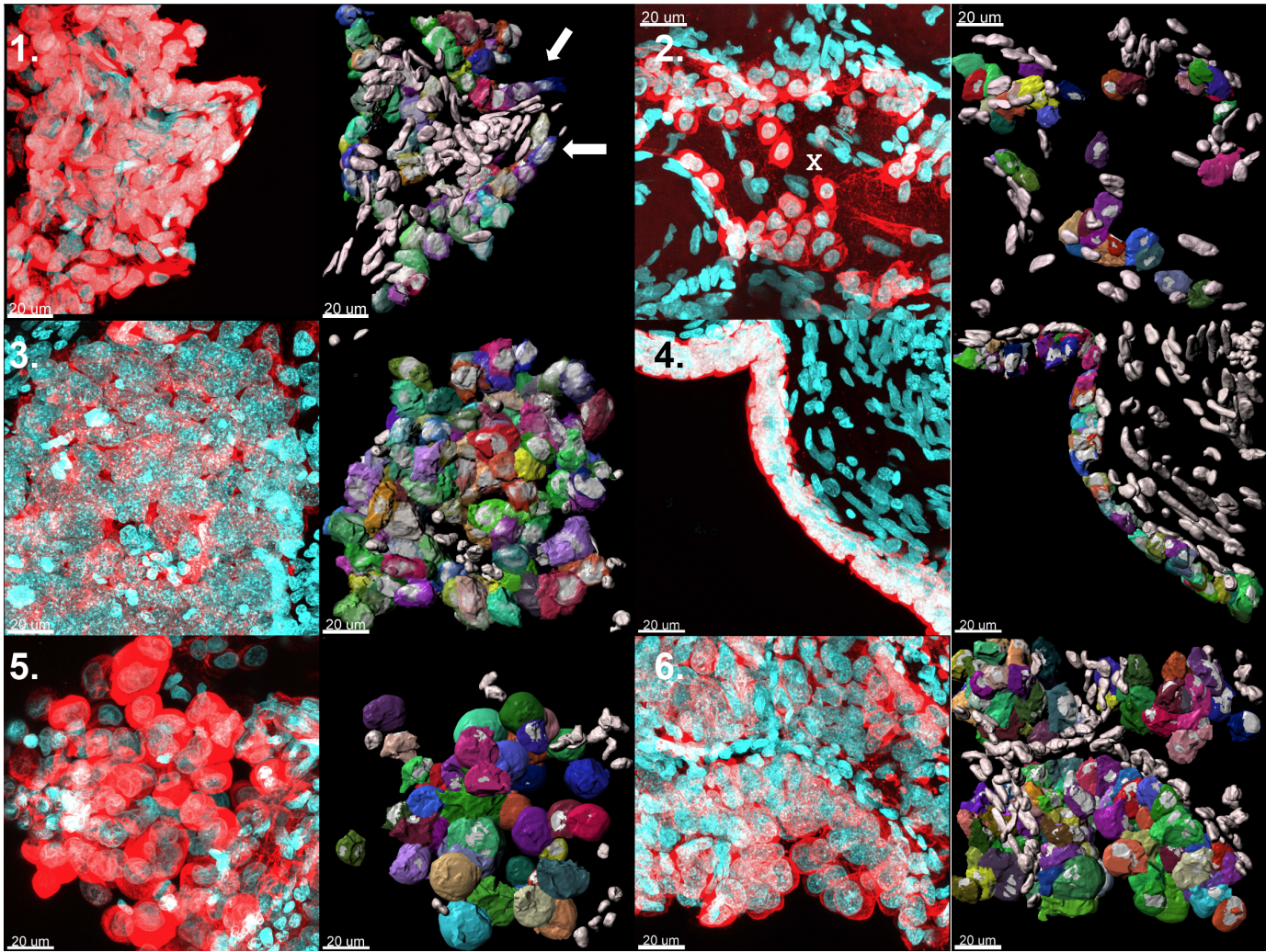

B.

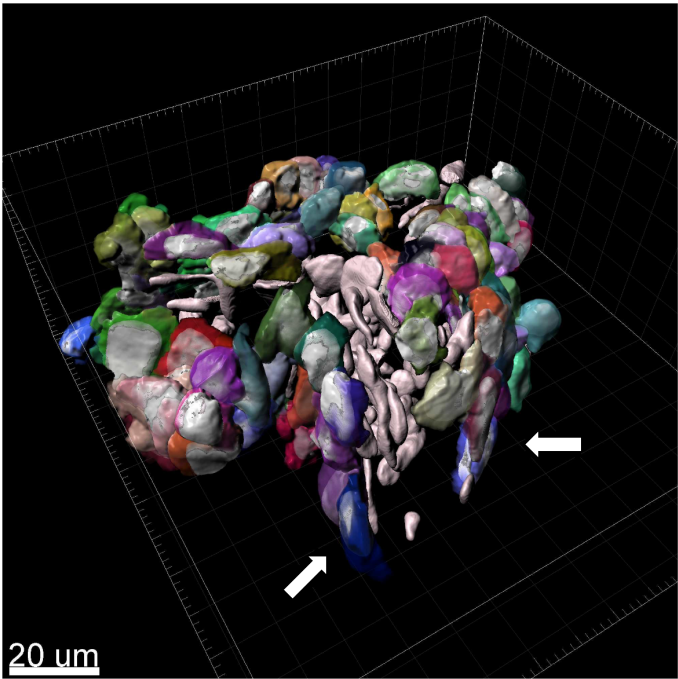

Supplement: S19 Fig — A., z-projections at 63X magnification of flat-looking cells are still many times the thickness of an AT1 cell, suggesting if any developmental programme remains for flattening, it is altered from the wild-type. B., corresponding cell models. Scale, 20 μm. (PDF) [file pone.0274091.s019.pdf]

Figure S20

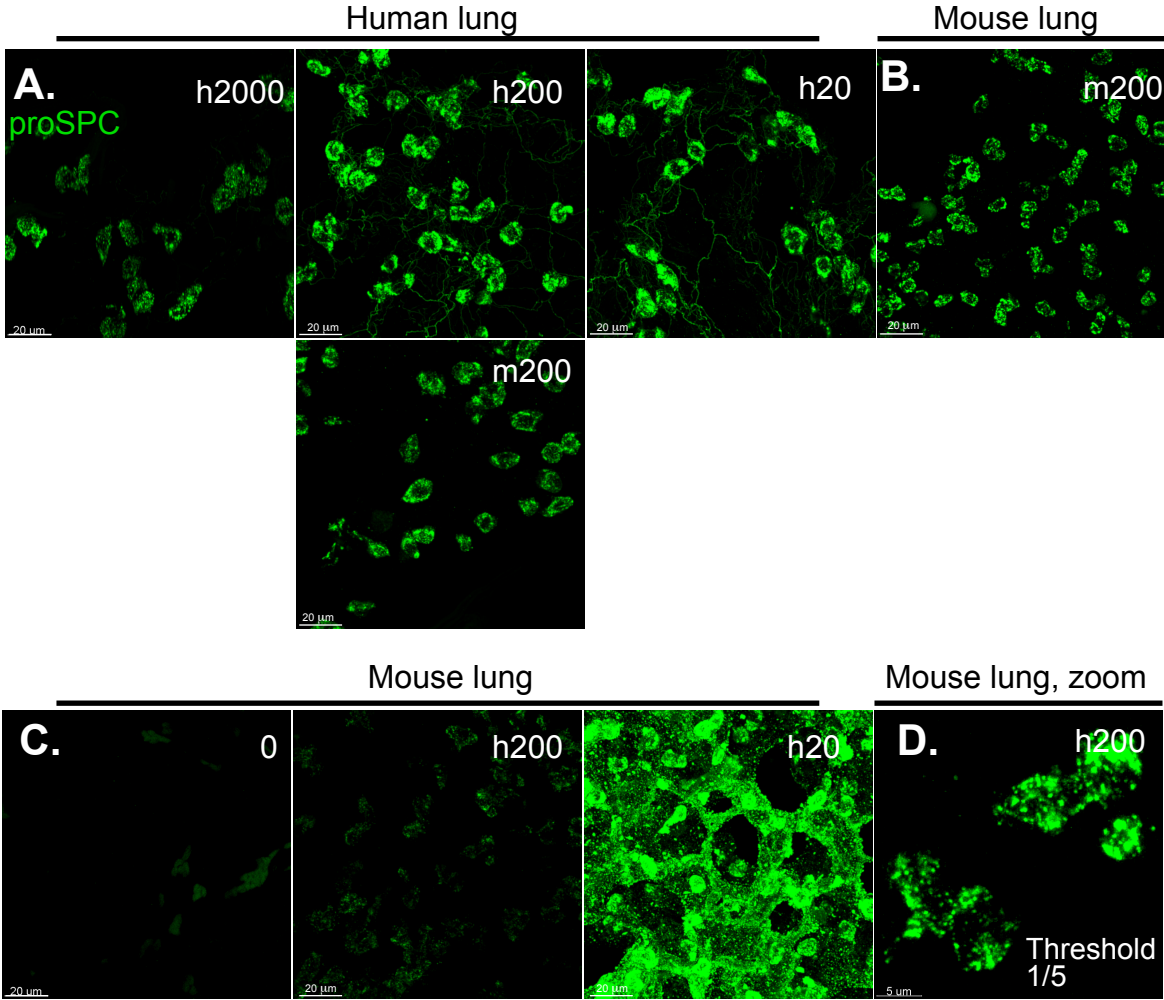

Supplement: S20 Fig — Green, proSPC. h, rabbit antibody ab90716 (abcam) raised against residues 1–100 of human proSPC. m, rabbit antibody ab3786 (Millipore Sigma) raised against residues 1–32 of human proSPC, but previously used in mouse studies. A., antibody and concentration (2000, 200, 20 show dilutions) dependence of IF signals from process networks. Antibody h showed minimal background staining even at high concentration, but nonetheless showed some evidence of processes even at low concentration. The m antibody could not detect processes in humans at the standard 200:1 concentration. B., process networks are not visible in mouse lung using the antibody raised against residues 1–32 at the standard 200:1 concentration. C., titration of the h antibody in mouse, showing that no process-like structures are visible in mouse even with nonspecific binding. D., a zoomed-in view of the C., h200 panel with the upper threshold reduced from 255 to 50 units still does not reveal any processes in mouse. Scale bars, 20 μm. (PDF) [file pone.0274091.s020.pdf]

Figure S21

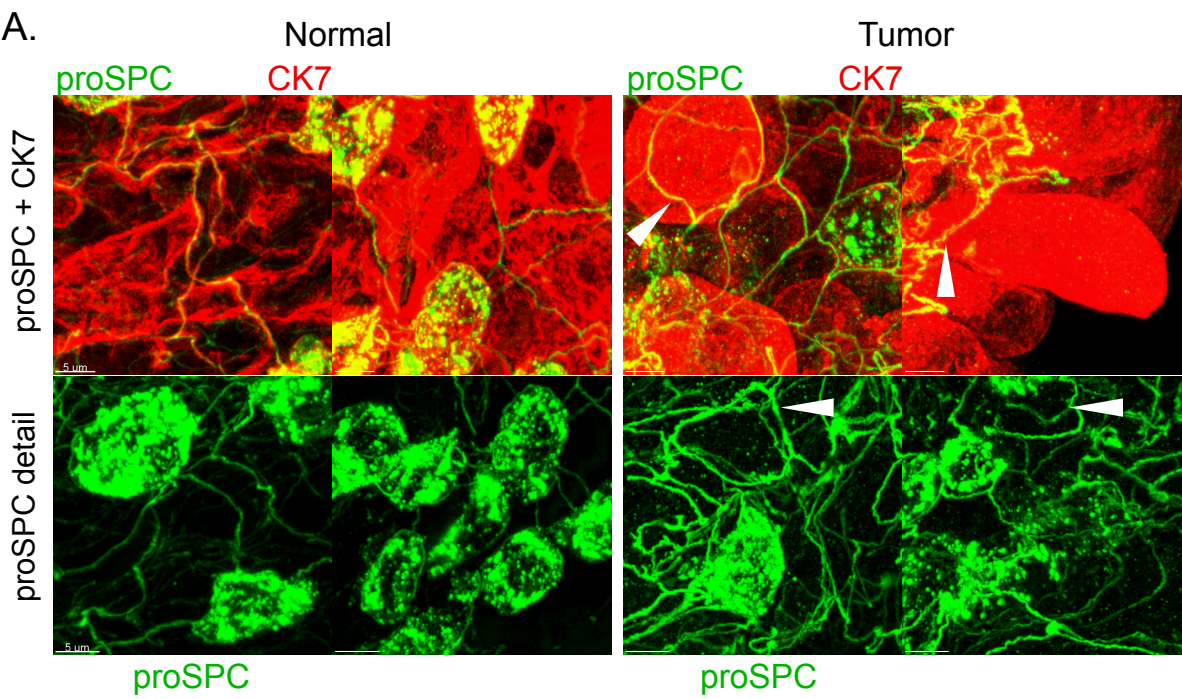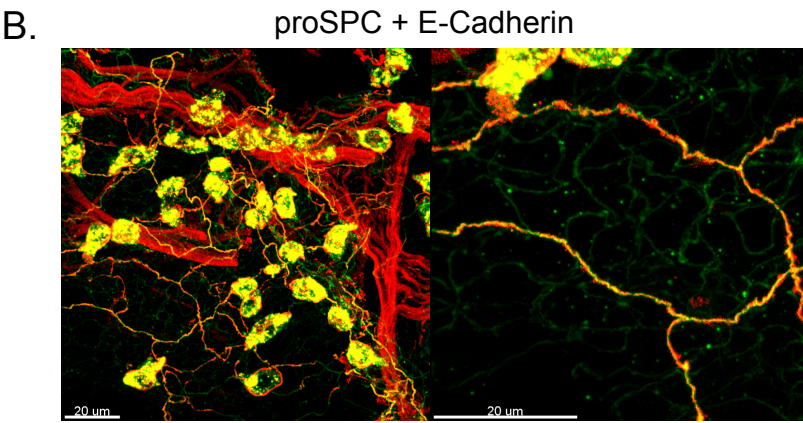

Supplement: S21 Fig — A., zoom views of proSPC, green, and CK7, red, showing how process networks follow the network of intracellular cytokeratin without displaying consistent colocalization (yellow in merge, relative to green in merge). This behavior may be expected in the case of multilamellar vesicles, structures which explain the puncta observed in AT2 cell bodies. White arrows, the distinctive pathology of AT2 processes in tumor cells is a progressive depletion of multilamellar bodies (puncta) and enhancement at cell-cell junctions. Neighbors often display a gradient in vesicle expression, from cells that appear AT2-like, to those with weak process staining and aberrant-appearing paths (see also, S4 and S5 Videos). Scale, 5 μm. B., colocalization of proSPC, green, and E-cadherin, red, is partial at the cell boundary, but “subnetworks” and what may be terminating branches are apparent (right image, darker regions). The “loop” that is visible is typical of the shape of large, flat, AT1 cell bodies in these alveoli. Scale, 20 μm. (PDF) [file pone.0274091.s021.pdf]

Figure S22

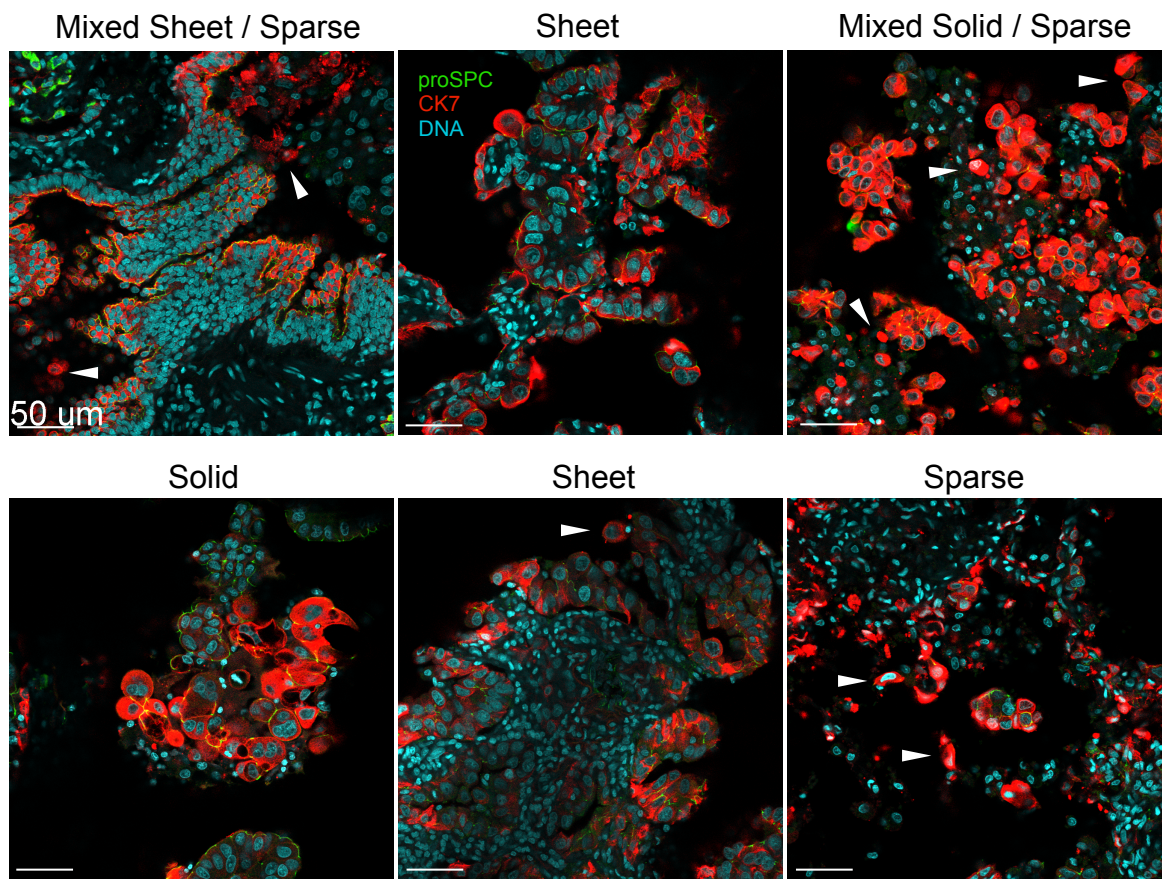

Supplement: S22 Fig — 25x single images illustrating how variations in local cell-cell contact relate to variations in process phenotypes in LA. Sparse tumor cells, arrows, lack process-like proSPC expression relative to their more well-packed neighbors. green, proSPC, red, CK7, cyan, DNA. Top-left, top-center, and lower-right, EGFR+. Top-right, lower-left, and lower-center, KRAS+ (See Table 5 for all Patient ID associations). Scale, 50 μm. (PDF) [file pone.0274091.s022.pdf]

Figure S23

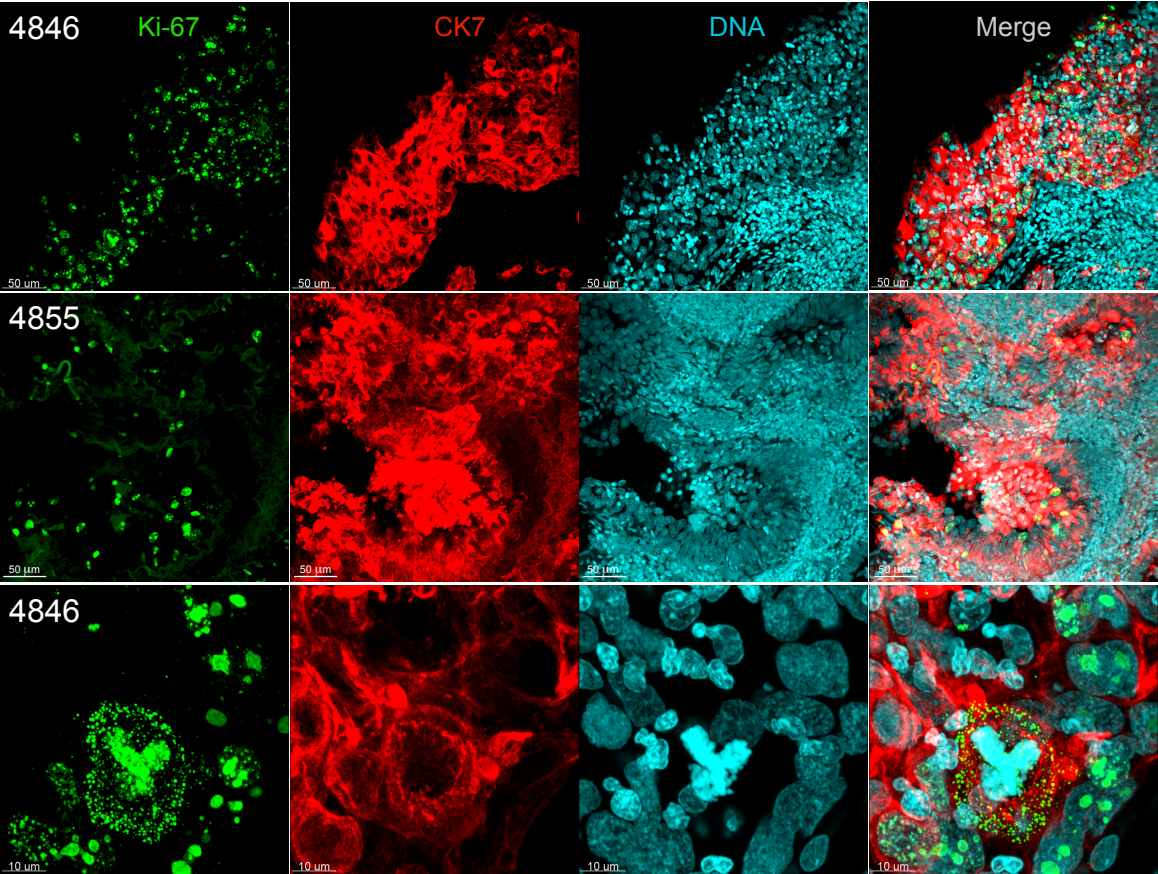

Supplement: S23 Fig — KRAS+ patient ID’s as indicated, with behaviors shown observed in both genotypes. Green, Ki-67, red, CK7, cyan, DNA. Bottom row, a higher density scan of the sample for patient 4846 displays the structural details of unusual proliferation centers in dividing tumor cells of varying size and DNA content. Scale, 50 μm, top and middle rows, 10 μm, bottom row. Normal AT2 cells have such a small fraction of proliferating cells that they were undetectable in the objective. (PDF) [file pone.0274091.s023.pdf]

Figure S24

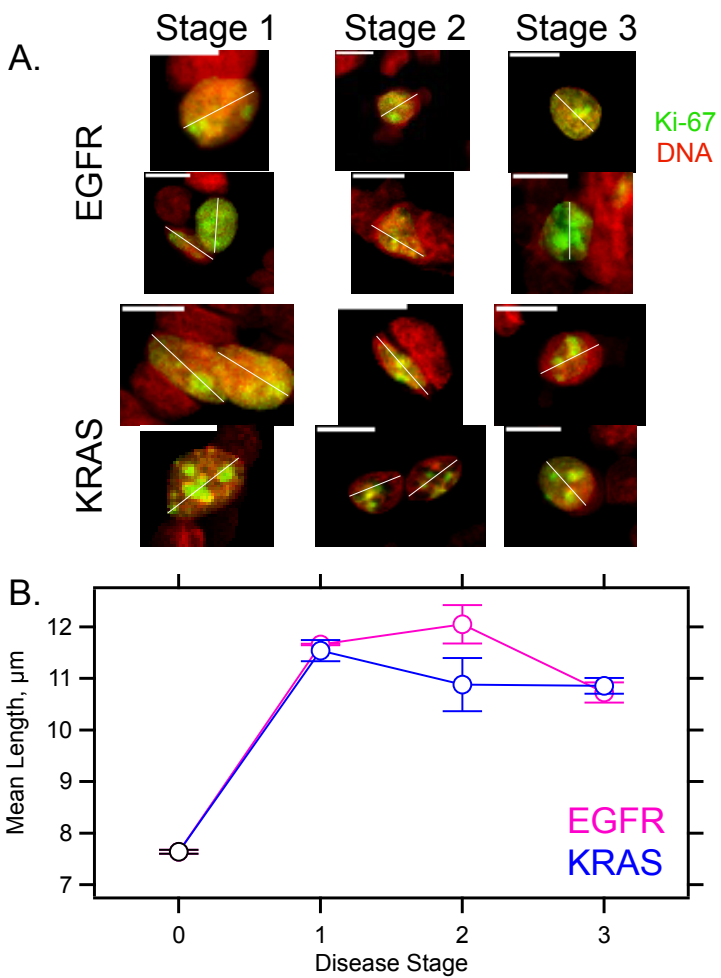

Supplement: S24 Fig — An alternative 2D-based approach was implemented as an expedient way to ascertain whether tumor nuclei classified as proliferating were also longer than normal AT2 nuclei. A., 2D projections of proliferating nuclei, illustrating typical measurement of the long axis, with dimensions near the means shown in B (see Methods). Green, Ki67, red, DNA.” Each row’s images are from a different patient. Scale bars, 10 μm. B., Mean long axis of 2D projections of proliferating nuclei as a function of genotype and disease stage. Black point, normal AT2 nuclei, which proliferate minimally in healthy tissue. Error bars, SEM. (PDF) [file pone.0274091.s024.pdf]

Figure S25

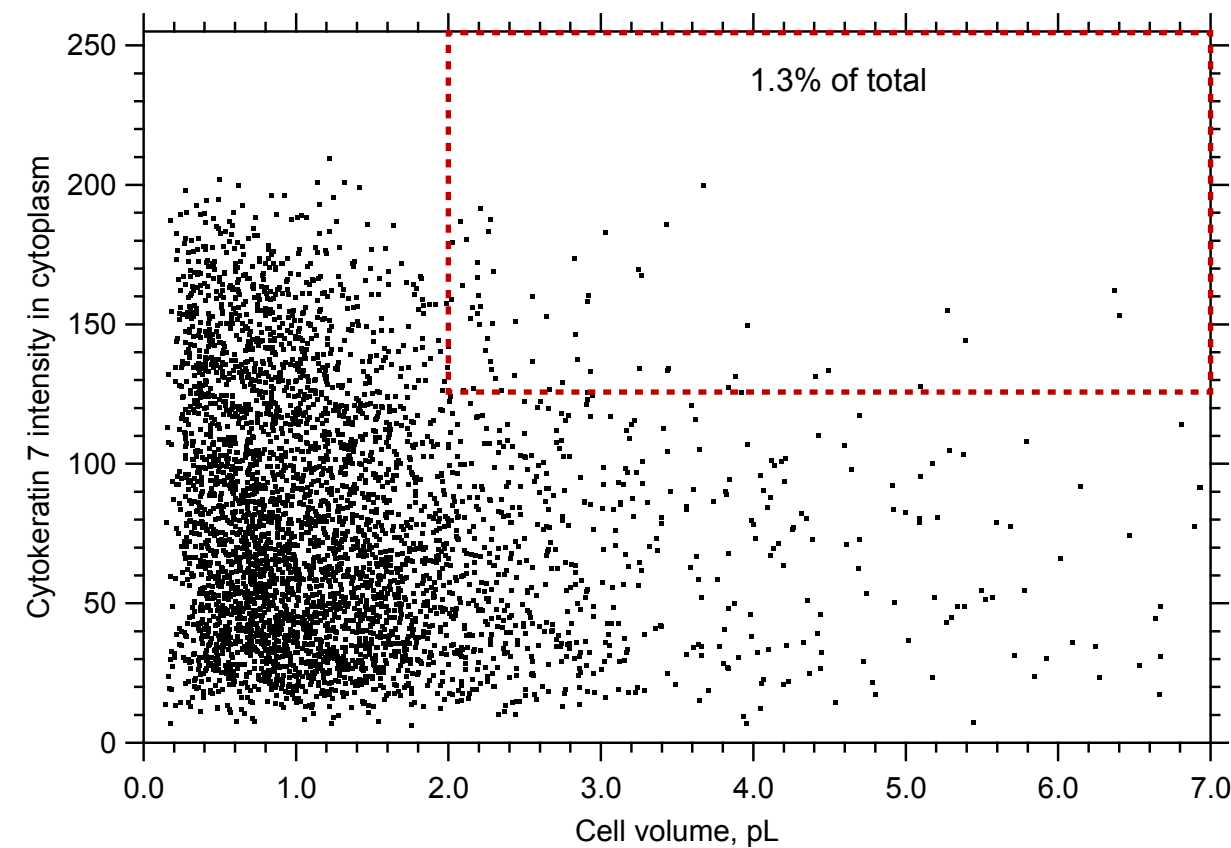

Supplement: S25 Fig — X-axis, intensity of CK7 IF measured in the cytoplasm of cells, employed as a proxy for concentration of intermediate filaments, expected to scale with cell size [22]. X-axis, cell volume. Inset, region discussed in text that is depleted of data, >127.5 A.U. CK7 in cytoplasm and >2 pL volume. 15.5% of data points shown are >2 pL, whereas only 1.3% of all data points are in the region shown (only 8.3% of cells >2 pL). In contrast, 18.2% of cells ≤ 2 pL display such high intensities. n = 4020, from analysis in S1 Data, Tab I. (PDF) [file pone.0274091.s025.pdf]

Figure S26

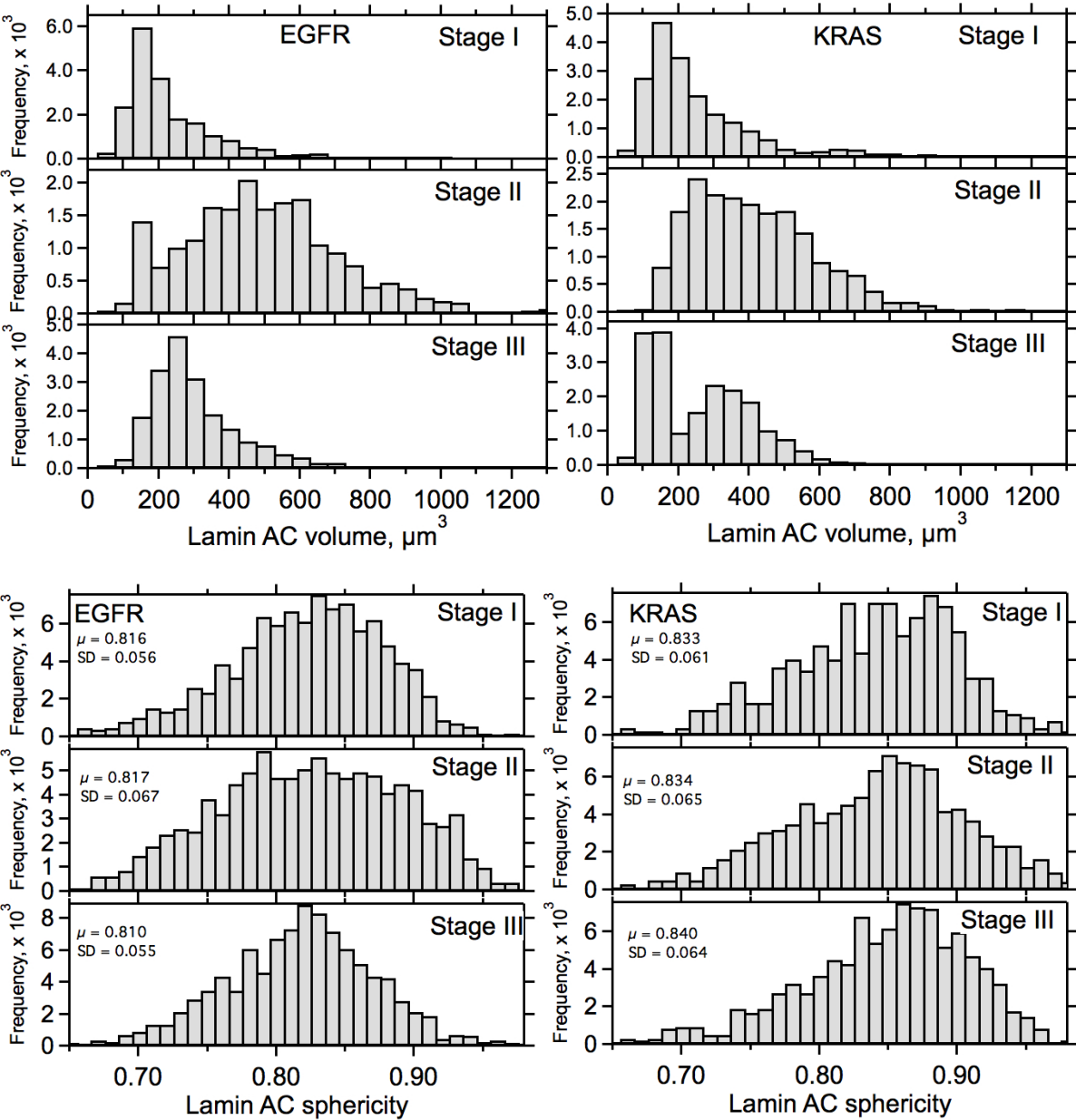

Supplement: S26 Fig — Top, histograms of volumes of nuclei, as reported by direct 3D segmentation of available Lamin A+C staining using Imaris software (S5 Fig, white surface cutaway). Data is of slightly smaller magnitude than that from DNA thresholding, but also supports specific enlargement of nuclei at stage 2 (S7 Fig, see Methods and S1 Data, tab G). Bottom, Lamin A+C surface segmentation effectively captures shape information about nuclear envelopes, as indicated by the reported sphericity, x-axis, as a function of genotype and stage. EGFR+ stage III notably displayed lower mean ± SD sphericity (μ = 0.810 ± 0.055) than other treatments (all other μ ≥ 0.816) with EGFR+ showing lower mean sphericities in all stages than KRAS+ patients (see panels). EGFR,+ left, and KRAS+, right, with disease stages as shown. Sample sizes: N, biological replicates, n, number of envelopes segmented. N = 21, n = 5668. NEGFR = 10, nEGFR,S1 = 1272, nEGFR,S2 = 807, nEGFR,S3 = 1132, NKRAS = 11, nKRAS,S1 = 524, nKRAS,S2 = 974, nKRAS,S3 = 959. (PDF) [file pone.0274091.s026.pdf]

Figure S27

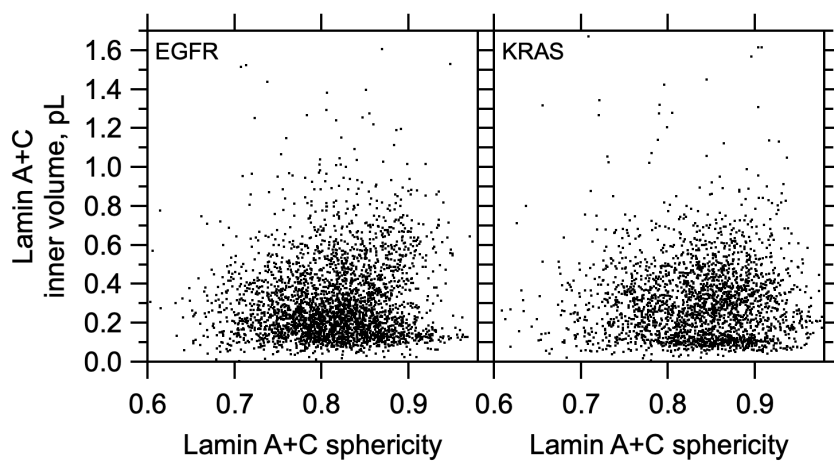

Supplement: S27 Fig — Related to S26 Fig, plots of the volume of the surface segmentation of Lamin A+C staining as a function of the sphericity reported by Imaris software, binned by genotype. Left, EGFR+, right, KRAS+. Lamin A+C Inner Volume = the volume of the envelope built from the Lamin A+C channel, which is significantly smaller than the volume of the nucleus as calculated from DNA thresholding (S5 Fig and Methods). Both large and small nuclei display a range of sphericities, which is not consistent with rounding of nuclei due to nuclear swelling. (PDF) [file pone.0274091.s027.pdf]

Figure S28

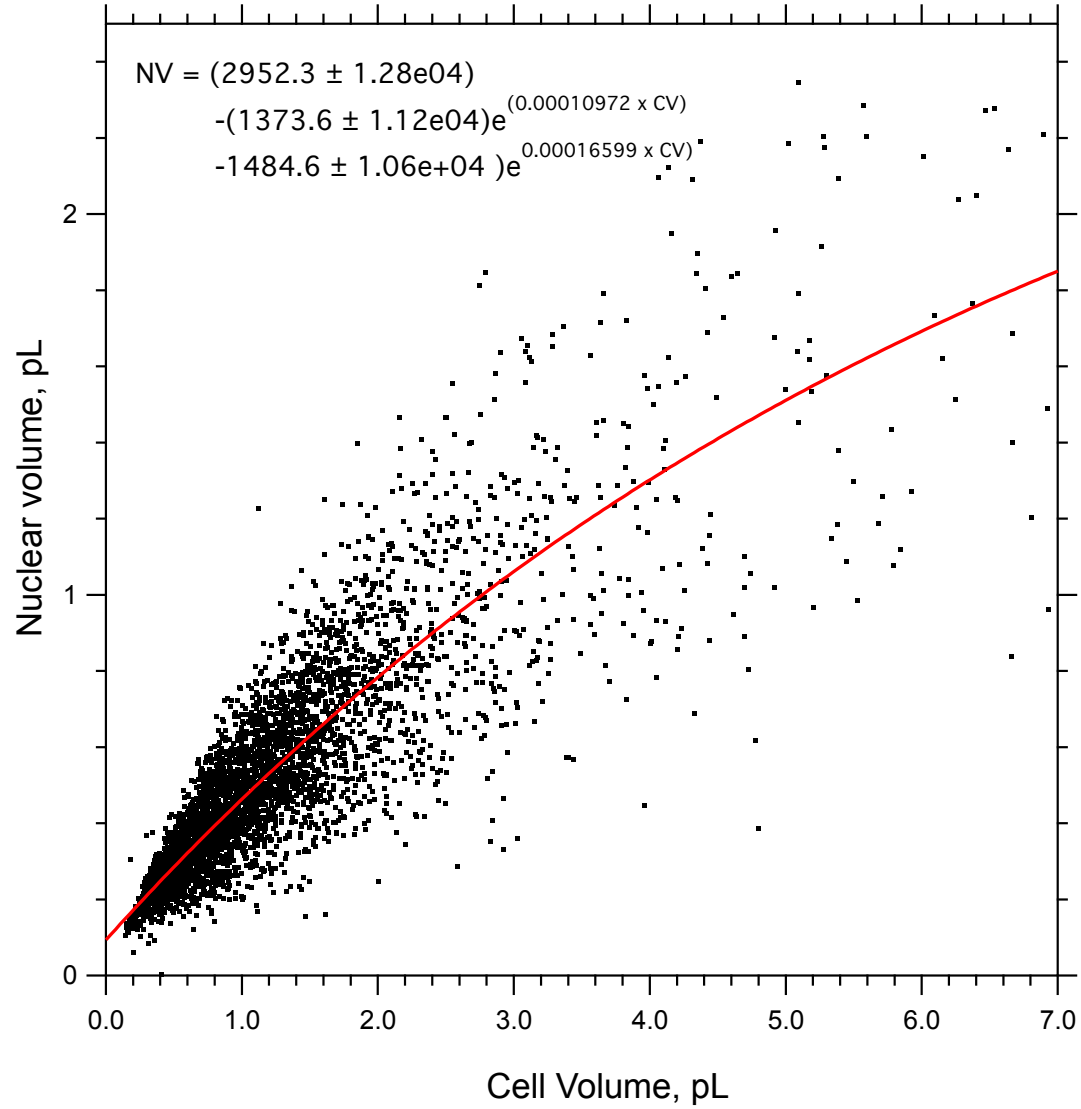

Supplement: S28 Fig — This figure is an enlargement of main Fig 7C. Red line, double exponential fit to data, with the equation and fit parameters shown on the plot. N = 21, n = 4082 Because cell volume mostly explains nuclear volume, organelle scaling is a feature of LA. (PDF) [file pone.0274091.s028.pdf]

Figure S29

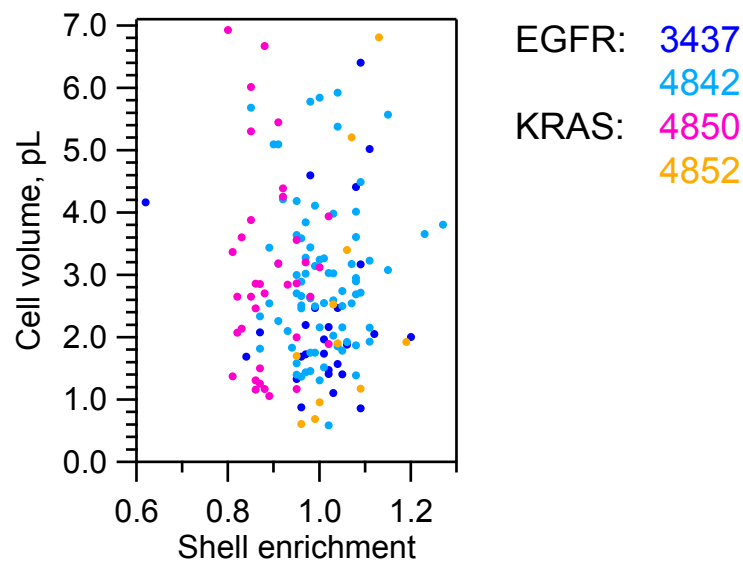

Supplement: S29 Fig — X-axis, shell-enrichment, was defined as the calculated mean intensity of fluorescence within 1.5 μm of the surface defined by the tumor cell nuclear model, divided by the mean intensity not in this outer shell. DNA enriched in the shell does not explain cell volume. Colored dots, data from individual patients. R2: 3437, 6.46 x 10^−5^, 4842, 6.58 x 10^−5^, 4850, 5.65 x 10^−3^, 4852, 0.25. However, shell enrichment overall differs between patients. p-values less than 0.05 from Student’s t-test: 3437 vs 4850, p = 5.4 X 10−7. 4842 vs 4850, p = 1.05 x 10^−12^, 4850 vs 4852, p = 3.6 x 10^−9^. (PDF) [file pone.0274091.s029.pdf]

Figure S30

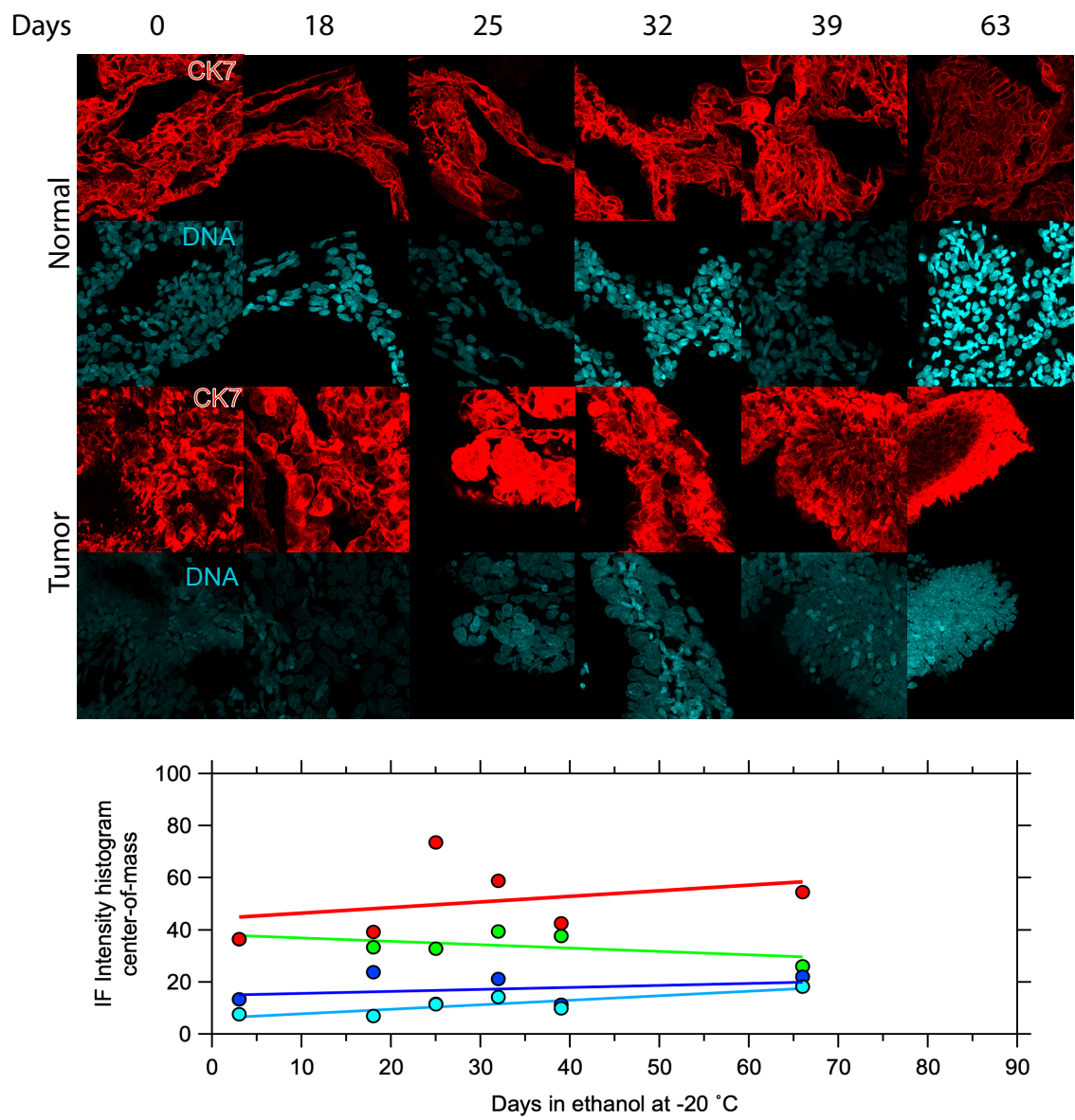

Supplement: S30 Fig — A., Images for control patient 4844 capture typical variability observed with immunofluorescence. Red, CK7, cyan, DNA. B., kinetics of overall stack intensities, as measured by the center-of-mass of the pixel intensity histogram, neither significantly decreased nor increased over the a two-month period when tissue sections were maintained dehydrated and at freezing temperatures. (PDF) [file pone.0274091.s030.pdf]
