# Supplementary material for: Epithelial cell size dysregulation in human lung adenocarcinoma
Source: PLoS One. 2022 Oct 6;17(10):e0274091. doi: 10.1371/journal.pone.0274091 (PMC9536599; doi:10.1371/journal.pone.0274091)

Figure S3

A. Automatic modeling of surfaces using Bitplane Imaris vs. UNet predictions and marked nuclear centers.

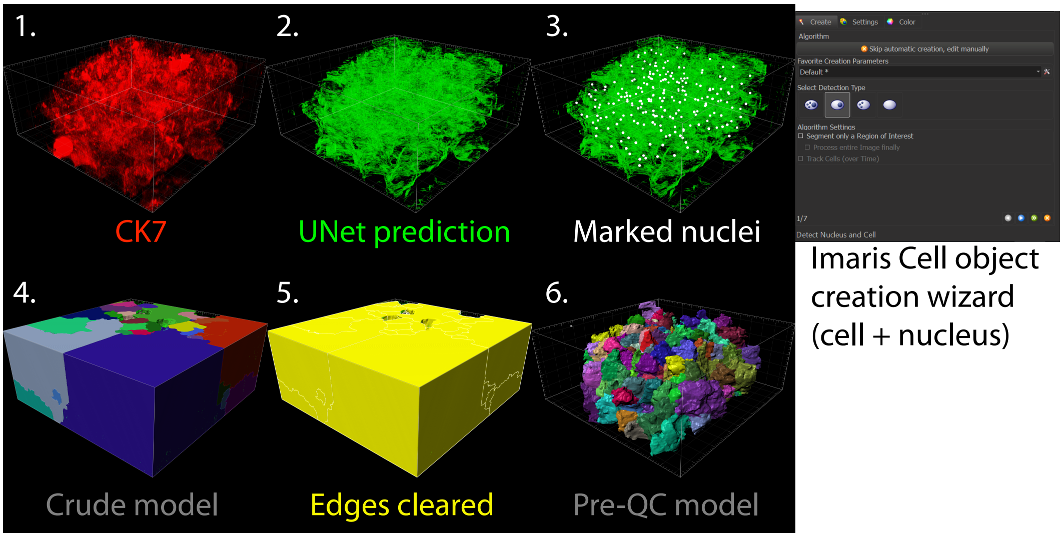

B. Manual quality checking guided by data and nuclear segmentation

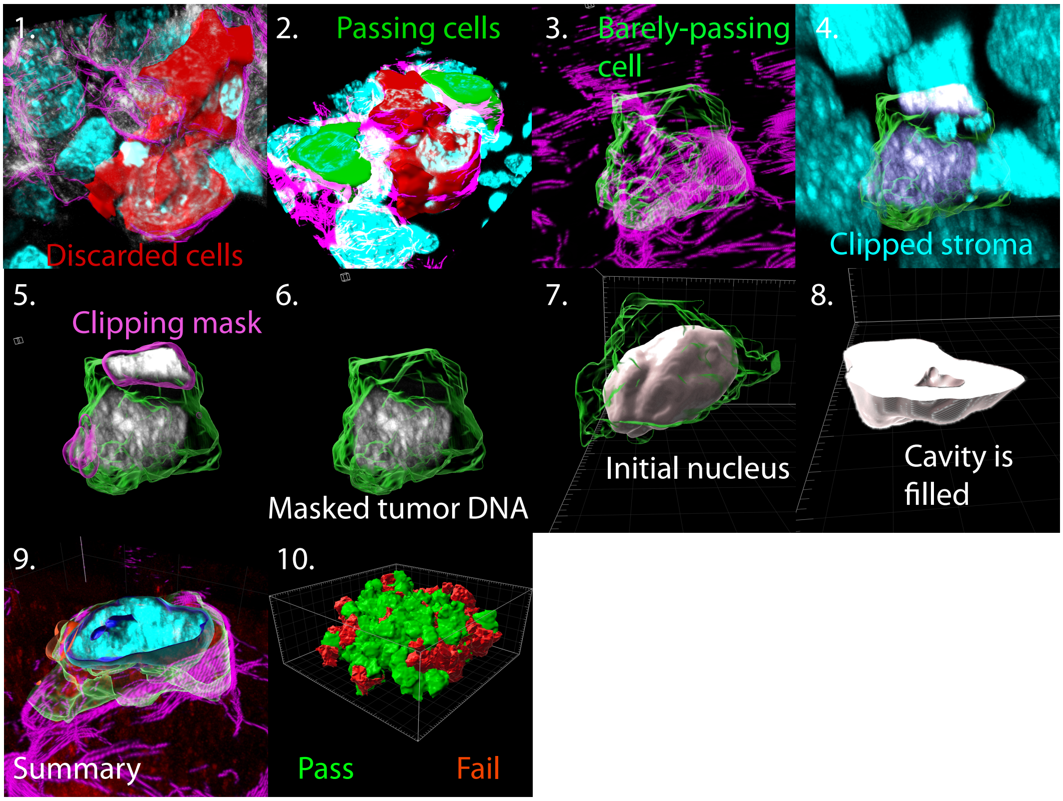

Supplement: S3 Fig — See Methods for detailed walkthrough. A: 1., CK7 data which was annotated by hand was used to train UNet, which, 2., predicts cell boundaries at every slice. 3., centers of nuclei are marked and transformed to spherical white regions to provide an idealized nucleus to guide the Imaris fitting algorithm. 4., Imaris fits the UNet-predicted “ribs” of each cell model. 5., Imaris creates volumes out the edges of the 3D stack volume, which are deleted. 6., The initial Imaris model contains both correct and incorrect cell models. B: 1., nuclear segmentation and all available IF staining and UNet predictions were used to assess the accuracy of every cell model in the X, Y, and Z view using clipping planes in Imaris. 2., cell models which disagreed with IF or UNet channels in Imaris or obviously conflicted with nuclear segmentation by >10% volume were discarded. 3., example of a lower-quality cell model which barely passed agreement with manual curation, presenting clipping of the cell body model with neighboring stromal nuclei. 4., view of the conflict in the DNA channel that illustrates the lower-limit of acceptance for model quality. 5., to quantify DNA accurately, any DNA clipped into a model were masked, first by creating a volume and then by deleting the data in Imaris. 6., illustration of a low-quality cell model with nucleus containing masked DNA associated uniquely with the cell model. 7., initial nuclear volume rendering, based on initial binary segmentation of the DNA channel made offline in FIJI. 8., a cavity present in the binary DNA channel was frequently seen in the larger nuclei of tumor cells, and was filled-in using FIJI to eliminate the void (related to Fig 8A). 9., after curation, overall agreement between Imaris models of cell bodies and nuclei, taken together with UNet and DNA channels is re-assessed. Blue and white, insignificant changes in volume and shape occurred after cavity-filling using FIJI. 10., typical overall appearance of coverage of cell mo [file pone.0274091.s003.pdf]
